# Diastereoselective and Chemically Reversible C–C Bond Formation Mediated by an (N-heterocyclic)boryloxy Aluminyl Compound

**DOI:** 10.1021/jacs.6c03691

**Published:** 2026-05-20

**Authors:** Debotra Sarkar, Petra Vasko, Job J. C. Struijs, Maximilian Dietz, Simon Aldridge

**Affiliations:** † Inorganic Chemistry Laboratory, Department of Chemistry, 6396 University of Oxford, South Parks Road, Oxford OX1 3QR, U.K.; ‡ Department of Chemistry, 37268 Indian Institute of Technology Madras, Chennai 600036, India; § Department of Chemistry, 3835 University of Helsinki, A.I. Virtasen Aukio 1, P.O. Box 55, Helsinki FI-00014, Finland

## Abstract

We report on the reductive C–C coupling of alkenes at a single main-group-metal center. Treatment of the bis­(boryloxy)­aluminyl compound K­[Al­{OB­(NDippCH)_2_}_2_] with ethene results in clean formation of a five-membered alumina-cyclopentane via alkene dimerization. In the case of propene, similar chemistry occurs regio- and diastereoselectively to generate the corresponding *rac*-3,4 disubstituted metallacycle. Isolation of an intermediate alumina-cyclopropane and quantum chemical analysis allows the mechanism of these transformations to be elucidated, with coupling between the most hindered carbon centers in propene being driven by the necessity to minimize steric interactions with the bulky aluminum-bound ligand scaffold. Although these transformations are thermodynamically favorable and physically irreversible (no alkene regeneration under vacuum), chemical reversibility can be effected through the addition of small unsaturated molecules (CO, CO_2_, alkynes), which leads to C–C bond scission and the loss of one or both alkene equivalents. These findings expand the reactivity landscape of low-valent Al­(I) reagents and showcase main-group-mediated pathways that parallel classical transition metal chemistry.

## Graphical abstract

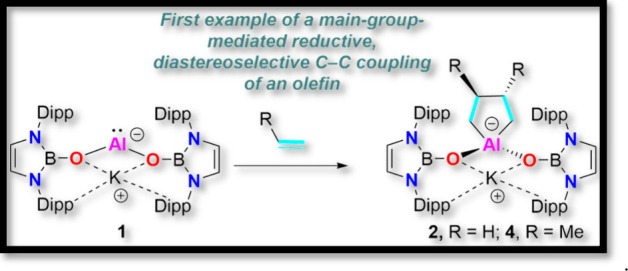

The formation of carbon–carbon (C–C) bonds remains a cornerstone transformation in synthetic chemistry, with transition metal (TM) catalysis occupying a central role due to its unmatched efficiency, selectivity, and mechanistic versatility.^1–5^ Among various TM-catalyzed C–C bond-forming processes, ethene oligomerization holds particular industrial and mechanistic significance, for example in the formation of 1-hexene via the Phillips process (Figure 1a).^3,6–8^

**Figure 1:**
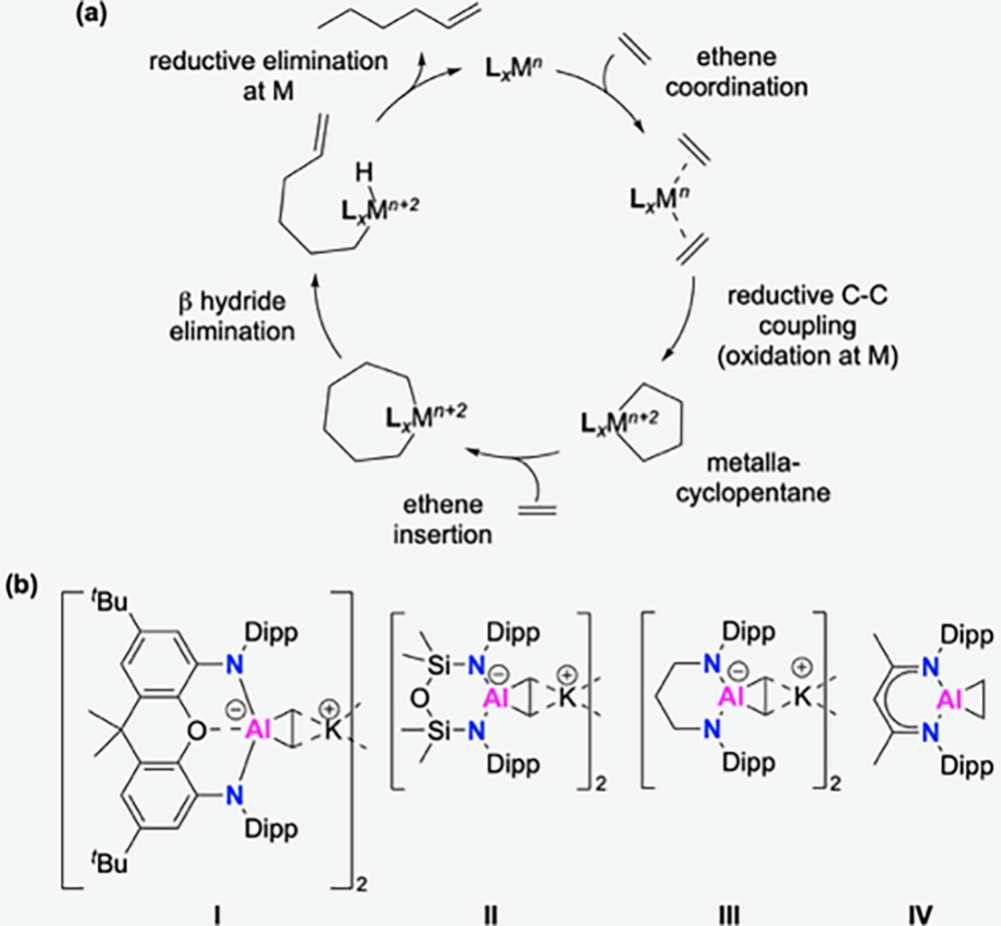
(a) Transition-metal-mediated ethylene oligomerization; (b) Al­(III) metalla-cyclopropane complexes formed via Al­(I)-mediated ethene activation [Dipp = 2,6-diisopropylphenyl].

Recent developments in main-group chemistry have begun to challenge the traditional boundaries in small molecule activation and catalysis.^9–12^ Low-valent *p*-block compounds—once thought incapable of emulating the coordinative flexibility and reactivity of transition metals—have been molded to coax TM-like behavior in a variety of bond activation processes.^9–12^ Among these, nucleophilic aluminyl anions [AlX_2_]^−^, featuring a low-coordinate Al­(I) center that is isoelectronic with a carbene, have emerged as versatile platforms for small-molecule activation.^13–16^ These species possess a stereochemically active lone pair and an orthogonal formally vacant p-orbital, enabling ambiphilic reactivity reminiscent of transition metal complexes.^13–16^ Their pronounced nucleophilicity has enabled the activation of diverse substrates, including the C–H and even C–C bonds in benzene, as well as small molecules such as CO_2_, N_2_O, CO, NH_3_ and H_2_.^13,14,17^ Reactions with alkenes have also been reported, albeit exclusively proceeding via (2 + 1) cycloaddition to form alumina-cyclopropanes (Figure 1b).^16,18–22^

In addition to aluminyl compounds, a variety of other low-valent *p*-block species—including tetrylenes,^23–34^ digermynes,^35,36^ disilynes,^37,38^ distannynes,^39^ distannenes,^40^ digallynes,^41^ dialumenes,^42,43^ neutral Al­(I)^44–46^ and cationic Al­(III) species,^47,48^ and distibenes^49^— have been shown to activate ethene and other alkenes, often leading to 1,2-insertion or (2 + 1), (2 + 2) or (2 + 2 + 2) cycloaddition products. In addition—very recently—the catalytic trimerization of *alkynes* by Al­(I) has been reported, driven thermodynamically by the formation of aromatic benzene products.^50^

Despite these developments, the reductive C–C coupling of alkenes at a single main-group center—akin to key mechanistic steps in TM-catalyzed alkene oligomerization^3,8^ —has not previously been definitively realized.^51^ In an attempt to address this, we have investigated the reactivity of anionic Al­(I) systems toward simple alkenes. Utilizing an N-heterocyclic boryloxy aluminyl compound,^17,52^ we demonstrate C–C bond formation from ethene to give a five-membered aluminacyclopentane, and the extension of this chemistry to propene—which gives rise to a similar coupling product in regio- and diastereoselective fashion.

Exposure of a solution of K­[Al­{OB­(NDippCH)_2_}_2_] (**1**) in C_6_H_6_ to ethene at ambient temperature and pressure results in an immediate color change from yellow to colorless, accompanied by rapid formation of a crystalline material subsequently identified as K­[(C_4_H_8_)­Al­{OB­(NDippCH)_2_}_2_] (**2**) (Scheme 1). Compound **2** has been characterized by multinuclear NMR spectroscopy, single-crystal X-ray diffraction (SCXRD) and elemental microanalysis. Its ^1^H NMR spectrum displays a diagnostic high-field signal integrating to 4H at δ_H_ = −1.47 ppm, i.e. at a chemical shift similar to the signals assigned to the Al–CH_2_ moieties of alumina-cyclopropanes **I**–**IV** (δ_H_ = 0.67 to −1.40 ppm), and consistent with the formation of an Al­(III) metallacycle.^18,19,22,45^ However, in contrast to these systems, the molecular structure determined by SCXRD (Figure 2) reveals a five-membered alumina-cyclopentane ring, formed by the reductive coupling of 2 equiv of ethene at aluminum. This reactivity is reminiscent of classical TM-mediated ethene oligomerization (Figure 1a), and stands in contrast to previously reported Al­(I)-mediated ethene activation, which uniformly involves reaction with one equivalent of the alkene to give a (2 + 1) cycloaddition products.^18–20,45^ More broadly, to our knowledge, this chemistry represents the first definitive example of main-group-mediated coupling of two equivalents of alkene at a single metal center.

**Scheme 1:**
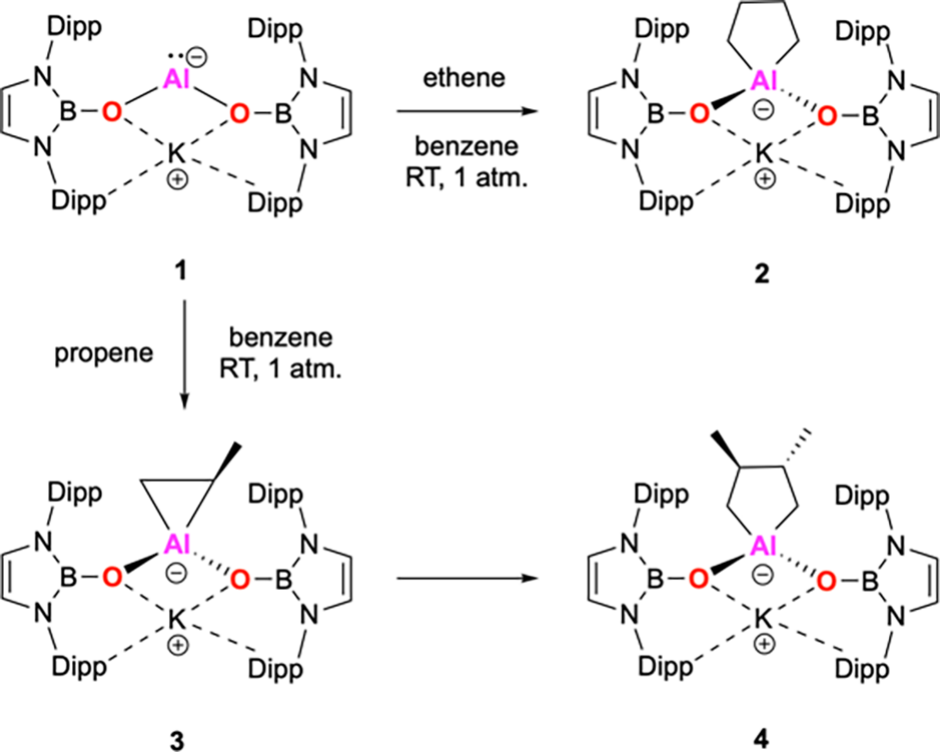
Aluminyl-Mediated Reductive Coupling of Ethene and Propene

**Figure 2:**
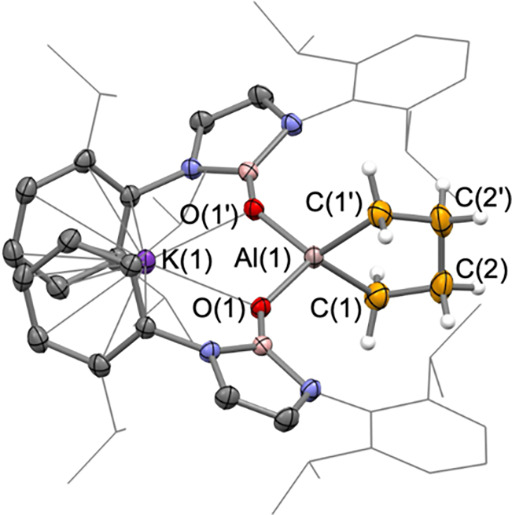
Molecular structure of compound **2** in the solid state as determined by X-ray crystallography. Thermal ellipsoids set at the 50% probability level; selected hydrogen atoms omitted, and ligand carbon atoms shown in wireframe format for clarity.

The symmetry-related Al–C bond lengths in **2** (1.979(1) Å) are similar to those measured for previously reported alumina-cyclopentanes (ca. 1.95 Å) – compounds which are typically synthesized by a double salt metathesis reaction between an Al­(III) halide and a dilithio-alkyl reagent (LiCR_2_CH_2_)_2_.^53^ Geometrically, the aluminum center in **2** is tetra-coordinated by two N-heterocyclic boryloxy (NHBO) ligands and two carbons from the metallacycle. The K^+^ counterion remains encapsulated between the two oxygen atoms of the boryloxy groups, as distinct from compounds **I**–**III**, which are dimeric and feature K^+^ bridging between aluminacycle units.^18,19,22^ With this in mind—and to assess the specific role of the counterion—we examined the corresponding reactivity of “naked” aluminyl compound [K(2.2.2-crypt)][Al{OB(NDippCH)_2_}_2_] (**1′**), in which the K^+^ cation is sequestered by 2.2.2-cryptand. Exposure of **1′** to ethene under comparable conditions yields the analogous product [K(2.2.2-crypt)][(C_4_H_8_)Al{OB(NDippCH)_2_}_2_] (**2′**), a charge-separated aluminacyclopentane—as confirmed by SCXRD (Figure S25), albeit less cleanly and in lower yield compared to **2** (36% vs 82%). These observations suggest that the availability of K^+^ plays at least some role in the reaction pathway for C–C bond formation in this system, consistent with trends observed in several previously studied systems.^19,52^

To probe the broader scope and selectivity of this aluminum-mediated C–C coupling chemistry, we examined the reactivity of compound **1** toward propene. Under conditions similar to those used with ethene, the reaction affords the corresponding five-membered-ring product K­[(C_4_H_6_Me_2_-3,4)­Al­{OB­(NDippCH)_2_}_2_] (**4**) in 72% yield (Scheme 1). The alumina-cyclopentane unit in **4** results from the regio- and diastereoselective coupling of two equivalents of propene. The solid-state structure determined by SCXRD confirms that the Me substituents are found in the 3- and 4-positions of the AlC_4_ heterocycle, i.e., remote from the sterically encumbered [Al­{OB­(NDippCH)_2_}_2_] unit. The heterocycle features an *anti* configuration of the methyl groups, in which both substituents occupy pseudoequatorial positions within a chair-like conformer. Notably, this type of regioselective reductive coupling of α-olefins (such as propene) remains a challenge even for transition metal complexes.^54–56^

Further investigation of the reaction of **1** with propene by *in situ* NMR monitoring suggests the formation of a transient intermediate, subsequently shown to be the alumina-cyclopropane species, K­[(C_2_H_3_Me)­Al­{OB­(NDippCH)_2_}_2_], **3**, formed by uptake of a single equivalent of propene (Scheme 1). Prompt workup and crystallization enabled isolation of **3**, albeit as a mixture of crystals (with **4**), leading to very poor isolated yield (3%). Nonetheless, the structure of **3** can be determined crystallographically (Figure 3) showing it to feature a three-membered alumina-cyclopropane ring similar to those reported for compounds **I**-**IV** (Figure 1), albeit with a pronounced divergence in the Al–C bond lengths (*d*(Al­(1)–C­(5) = 1.903(6) Å; *d*(Al­(1)–C­(5) = 2.056(7) Å; *d*(C­(4)–C­(5) = 1.629(9) Å). This unsymmetrical ligation ultimately proves to be important mechanistically, influencing the selectivity in C–C coupling (see below).

**Figure 3:**
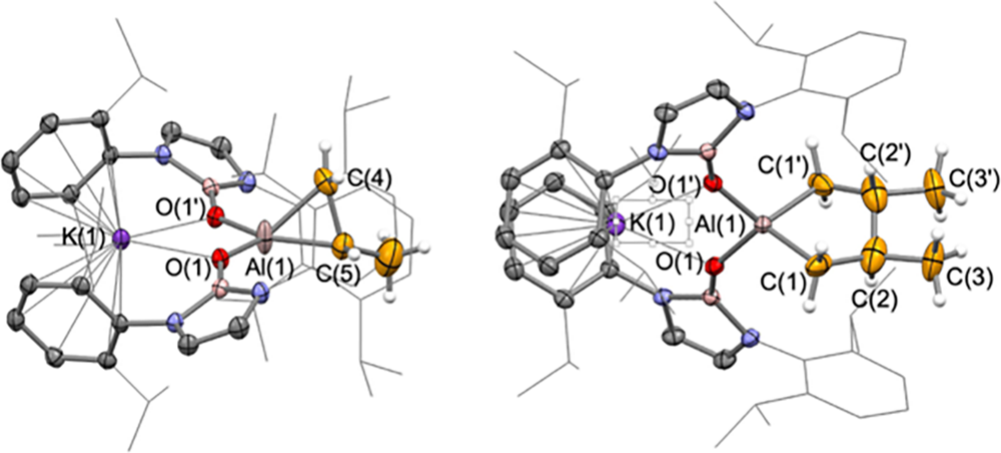
Molecular structures of compounds **3** (left) and **4** (right) in the solid state as determined by X-ray crystallography. Thermal ellipsoids set at the 50% probability level; second disorder component and selected hydrogen atoms omitted, and ligand carbon atoms shown in wireframe format for clarity.

From a broader perspective, and in contrast to compound **IV**,^45^ none of compounds **2**-**4** releases alkene at room temperature under high vacuum, indicating a lack of *physical* reversibility under such conditions. Heating at 100 °C ultimately leads to decomposition into complex mixtures, indicating limited thermal stability at elevated temperatures.

In order the probe (i) the preferential formation of five-membered alumina-cyclopentane **2** in the reaction of **1** with ethene; (ii) the regio- and diastereoselectivity in the coupling of propene by **1**, we carried out mechanistic calculations using Density Functional Theory (Gaussian16Rev.C02, PBE0-GD3BJ/Def2-TZVP­(PCM, benzene)//PBE0-GD3BJ/Def2-SVP) using the complete molecular scaffolds of compounds **1**-**4** (including K^+^ counterion). In terms of the C–C coupling of ethene, the reactions of aluminyl compound **1** with 1–3 equiv of ethene to give 3-, 5- or 7-membered metallacycles are found to be sequentially more thermodynamically favorable (by 40.5, 224.6, and 268.7 kJ mol^–1^, respectively; Figure 4). The activation barriers associated with assimilation of successive ethene molecules, however, become sequentially higher (64.2, 95.0, and 273.2 kJ mol^–1^ for TS1–3, respectively), reflecting (at least in part) increased steric crowding at the aluminum center. In addition, uptake of the second ethene molecule is (uniquely) characterized by involvement of the potassium counterion, which is elevated out of the AlO_2_ plane to establish a contact with the remote carbon of the existing CH_2_CH_2_ ligand (ca. 3.5 Å in TS2). Polarization of the C_2_ unit in this manner would be expected to facilitate C–C bond formation by electron donation from the incoming (second) alkene molecule (Figure 4, inset). This mechanistic proposal is also consistent with the cleaner/higher yielding C–C coupling effected experimentally by **1** (over [K­(2.2.2-crypt)]^+^ system **1′**).

**Figure 4:**
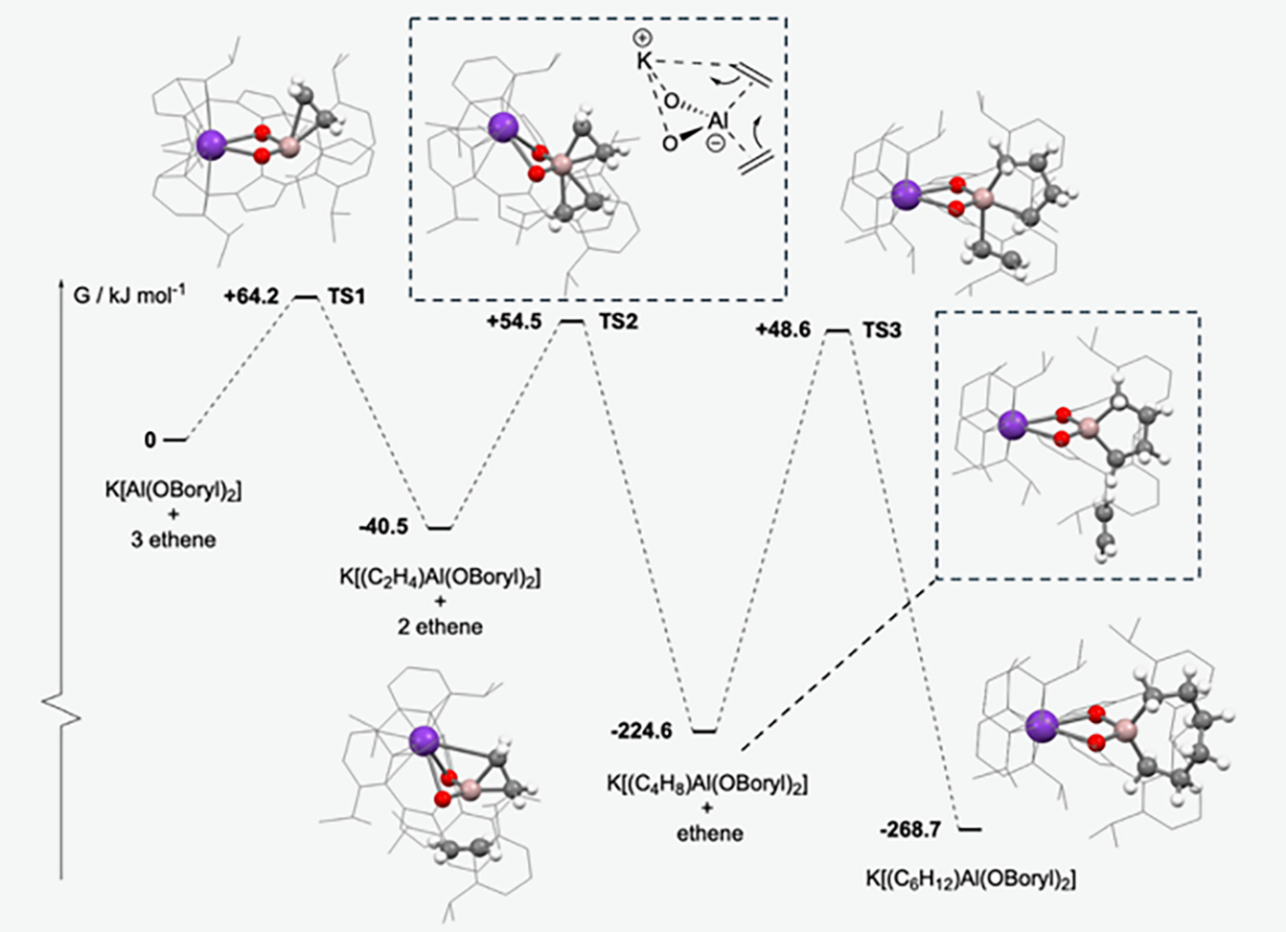
DFT-calculated mechanism for the coupling of ethene by aluminyl compound K­[Al­{OB­(NDippCH)_2_}_2_] (**1**).

Similar calculations on the mechanism of propene coupling show that the product formed (**4**)—which involves C–C bond formation between the two most sterically encumbered alkene carbons—is favored both thermodynamically and kinetically (Figure 5). This isomer (with the Me groups in the 3- and 4-positions of the metalla-cyclopentane ring) is more stable than the alternative 2,4- and 2,5-regio-isomers by 17.9 and 39.7 kJ mol^–1^, respectively. Kinetically, the transition state associated with the formation of the 3,4-isomer from the corresponding metalla-cyclopropane (**3**) is also significantly lower than those associated with either of the alternative regio-isomers—each of which requires alignment of the incoming propene Me group *toward* the sterically encumbered aluminum center (ΔG^⧧^ = 67.4 vs 78.5 and 108.0 kJ mol^–1^). While steric factors clearly influence the orientation of the *incoming* propene molecule, the coordination geometry at the aluminum center in intermediate **3** (as determined both crystallographically and quantum chemically; Figure 3 and the Supporting Information (SI)) appears to favor C–C bond formation involving the more hindered carbon of the *existing* metalla-cyclopropane ring. As such, atoms Al(1), O(1), O(1′) and C(5) in **3** define a very close to trigonal plane (sum of angles = 359.5°), with C(4) being projected above the plane, and the approach of the second alkene molecule therefore being more facile from below—favoring C–C formation involving C(5). This pronounced deviation from the expected tetrahedral geometry at Al(1) is presumably a consequence of the greater steric loading at C(5) than C(4) (i.e., C­(Me)H vs CH_2_). Finally, in terms of diastereoselectivity, the formation of **4**, rather than the alternative *meso* isomer is driven by keeping the methyl groups *anti* to one another in the transition state, i.e. is driven by steric repulsions *between* the Me substituents (Figure 6). The transition state associated with C–C coupling to give the alternative *meso* isomer lies 10.6 kJ mol^–1^ higher in energy (at ΔG^⧧^ = 78.0 kJ mol^–1^).

**Figure 5:**
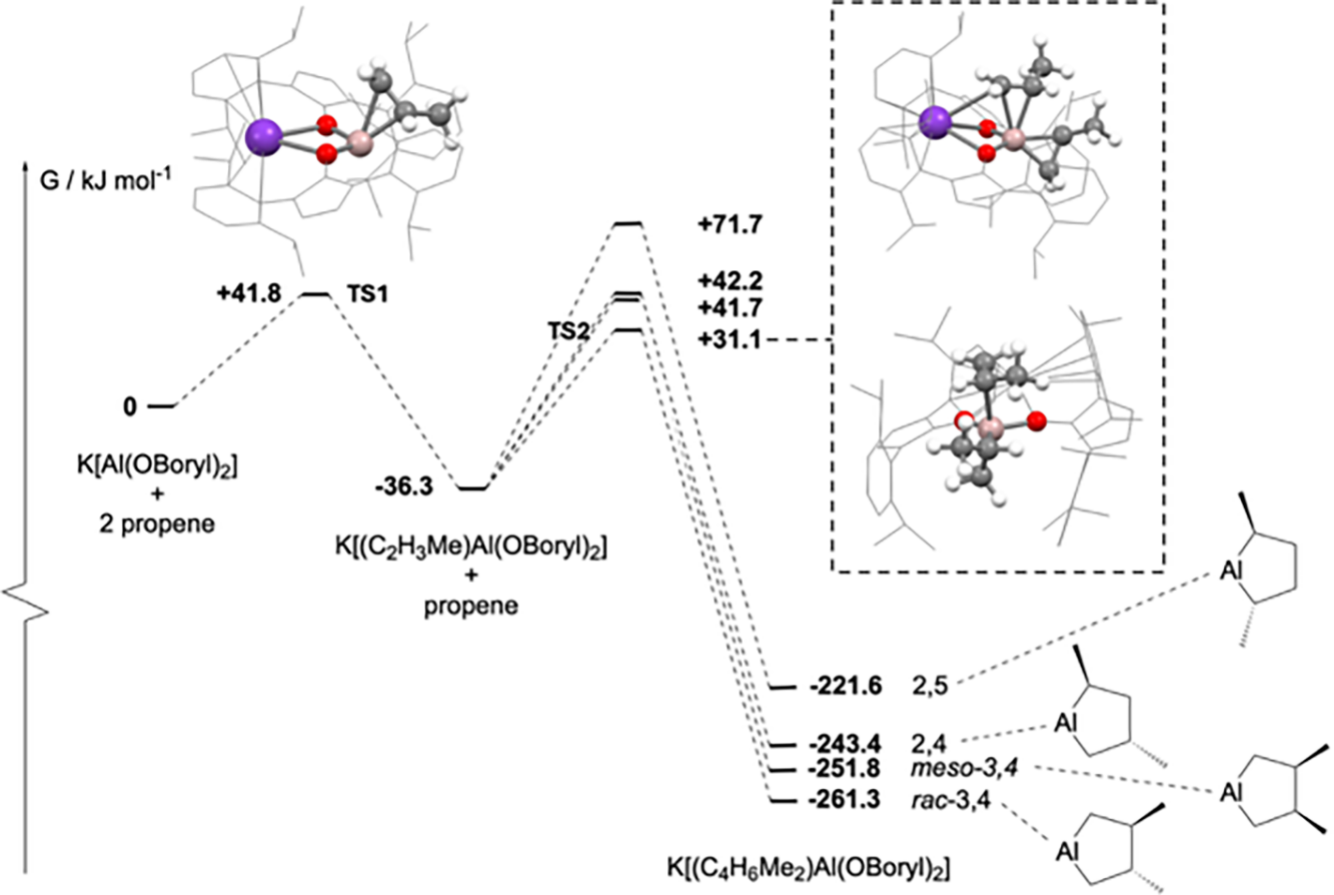
DFT-calculated mechanisms for the coupling of propene by **1**; (inset) product-determining transition state (two viewing angles).

**Figure 6:**
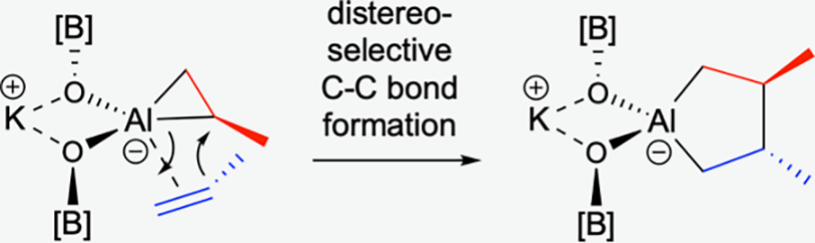
Steric origins of diastereoselectivity in the C–C coupling of propene.

Although the formation of **2** and **4** shows no evidence of physical reversibility, e.g. of alkene loss under vacuum, we sought to probe the possibility for *chemical* reversibility by examining their reactivity toward reagents known to trap either alumina-cyclopropanes or the parent aluminyl species itself (as might be formed by loss of one or both equivalents of alkene). With this in mind, we examined the reactivity of **2** and **4** toward CO, CO_2_ and internal alkynes.

Exposure of **2** to CO_2_ under very mild conditions (1 atm, ambient temperature) results in clean formation of the dimeric carboxylation product **5** (Scheme 2), which can be shown by SCXRD to feature a five-membered AlC_3_O heterocycle formed by formal substitution of one of the ethene moieties of **2** by the carbon and one oxygen atom from CO_2_. The solid-state structure of **5** features two such units linked by a pair of O- and arene-ligated K^+^ counterions (Figure S26). Chemical regeneration of ethene can also be demonstrated in the reaction of **2** with phenylacetylene (PhCCH). The release of ethene by C–C bond scission can be demonstrated explicitly by *in situ* ^1^H NMR monitoring (Figure S18), and in this case, X-ray crystallography shows that the aluminum-containing product is K­[(PhCC)­(Et)­Al­{OB­(NDippCH)_2_}_2_] (**6**) featuring one ethyl and one phenylacetylide ligand, suggesting that deprotonation of the PhCCH substrate enables the formation of the Et ligand from a C_2_H_4_ fragment. (Scheme 2). The significant steric demands of the phenyl substituents presumably prevent the formation of K­[(PhCC)_2_Al­{OB­(NDippCH)_2_}_2_]—despite use of excess PhCCH.

**Scheme 2:**
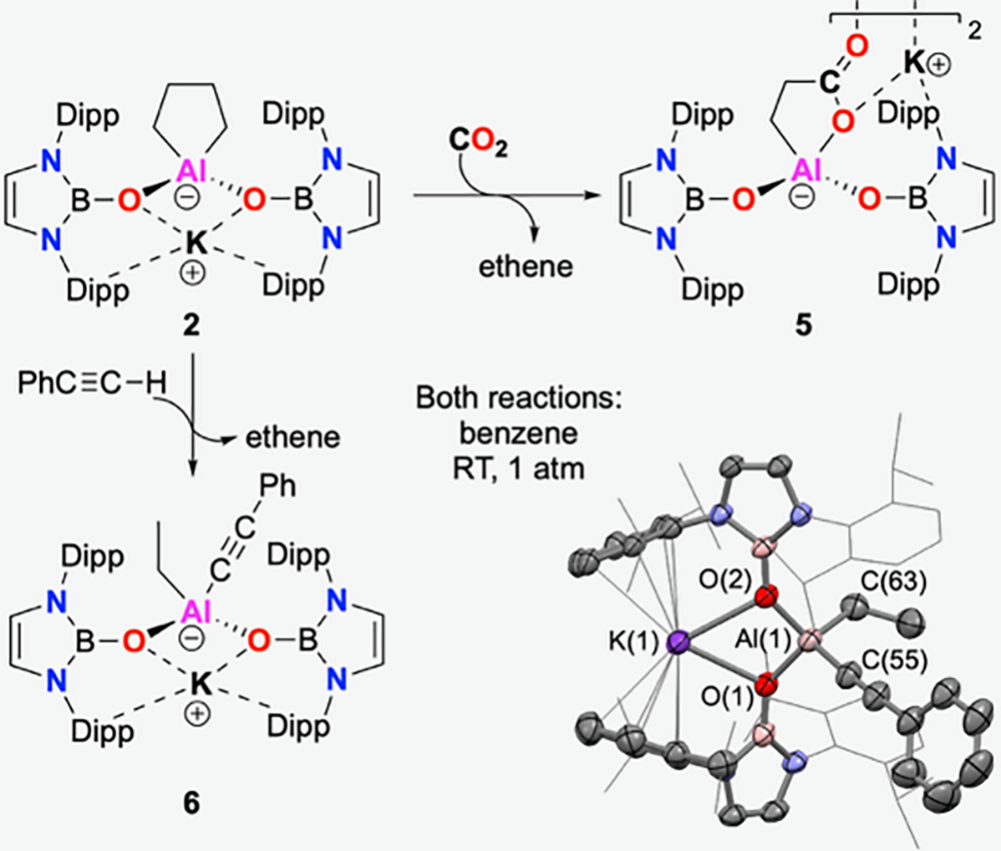
Reactions of **2** with CO_2_ and PhCCH Demonstrating Chemical Reversibility in C–C Bond Formation (with Accompanying Evolution of 1 or 2 equiv of Ethene)^a^

a Inset: molecular structure of compound **6** in the solid state as determined by X-ray crystallography. Thermal ellipsoids set at the 50% probability level; hydrogen atoms omitted, and ligand carbon atoms shown in wireframe format for clarity (see Figure S26 for the X-ray crystal structure of compound **5**).

Chemical reversibility involving C–C bond scission can also be demonstrated in the case of compound **4**, which undergoes clean carbonylation with CO to give **7** (plus propene) under similarly mild conditions, i.e. room temperature, 1 atm. pressure (Scheme 3). Formal substitution of one propene unit by CO is accompanied by an intra-annular 1,2-hydrogen shift from the β-position of the metallacycle (presumably via a keto–enol isomerization). Similar reactivity has been observed by Coles and co-workers for compound **II** (as its [18-crown-6] derivative) toward carbon monoxide.^18^ In this case, the CO molecule inserts into the Al–C bond of the alumina-cyclopropane, with the resulting four-membered ring isomerizing (with accompanying Al–C to Al–O conversion) concomitant with the 1,2-hydrogen shift.

**Scheme 3:**
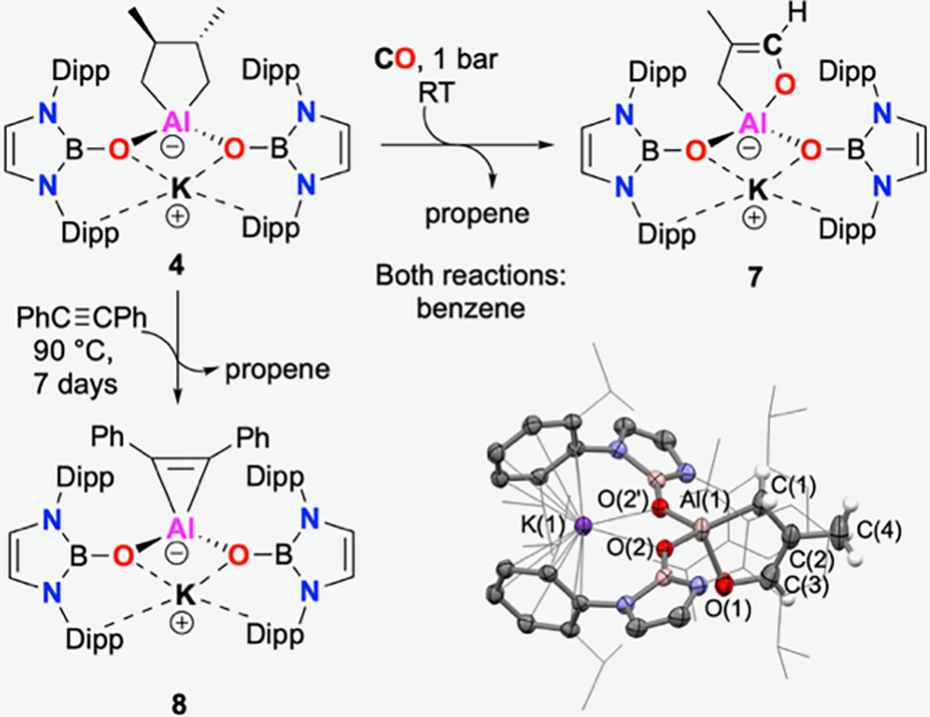
Reactions of **4** with CO and with PhCCPh^a^

a Inset: molecular structure of compound **7** in the solid state as determined by X-ray crystallography. Thermal ellipsoids set at the 50% probability level; selected hydrogen atoms and second disorder component omitted, and ligand carbon atoms shown in wireframe format for clarity (see Figure S27 for the X-ray crystal structure of compound **8**).

Chemical regeneration of propene is also evident in the reaction of **4** with diphenylacetylene, PhCCPh (albeit not selectively so). Heating **4** in the presence of PhCCPh at 90 °C leads to mixture of products, from which **8** can be crystallized in very low yield (ca. 5%). X-ray crystallography shows that **8** features a three-membered alumina-cyclopropene ring, implying that (for this product at least) *both* molecules of propene are released (Scheme 3).^50^

In summary, we have demonstrated the first structurally authenticated examples of single-site main-group-mediated reductive coupling of alkenes, exploiting an aluminyl compound supported by N-heterocyclic boryloxy ligands. In the case of propene, this chemistry occurs regio- and diastereoselectively to generate a *rac*-3,4-disubstituted alumina-cyclopentane. Isolation of the intermediate metalla-cyclopropane and DFT analysis allows the mechanism of these transformations to be elucidated, with coupling between the most hindered carbon centers in propene being driven by minimizing steric interactions with the bulky ligand scaffold. Although these transformations are physically irreversible (i.e. no “free” alkene is evolved under vacuum), chemical reversibility can be effected through the addition of small unsaturated substrates (CO, CO_2_, alkynes). In each of these reactions C–C bond scission occurs with accompanying loss of one or both alkene molecules; mechanistically, the very substantial activation barriers associated with spontaneous alkene loss from alumina-cyclopentane species (>250 kJ mol^–1^) suggests that these reactions (in contrast to related early d-block metal chemistry)^57–59^ occur via associative (or even concerted) mechanisms, with assimilation of the substrate facilitating alkene loss. As such, the transition state for the *associative* reaction of CO_2_ with metallacyclopentene **2** is calculated to be ca. 90 kJ mol^–1^ (cf. ca. 280 kJ mol^–1^ for ethene dissociation).

## Supplementary Material



## Notes

Complete synthetic and characterization data for new compounds, representative spectra, and details of quantum chemical calculations and optimized xyz-coordinates (PDF)

## Abbreviations

Dipp: 2,6-diisopropylphenyl
