# Supplementary material for: Diastereoselective and Chemically Reversible C–C Bond Formation Mediated by an (N-heterocyclic)boryloxy Aluminyl Compound
Source: J Am Chem Soc. 2026 May 20;148(21):21204–10. doi: 10.1021/jacs.6c03691 (PMC13244447; doi:10.1021/jacs.6c03691)
Supplement: Supplementary file 1 [file ja6c03691_si_001.pdf]

Diastereoselective and Chemically Reversible C-C Bond Formation Mediated by an (N-heterocyclic)boryloxy Aluminyl Compound

Authors: Debotra Sarkar, Petra Vasko, Job J. C. Struijs, Maximilian Dietz, and Simon Aldridge

[†] Inorganic Chemistry Laboratory, Department of Chemistry, University of Oxford, South Parks Road, Oxford, OX1 3QR (UK)

[‡] Department of Chemistry, Indian Institute of Technology Madras, Chennai 600036 (India)

[§] Department of Chemistry, University of Helsinki, A.I. Virtasen Aukio 1, P.O. Box 55, FI-00014 (Finland)

General Experimental Procedures S2
Syntheses, Spectroscopic Data and Representative Spectra for Novel Compounds S3
Single Crystal X-ray Diffraction Studies S19
Computational Details including Optimized xyz Coordinates S27
References S46

General Experimental Procedures

All manipulations were carried out using standard Schlenk line or dry-box techniques under an atmosphere of argon or dinitrogen. Solvents were degassed by sparging with argon and dried by passing through a column of the appropriate drying agent. Xylenes were refluxed over potassium, with the solvent then being distilled and stored under argon in Teflon valve ampoules. NMR spectra were measured in C_6D_6 (which was dried over potassium) or THF-d_8 (which was dried over $LiAlH_4$), with the solvent then being distilled under reduced pressure and stored under argon in Teflon valve ampoules. ^{1}H, $^{13}C\{^{1}H\}$ and $^{11}B\{^{1}H\}$ NMR spectra were recorded on Bruker 400 MHz spectrometer at ambient temperature and referenced internally to residual protio-solvent (^{1}H) or solvent (^{13}C) resonances and are reported relative to tetramethylsilane (δ = 0 ppm). Chemical shifts are quoted in δ (ppm) and coupling constants in Hz. Elemental analyses were carried out by London Metropolitan University. $[KAl\{OB(NDippCH)_2\}_2]$ was prepared by the literature method.[1]

Syntheses, Spectroscopic Data and Representative Spectra for Novel Compounds

K[(C_4H_8)Al{OB(NDippCH)$_2$}$_2$], 2

K[Al{OB(NDippCH)$_2$}$_2$] (**1**, 50 mg, 0.05 mmol) was dissolved in 0.5 mL of C_6D_6 and transferred to a J-Young NMR tube. The solution was freeze-pump-thaw degassed three times before being refilled with 1 bar of ethene. After 4 h, compound **2** started to crystallize inside the NMR tube (producing single crystals suitable for SC-XRD analysis). Compound **2** was separated from the mother solvent by decantation, then dried under reduced pressure, yielding a white crystalline powder. Yield: 44 mg, 0.04 mmol, 82%. Anal. Calcd. [%] for $C_{56}H_{80}AlB_2KN_4O_2$: C, 72.40; H, 8.68; N, 6.03; Found: 70.24; H, 7.92; N 5.65.

^{1}H NMR (400 MHz, THF-d$_8$, 297 K): δ = -1.47 (m, 4H, C*H*$_2$Al), 0.33 (m, 4H, C*H*$_2$CH$_2$Al), 0.98-1.29 (m, 48H, CH(C*H*$_3$)$_2$), 3.13-3.29 (m, 8H, C*H*(CH$_3$)$_2$), 5.72 (s, 4H, NC*H*), 7.00-7.16 (Ar^{Dipp}-*H*, 12H).

^{11}B{^{1}H} NMR (128 MHz, THF-d$_8$): δ = 19.8.

^{13}C{^{1}H} NMR (100 MHz, THF-d$_8$): δ = 14.59, 24.1 (CH(*C*H$_3$)$_2$), 28.5 (*C*H$_2$CH$_2$Al), 29.0-32.7 (*C*H(CH$_3$)$_2$), 116.8 (N*C*H), 123.9 (Ar-*C*), 126.9 (Ar-*C*), 142.0 (Ar-*C*), 147.9 (Ar-*C*).

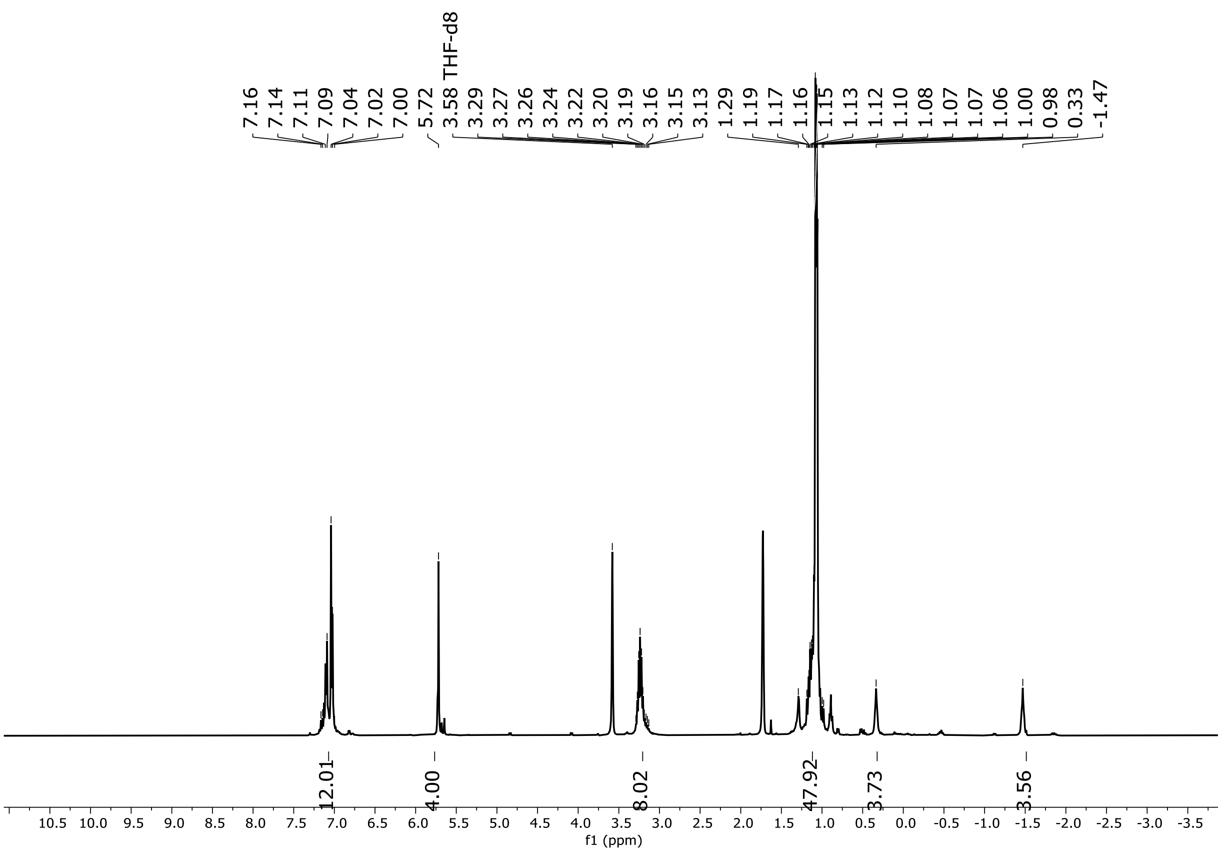

Figure S1. ^{1}H NMR spectrum of compound **2** in THF-d$_8$.

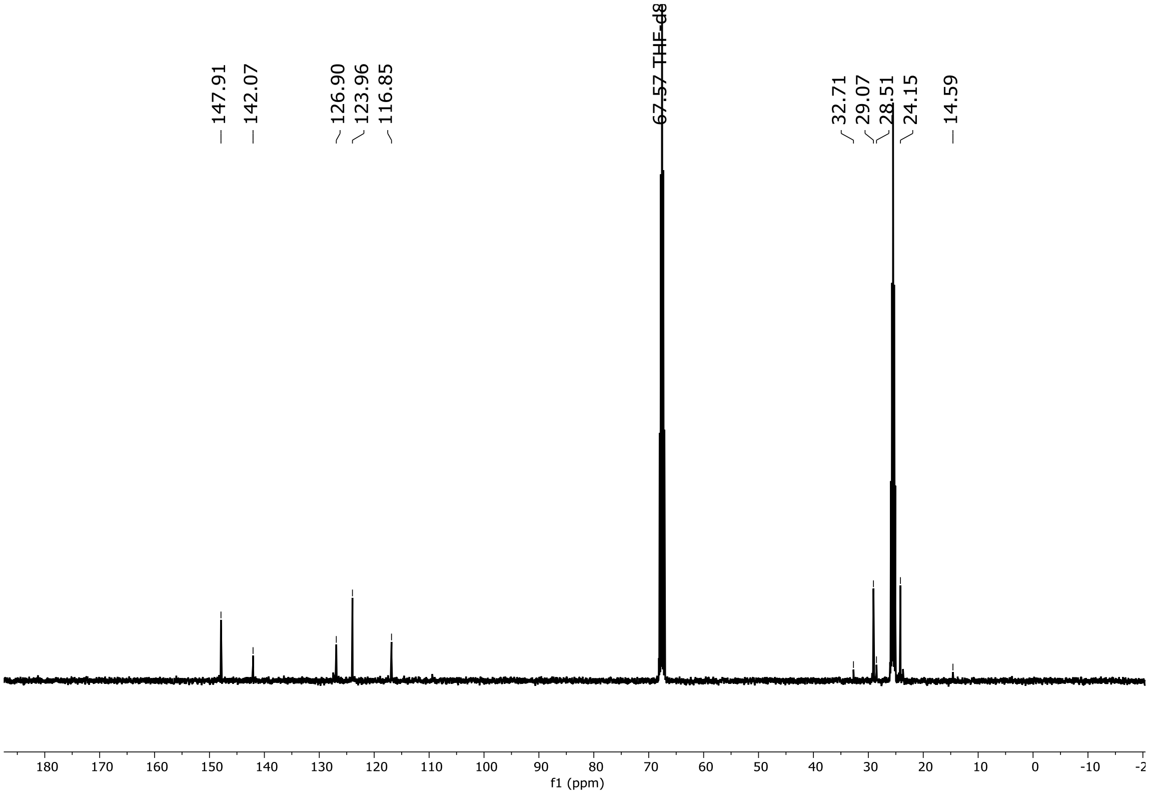

Figure S2. $^{13}C\{^{1}H\}$ NMR spectrum of compound **2** in THF-d_8.

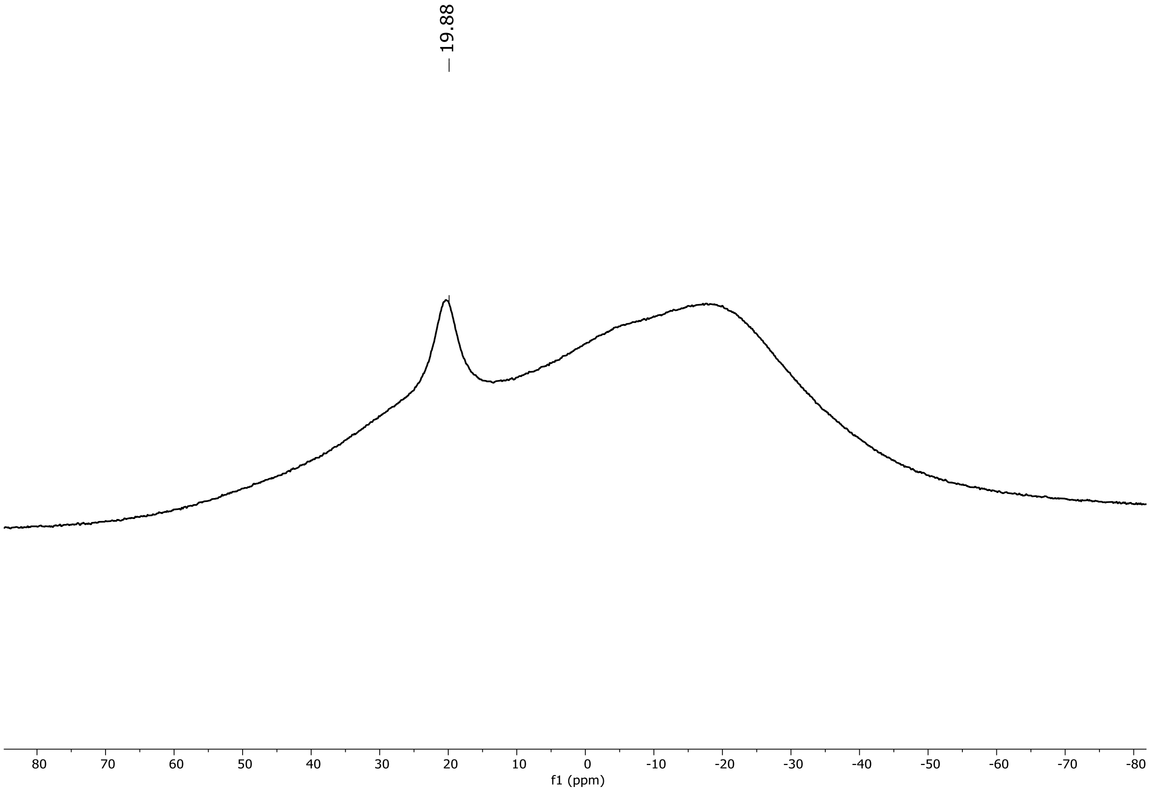

Figure S3. $^{11}B\{^{1}H\}$ NMR spectrum of compound **2** in THF-d_8.

[K(2.2.2-crypt)][(C_4H_8)Al{OB(NDippCH)$_2$}$_2$], 2'

[K(2.2.2-crypt)][(HCDippN)$_2$BO]$_2$Al] (**1'**, 50 mg, 0.04 mmol) was dissolved in 0.6 mL of THF-d$_8$ and transferred to a J-Young NMR tube. The solution was freeze-pump-thaw degassed three times before being refilled with 1 bar of ethene. After 4 h, the solution was filtered, the solution concentrated to 0.3 mL and hexane (0.5 mL) added to aid crystallization. After 7 d, colourless crystals of compound **2'** were obtained, which were suitable for X-ray diffraction. For further analysis, the solvent was decanted, and the colourless crystals were washed once with hexane and dried under high vacuum (17 mg, 0.01 mmol, 32% yield). Anal. Calcd. [%] for $C_{74}H_{116}AlB_2KN_6O_8$: C, 68.08; H, 8.96; N, 6.44; Found: 67.12; H, 8.25; N 5.38.

^{1}H NMR (400 MHz, THF-d$_8$, 297 K): δ = -1.61 (m, 4H, C*H*$_2$Al), 0.59 (m, 4H, C*H*$_2$CH$_2$Al), 0.95-1.04 (m, 48H, CH(C*H*$_3$)$_2$), 2.55 (m,12H,NCH2-crypt.), 3.22-3.32 (m, 8H, C*H*(CH$_3$)$_2$), 3.54–3.56 [br, 24H, {(12H, NCH2CH2crypt)+ (12H, OCH2-crypt)}], 5.57 (s, 4H, NC*H*), 6.89-6.99 (Ar^{Dipp}-*H*, 12H).

^{11}B{^{1}H} NMR (128 MHz, THF-d$_8$): δ = 19.4.

^{13}C{^{1}H} NMR (100 MHz, THF-d$_8$): δ = 13.4 (CH$_2$*C*H$_2$Al), 24.3 (CH(*C*H$_3$)$_2$), 28.8 (*C*H$_2$CH$_2$Al), 29.8 (*C*H(CH$_3$)$_2$), 54.8 (N*C*H$_2$-crypt.), 57.7 (NCH$_2$*C*H$_2$-crypt.), 68.6 (NCH$_2$*C*H$_2$-crypt.), 71.0-71.8 (O*C*H$_2$-crypt.), 116.2 (N*C*H), 123.0 (Ar-*C*), 125.5 (Ar-*C*), 142.9 (Ar-*C*), 147.4 (Ar-*C*).

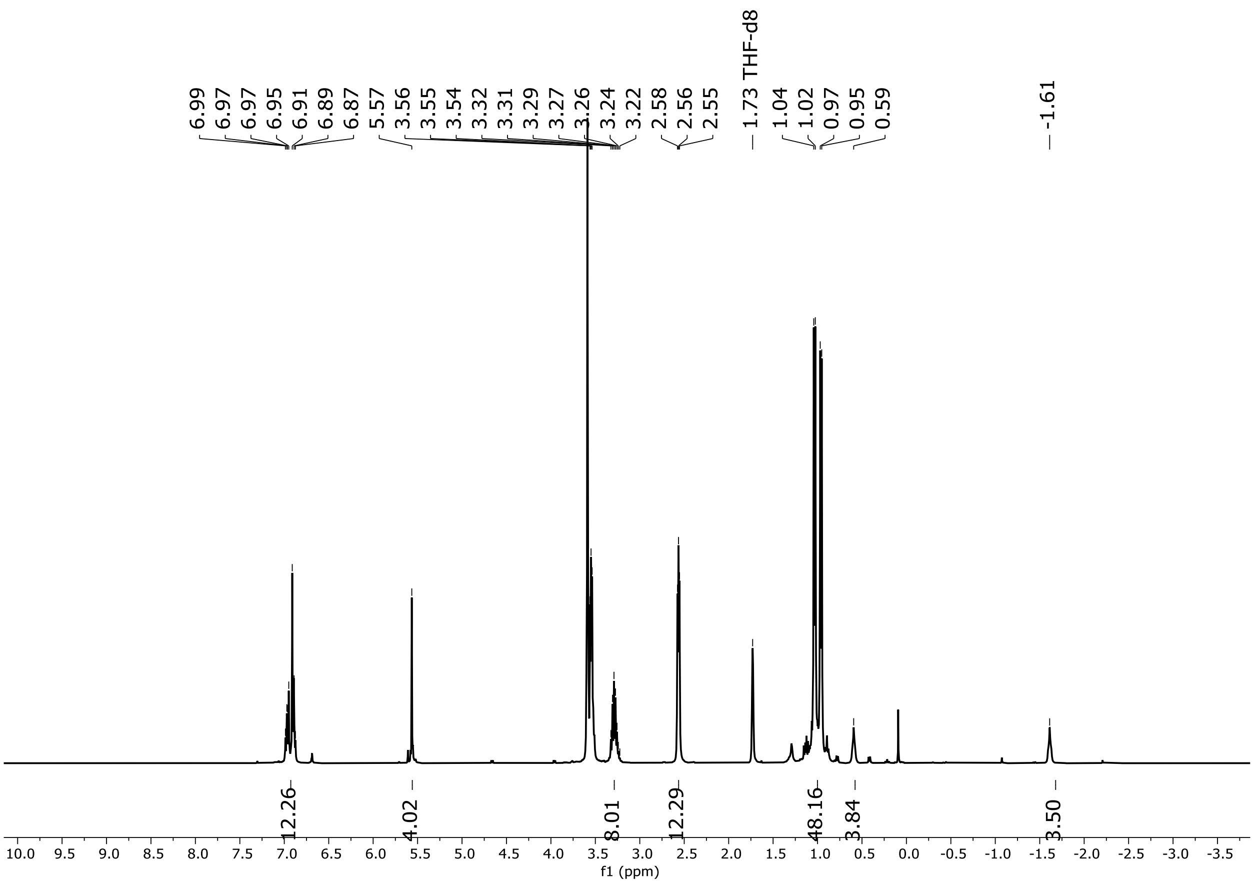

Figure S4. ^{1}H NMR spectrum of compound **2'** in THF-d$_8$.

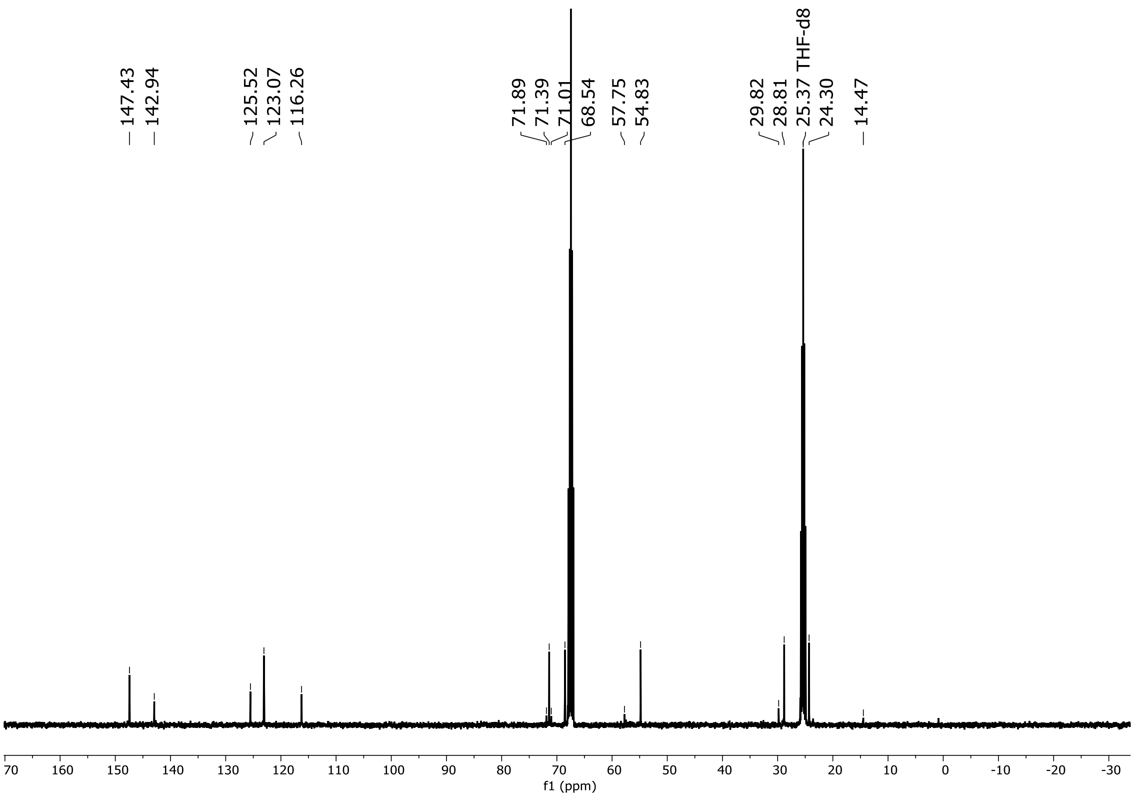

Figure S5. $^{13}C\{^{1}H\}$ NMR spectrum of compound **2'** in THF-d_8.

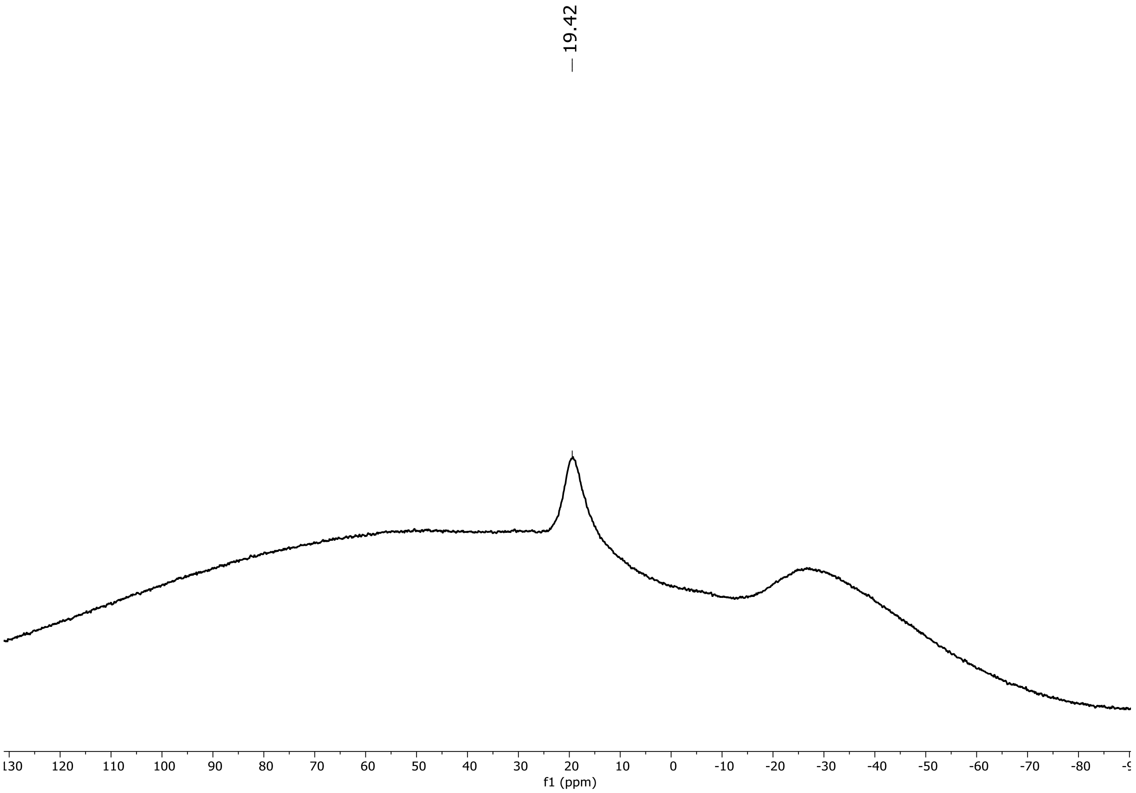

Figure S6. $^{11}B\{^{1}H\}$ NMR spectrum of compound **2'.**

Identification of K[($C_2H_3Me_2$-3,4)Al{OB(NDippCH)$_2$}$_2$], 3

K[Al{OB(NDippCH)$_2$}$_2$] (**1**, 70 mg, 0.08 mmol) was dissolved in 0.5 mL of C_6D_6 and transferred to a J-Young NMR tube. The solution was freeze-pump-thaw degassed three times before being refilled with 1 bar of propene. Immediately acquired in situ ^{1}H and ^{11}B NMR suggested the formation of an intermediate species prior to **4**. For crystallization, the NMR tube was degassed and quickly transferred into a glovebox. The solution was filtered, and slow evaporation of the solvent at room temperature led to the formation of a mixture of colourless crystals containing compound **3** (minor component; yield ≈ 3 %) and compound **4** (major component; yield ≈ 97 %), suitable for SC-XRD analysis.

^{1}H NMR (400 MHz, C_6D_6, 297 K): δ = -0.80-0.59 (m, 2H, CHC*H*$_2$Al), 0.03 (m, 1H, C*H*CH$_2$Al), 1.14-1.27 [m, 51H, {48H, CH(C*H*$_3$)$_2$)+ 3H (C*H*$_3$CHCH$_2$Al)}] , 3.32-3.42 (m, 8H, C*H*(CH$_3$)$_2$), 5.92 (s, 4H, NC*H*), 7.01-7.21 (12H, Ar^{Dipp}-*H*).

^{11}B{^{1}H} NMR (128 MHz, C_6D_6): δ = 20.0.

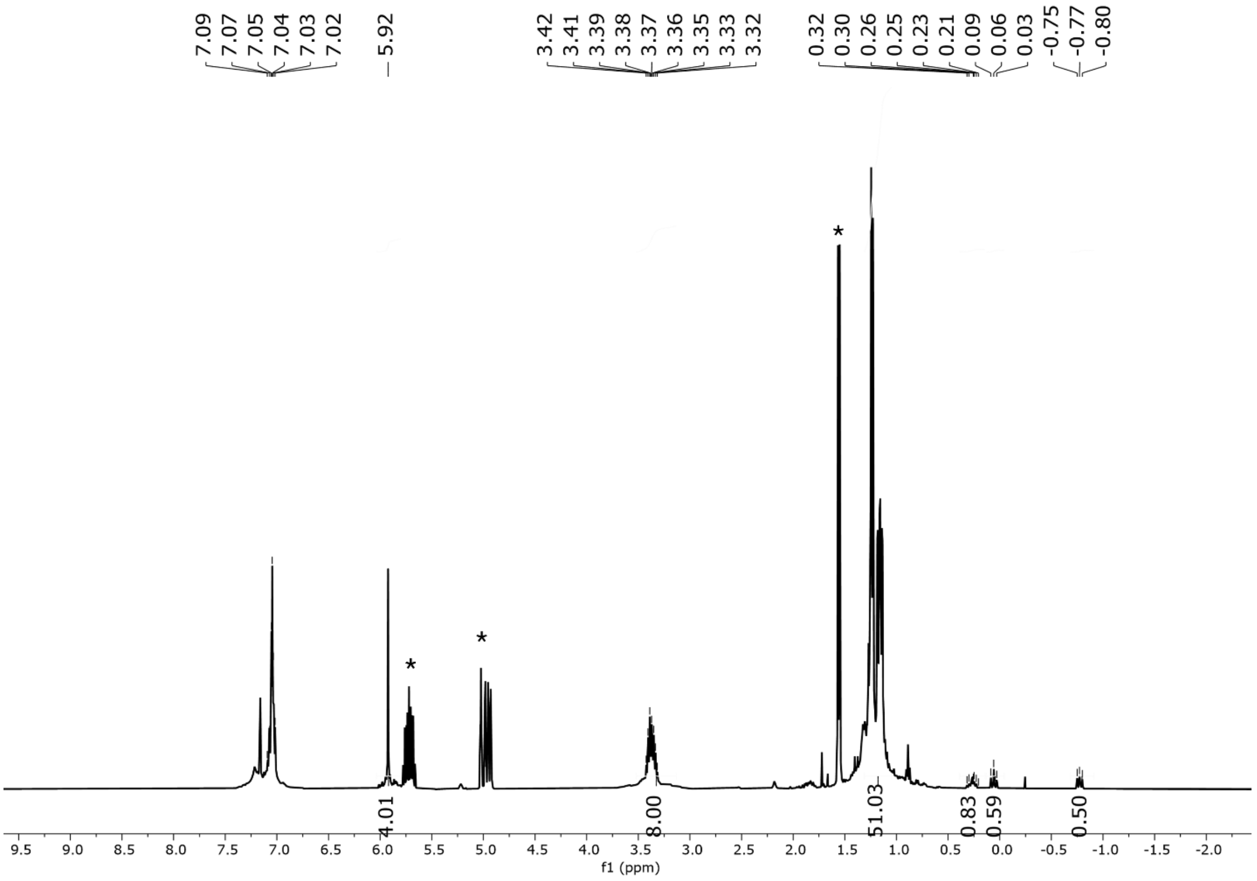

Figure S7. ^{1}H NMR spectrum of compound **3** in C_6D_6.

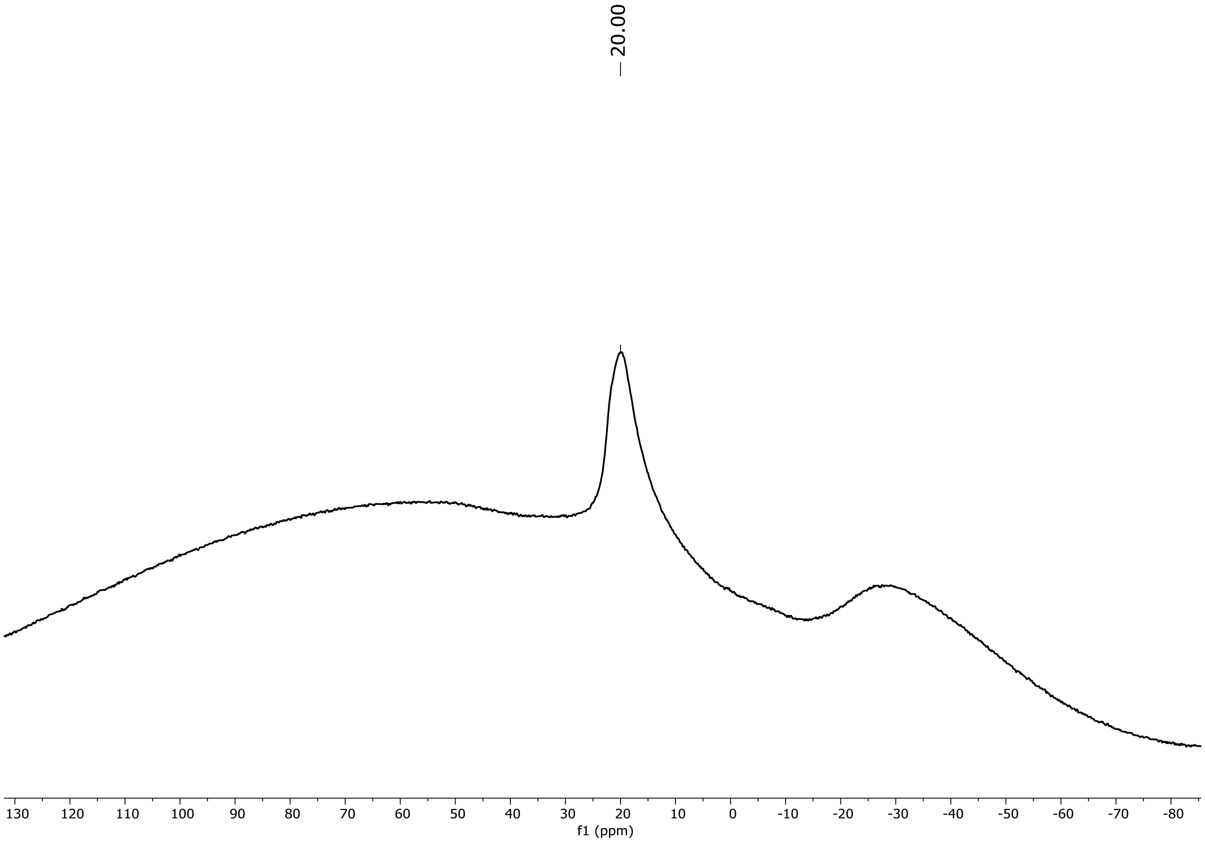

Figure S8. $^{11}B\{^{1}H\}$ NMR spectrum of compound **3** in C_6D_6.

K[($C_4H_6Me_2$-3,4)Al{OB(NDippCH)$_2$}$_2$], 4

K[Al{OB(NDippCH)$_2$}$_2$] (**1**, 70 mg, 0.08 mmol) was dissolved in 0.5 mL of C_6D_6 and transferred to a J-Young NMR tube. The solution was freeze-pump-thaw degassed three times before being refilled with 1.2 bar of propene. After 15 min, compound **4** started to crystallize inside the NMR tube (producing single crystals suitable for SC-XRD analysis). Compound **4** was separated from the mother solvent by decantation, washed with pentane and dried under reduced pressure, yielding compound a white crystalline powder (55 mg, 0.05 mmol, 72%). Anal. Calcd. [%] for $C_{58}H_{84}AlB_2KN_4O_2$: C, 72.79; H, 8.85; N, 5.85; Found: C, 71.26; H, 8.19; N, 4.72.

^{1}H NMR (400 MHz, C_6D_5Br, 297 K): δ = -1.20-0.25 (m, 4H, C*H*$_2$Al), 0.30-0.92 (m, 2H, C*H*CH$_2$Al), 0.81-0.82 (m, 3H, C*H*$_3$CHCH$_2$Al), 1.14-1.27 [m, 51H, {48H, CH(C*H*$_3$)$_2$)+ 3H (C*H*$_3$CHCH$_2$Al)}] , 3.27-3.42 (m, 8H, C*H*(CH$_3$)$_2$), 6.01 (s, 4H, NC*H*), 7.01-7.36 (12H, Ar^{Dipp}-*H*).

^{11}B{^{1}H} NMR (128 MHz, C_6D_5Br): δ = 20.6.

^{13}C{^{1}H} NMR (100 MHz, C_6D_5Br): δ = 14.6 (CH*C*H$_2$Al), 23.2-25.3 (*C*H$_3$), 28.3-28.4 (*C*HCH$_2$Al), 31.9 (*C*H(CH$_3$)$_2$), 116.4 (N*C*H), 123.3-127.7 (Ar-*C*), 140.0 (Ar-*C*), 147.2 (Ar-*C*), 147.4 (Ar-*C*), 147.7 (Ar-*C*).

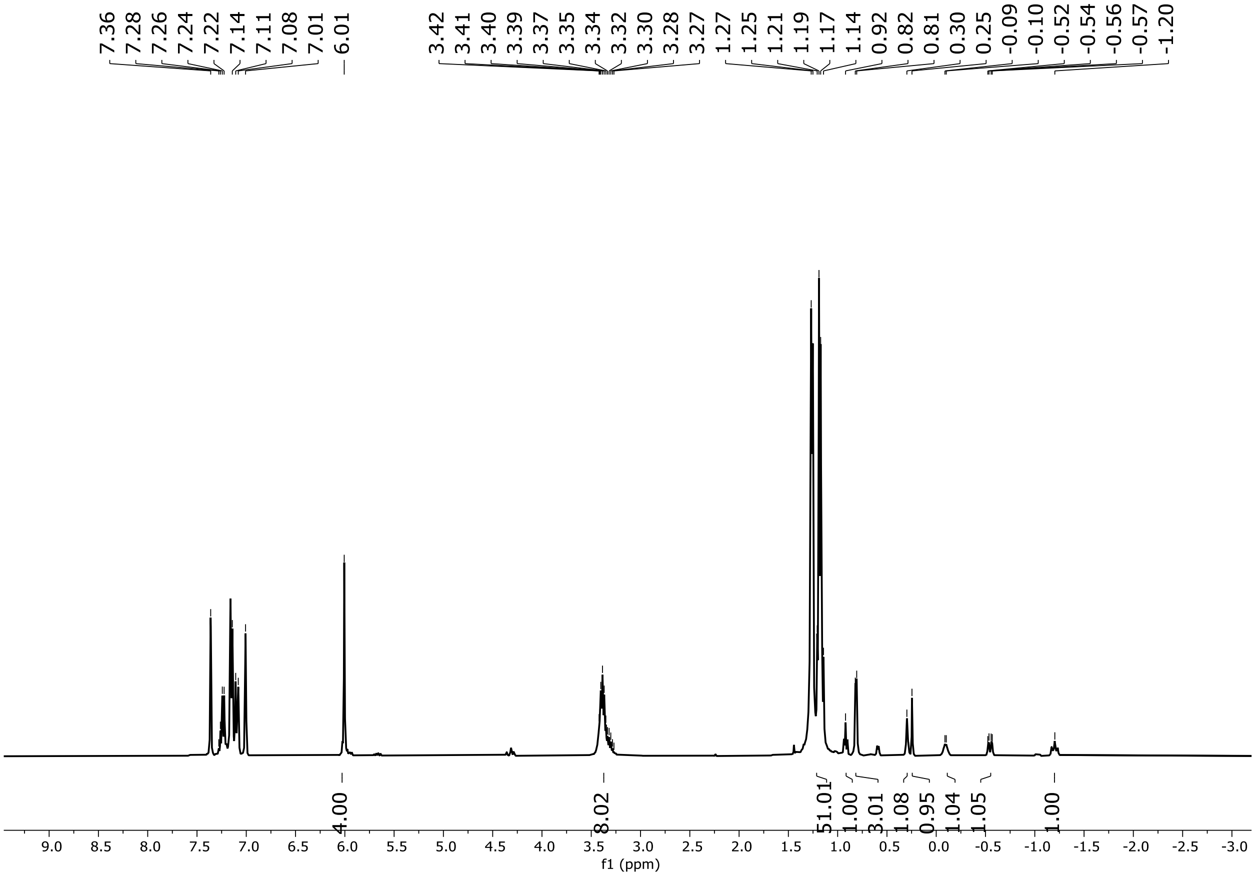

Figure S9. ^{1}H NMR spectrum of compound **4** in C_6D_5Br.

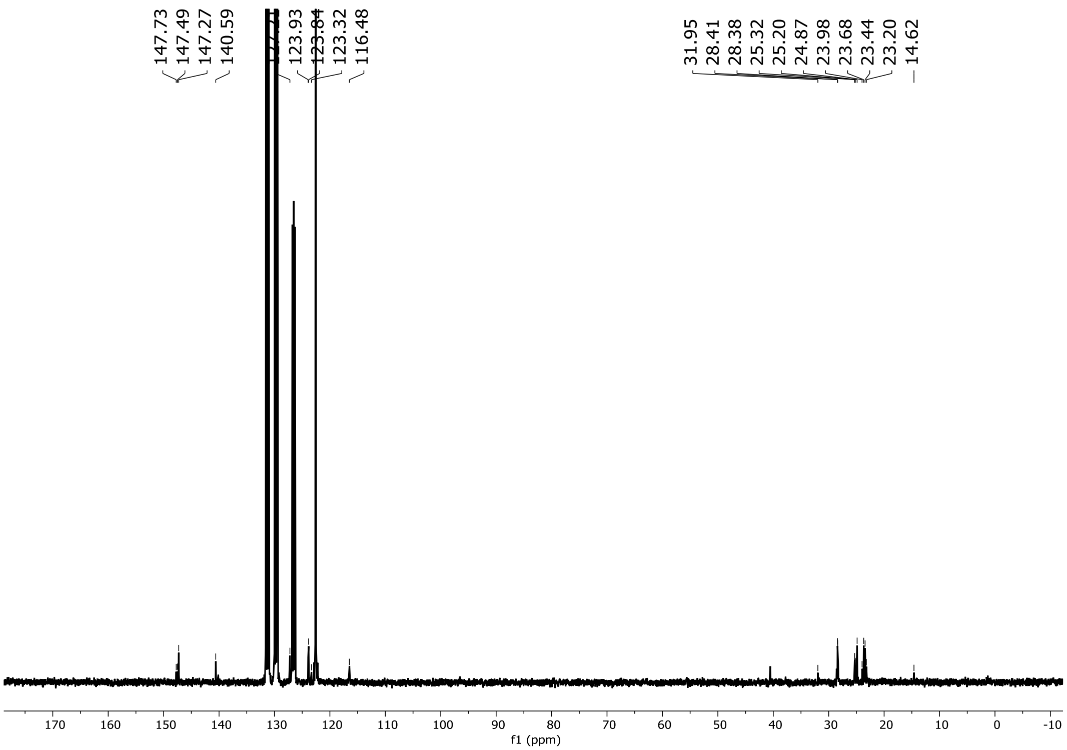

Figure S10. $^{13}C\{^{1}H\}$ NMR spectrum of compound **4** in C_6D_5Br.

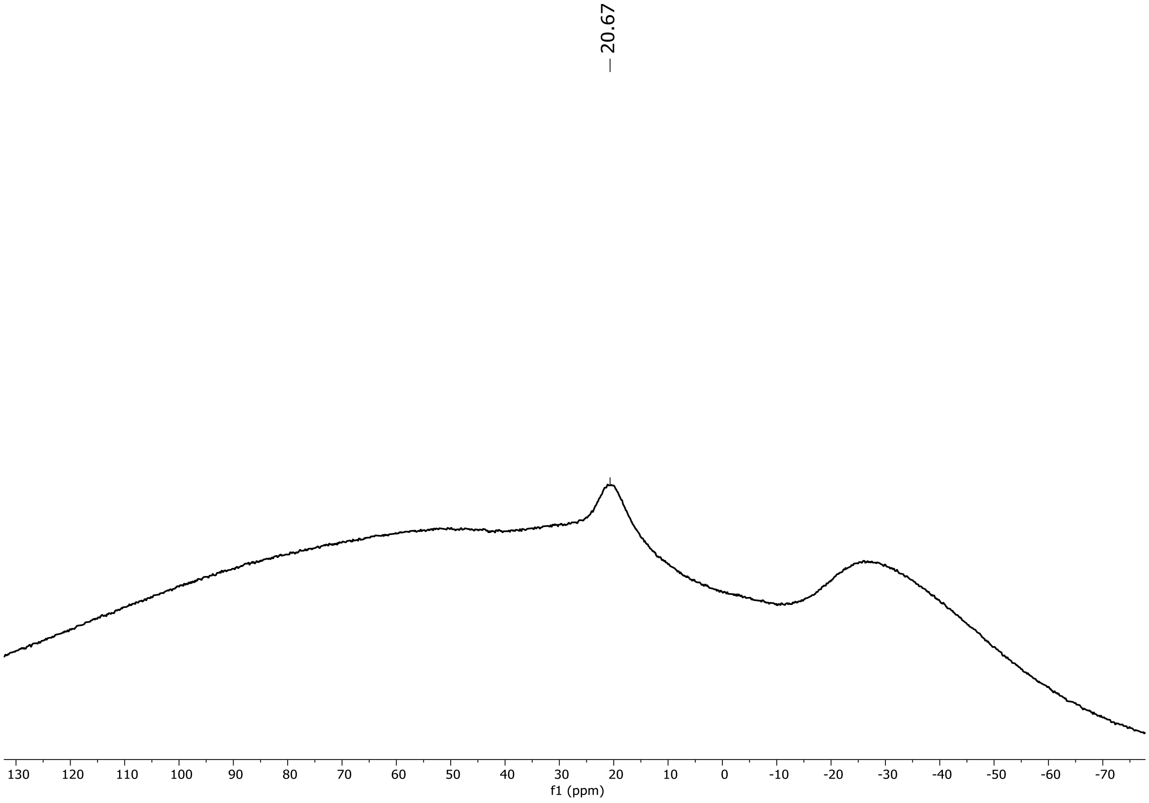

Figure S11. $^{11}B\{^{1}H\}$ NMR spectrum of compound **4** in C_6D_5Br.

K[(C_3H_4O)Al{OB(NDippCH)$_2$}$_2$], 5

Compound **2** (50 mg, 0.05 mmol) was dissolved in 0.5 mL of C_6D_6 and transferred to a J-Young NMR tube. The solution was freeze-pump-thaw degassed three times before being refilled with 1 bar of CO_2. *In situ* ^{1}H NMR suggested the immediate conversion of a new compound. The solution was filtered, and all volatiles were removed under reduced pressure, yielding compound **5** as a spectroscopically pure white powder (44 mg, 0.04 mmol, 86 %) Single crystals suitable for X-ray diffraction analysis were obtained via slow evaporation of a benzene solution of **5** (in a glove box), at room temperature. Anal. Calcd. [%] for $C_{52}H_{72}AlB_2KN_4O_2$: C, 69.91; H, 8.11; N, 5.93; Found: C, 68.51; H, 7.32; N, 4.25.

^{1}H NMR (400 MHz, THF-d$_8$, 297 K): δ = -1.43 (m, 2H, CH$_2$C*H*$_2$Al), 0.91-1.08 [m, 48H, CH(C*H*$_3$)$_2$], 1.28-1.32 (m, 2H, C*H*$_2$CH$_2$Al), 3.11-3.21 (m, 8H, C*H*(CH$_3$)$_2$), 5.64 (s, 4H, NC*H*), 6.96-7.09 (12H, Ar^{Dipp}-*H*).

^{13}C{^{1}H} NMR (100 MHz, THF-d$_8$): δ = 24.2-24.6 (CH(*C*H$_3$)$_2$), 28.9 (*C*HCH$_2$Al), 34.2 (*C*H(CH$_3$)$_2$), 116.5 (N*C*H), 123.3 (Ar-*C*), 123.4 (Ar-*C*), 126.3 (Ar-*C*), 141.8 (Ar-*C*), 147.5 (Ar-*C*), 147.6 (Ar-*C*), 184.9 (*C*(O)OAl).

^{11}B{^{1}H} NMR (128 MHz, THF-d$_8$): δ = 19.3.

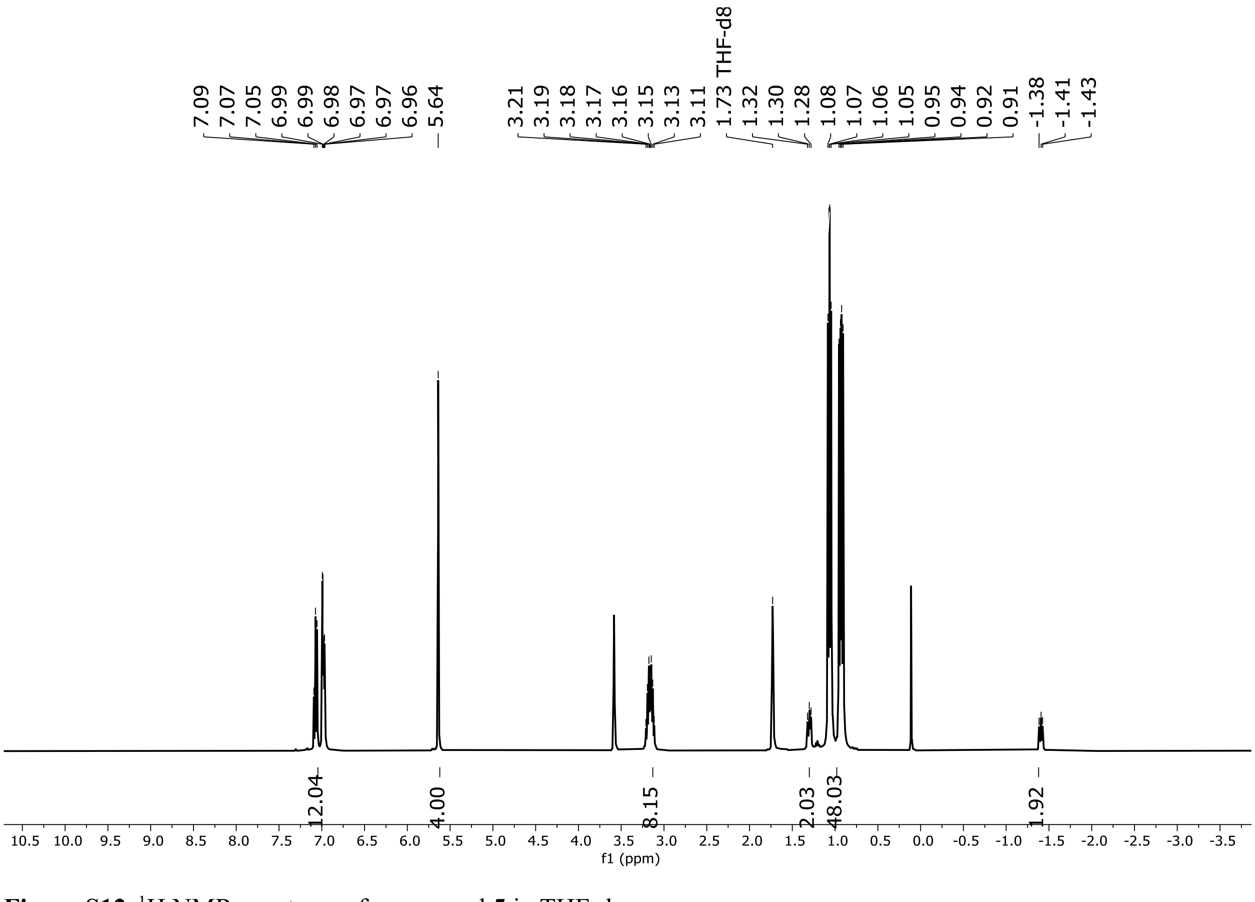

Figure S12. ^{1}H NMR spectrum of compound **5** in THF-d$_8$.

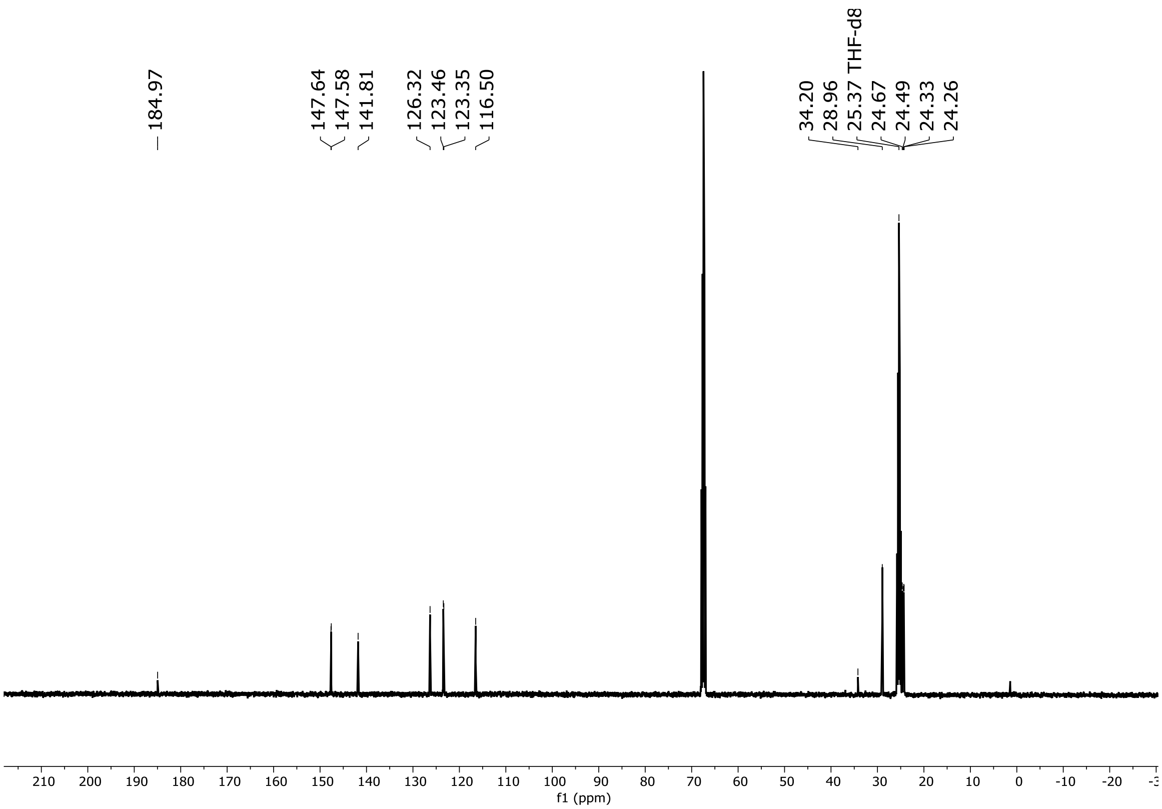

Figure S13. $^{13}C\{^1H\}$ NMR spectrum of compound **5** in THF-d_8.

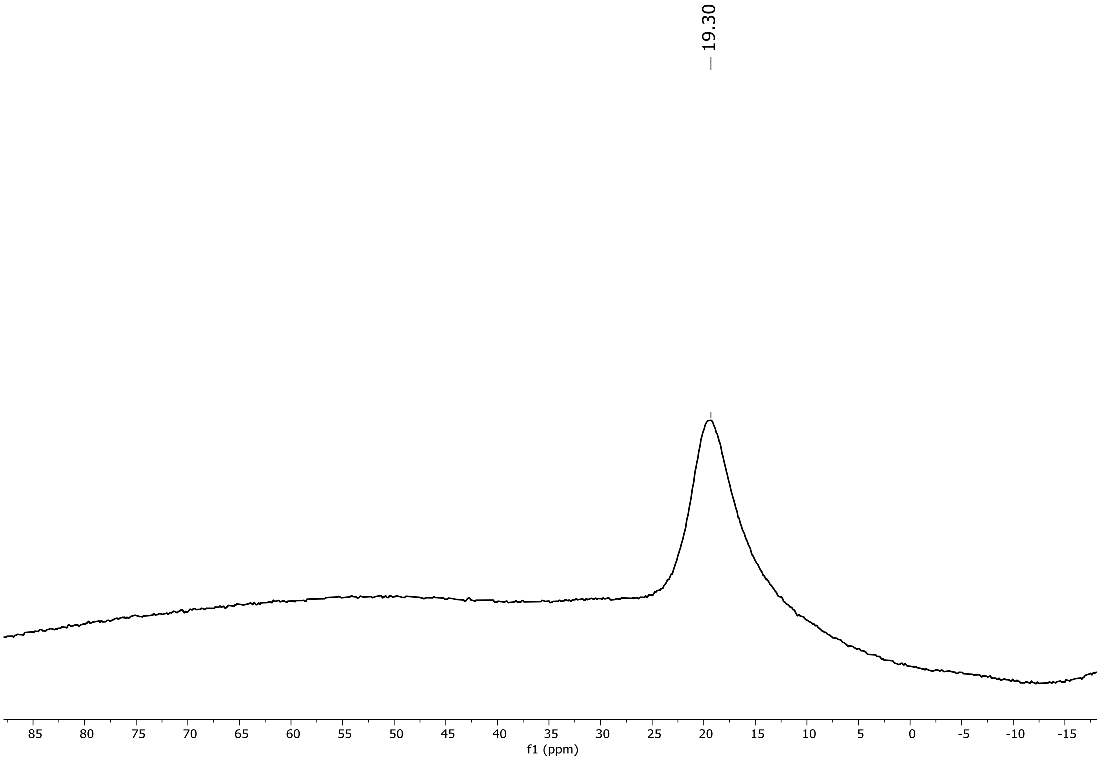

Figure S14. $^{11}B\{^1H\}$ NMR spectrum of compound **5** in THF-d_8.

K[{(Et)(CCPh)}Al{OB(NDippCH)$_2$}$_2$], 6

To a mixture of **2** (70 mg, 0.07 mmol) and PhCCH (7.7 mg, 0.07 mmol) was added benzene (3 mL), and the reaction mixture stirred for 2 h at room temperature. The solution was filtered, and all volatiles were removed under reduced pressure, yielding compound **6** as an off-white powder. Single crystals suitable for X-ray diffraction analysis were obtained via slow evaporation of a hexane/benzene mixture (0.5mL+0.2mL) in a glove box, at room temperature. Yield: 56 mg, 0.05 mmol, 74%.

^{1}H NMR (400 MHz, THF-d$_8$, 297 K): δ = -1.75 (m, 2H, C*H*$_2$Al), 0.17 (m, 2H, C*H*$_3$CH$_2$Al), 1.07-1.13 (m, 48H, CH(C*H*$_3$)$_2$), 3.24-3.34 (m, 8H, C*H*(CH$_3$)$_2$), 5.74 (s, 4H, NC*H*), 7.03-7.16 (Ar^{Dipp}-*H*, 12H).

^{11}B{^{1}H} NMR (128 MHz, THF-d$_8$): δ = 20.7.

^{13}C{^{1}H} NMR (100 MHz, THF-d$_8$): δ = 9.7 (*C*H$_3$CH$_2$Al), 14.4 (*C*H$_3$CH$_2$Al), 23.5-24.4 (CH(*C*H$_3$)$_2$), 29.0-32.5 (*C*H(CH$_3$)$_2$), 116.9 (N*C*H), 123.8 (C*C*Ph), 124.0 (Ar-*C*), 126.04 (Ar-*C*), 127.0 (Ar-*C*), 128.0 (Ar-*C*), 132.4 (Ar-*C*), 141.7 (Ar-*C*), 148.0(Ar-*C*).

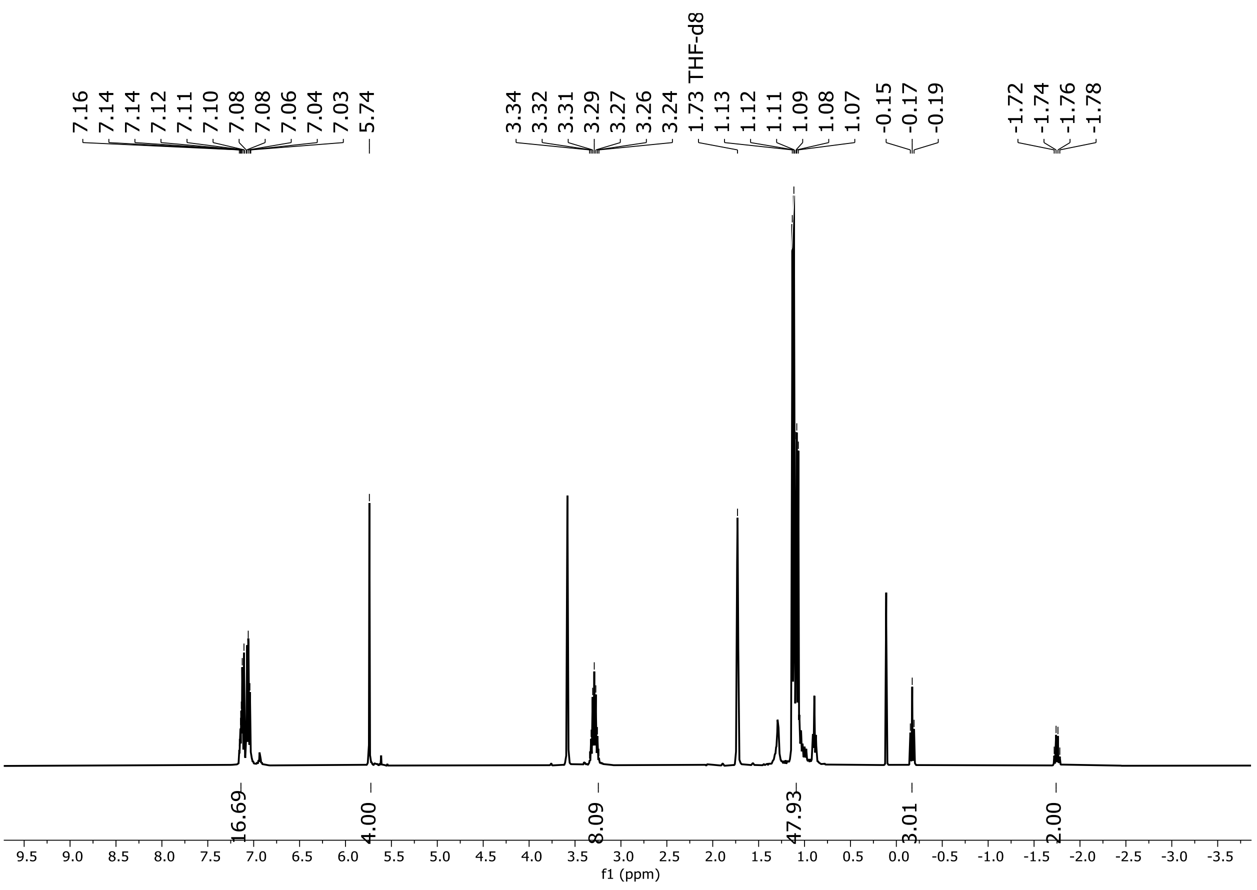

Figure S15. ^{1}H NMR spectrum of compound **6** in THF-d$_8$.

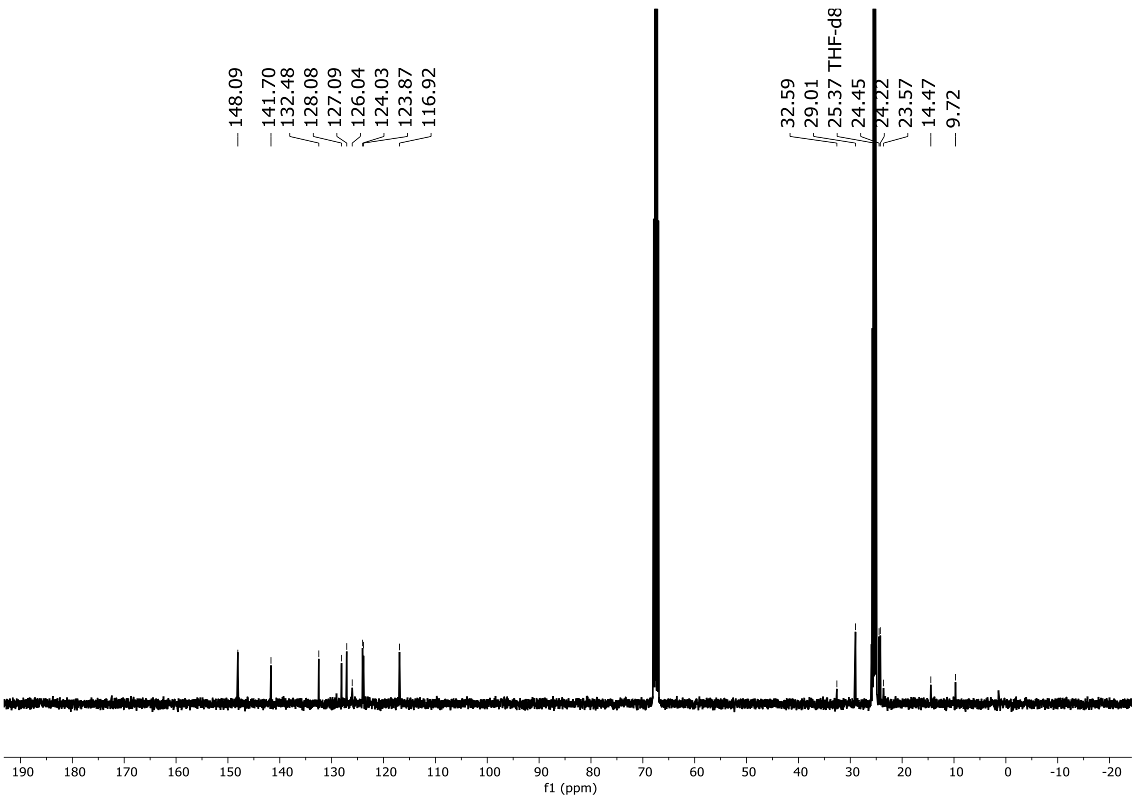

Figure S16. $^{13}C\{^{1}H\}$ NMR spectrum of compound **6** in THF-d_8.

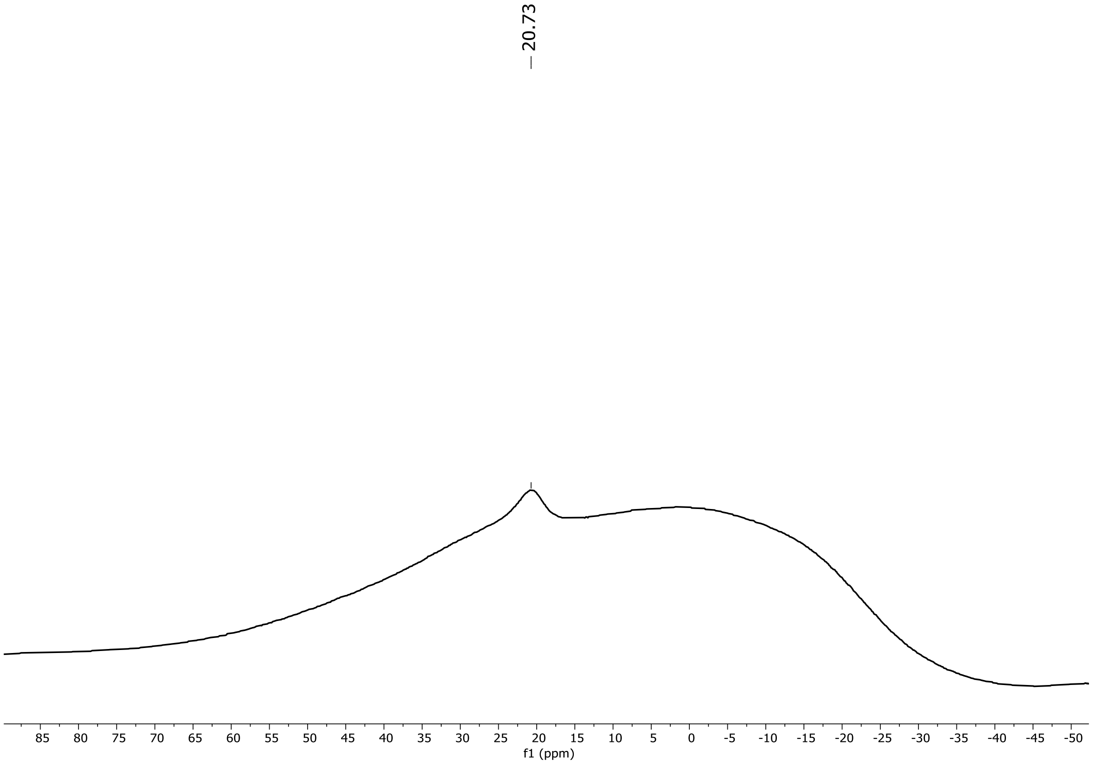

Figure S17. $^{11}B\{^{1}H\}$ NMR spectrum of compound **6** in THF-d_8.

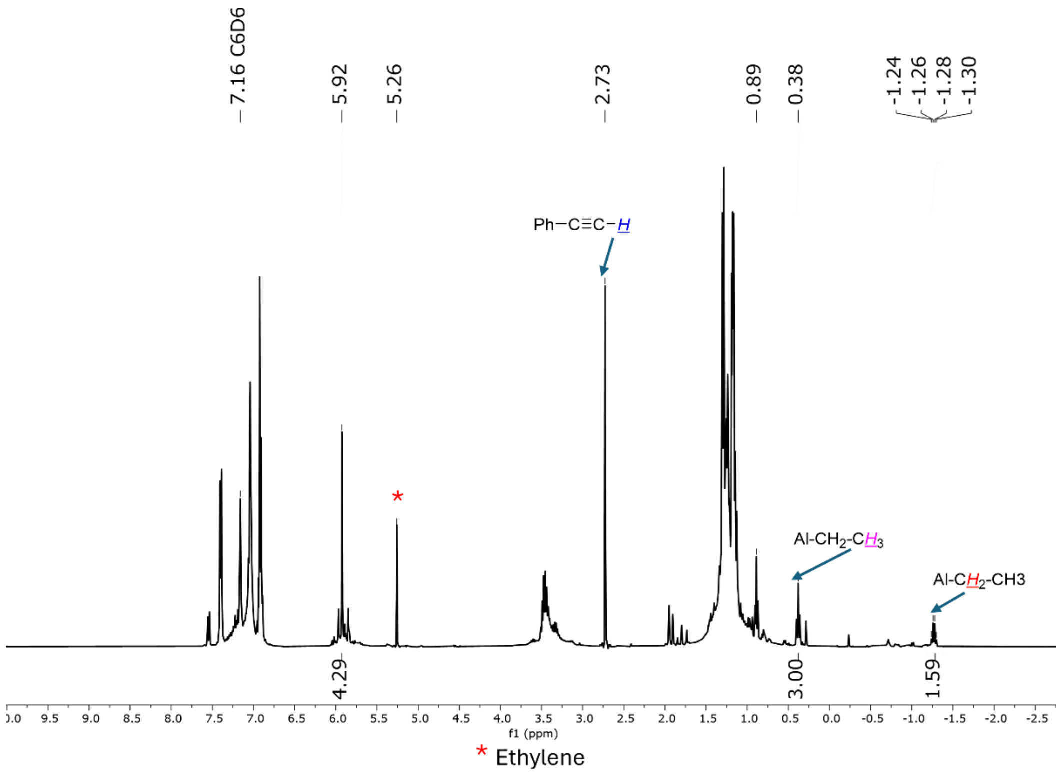

Figure S18. Crude ^{1}H NMR spectra of the reaction between Compound 2 and excess PhCCH.

K[(C_3H_4O)Al{OB(NDippCH)$_2$}$_2$], 7

Compound **4** (50 mg, 0.04 mmol) was dissolved in 0.5 mL of C_6D_6 and transferred to a J-Young NMR tube. The solution was freeze-pump-thaw degassed three times before being refilled with 1 bar of CO. *In situ* ^{1}H NMR measurements suggested the immediate formation of a new compound. The solution was filtered and all volatiles removed under reduced pressure, yielding compound **7**, as a spectroscopically pure powder in 69% yield. Single crystals suitable for X-ray diffraction analysis were obtained via slow evaporation of a benzene solution in a glove box at room temperature.

^{1}H NMR (400 MHz, THF-d$_8$, 297 K): δ = 0.38-0.48 (m, 3H, Ar-C*H*$_3$), 0.7-1.17 [m, 50H, {(45H, CH(C*H*$_3$)$_2$) + (2H, C*H*$_2$Al) + (3H, (O)HC=C(C*H*$_3$)C*H*$_2$Al)], 2.74-3.44 (m, 8H, C*H*(CH$_3$)$_2$), 5.37-5.56 (s, 4H, NC*H*), 6.87-7.24 [m, 13H, {(Ar^{Dipp}-*H*, 12H)+(1H, (O)C*H*)}].

^{11}B{^{1}H} NMR (128 MHz, THF-d$_8$): δ = 20.4.

^{13}C{^{1}H} NMR (100 MHz, THF-d$_8$): δ = 14.4 (*C*H$_2$Al), 22.2-27.5 (CH(*C*H$_3$)$_2$), 28.0-32.5 (*C*H(CH$_3$)$_2$), 116.7-119.7 (N*C*H), 123.4 (Ar-*C*), 124.3 (Ar-*C*), 124.5 (Ar-*C*), 125.0 (Ar-*C*), 125.9 (Ar-*C*), 126.0 (Ar-*C*), 126.7 (Ar-*C*), 128.4 (Ar-*C*), 140.4, 142.2, 142.4 (Ar-*C*), 146.3 (Ar-*C*), 147.2 (Ar-*C*), 147.8 (Ar-*C*), 148.0 (Ar-*C*), 148.2 (Ar-*C*), 149.7 (Ar-*C*).

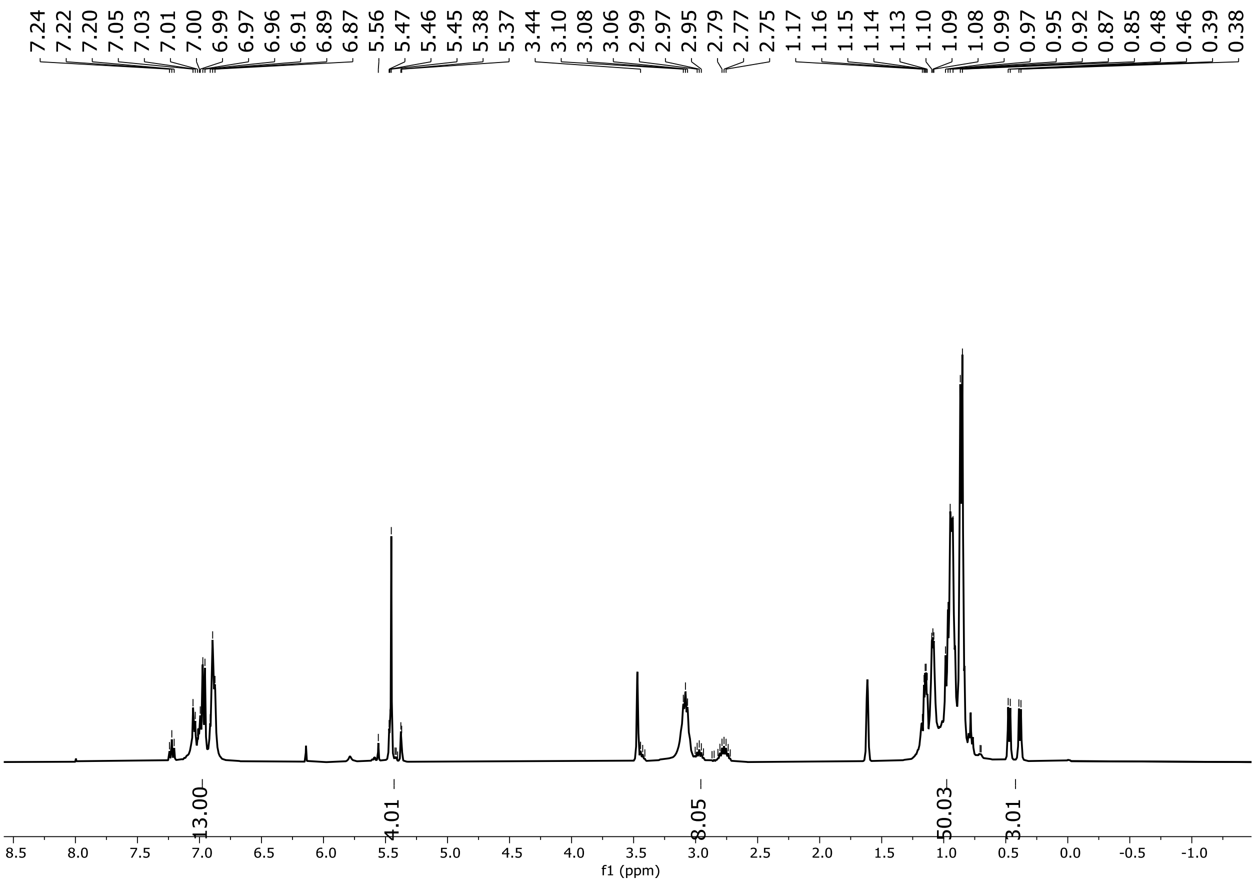

Figure S19. ^{1}H NMR spectrum of compound **7** in THF-d_8.

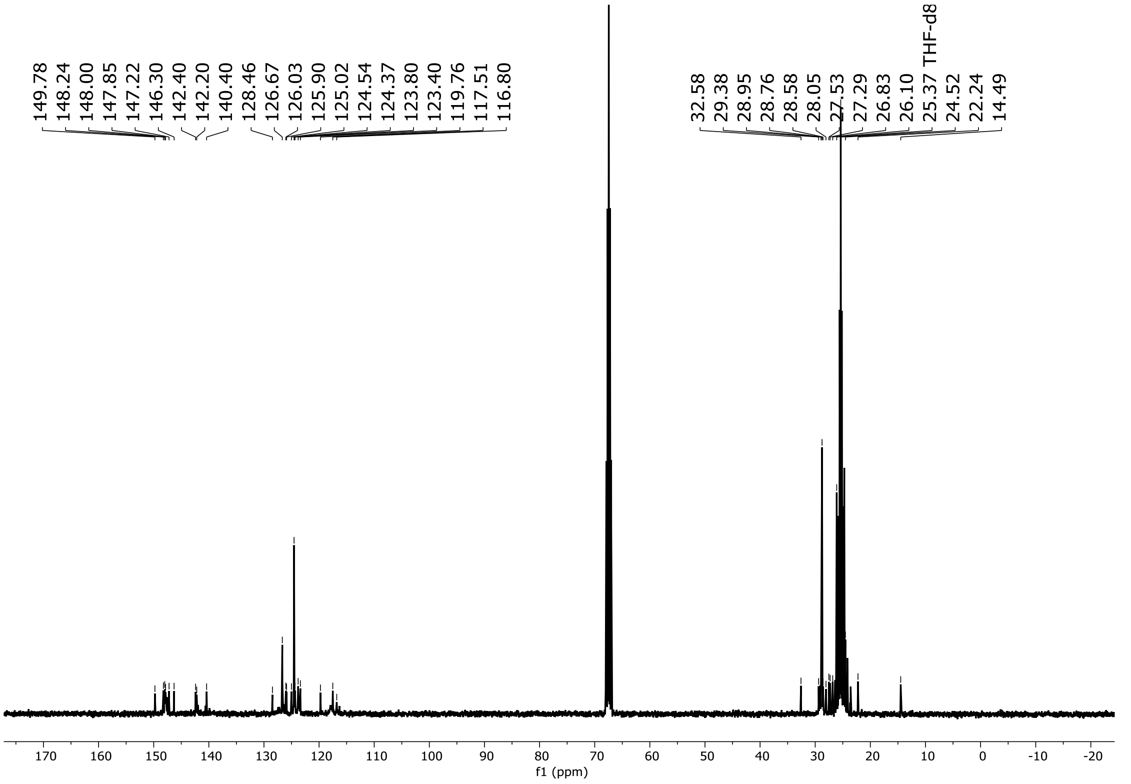

Figure S20. $^{13}C\{^1H\}$ NMR spectrum of compound **7** in THF-d$_8$.

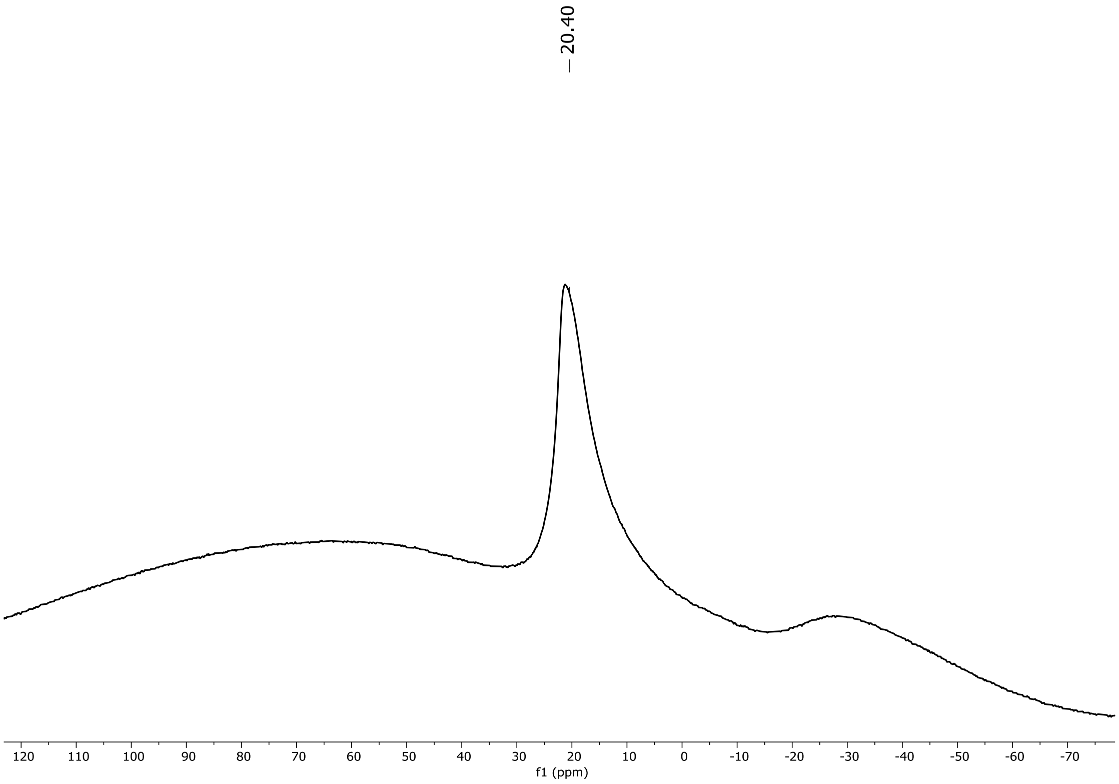

Figure S21. $^{11}B\{^1H\}$ NMR spectrum of compound **7** in THF-d$_8$.

K[(PhCCPh)Al{OB(NDippCH)$_2$}$_2$], 8

A mixture of compound **4** (80 mg, 0.08 mmol) and PhCCPh (14.9 mg, 0.08 mmol) in 0.5 mL of C_6D_6 was transferred to a high-pressure J-Young NMR tube and heated at 90 °C for 7 d. After cooling to room temperature, the reaction mixture was filtered and hexane (0.5 mL) added to the filtrate. The resulting solution was left to slowly evaporate at room temperature in a glove box, affording single crystals of **8** suitable for X-ray diffraction analysis. Yield: 5 mg, 0.004 mmol, ca. 5%.

^{1}H NMR (400 MHz, THF-d$_8$, 297 K): 0.80-1.01 (m, 48H, CH(C*H*$_3$)$_2$), 3.19-3.26 (m, 8H, C*H*(CH$_3$)$_2$), 5.57 (s, 4H, NC*H*), 6.41 (4H, Ph-*H)*, 6.41-6.44 (2H, Ph-*H)*, 6.57-6.61 (4H, Ph-*H)*, 7.00-7.16 (12H, Ar^{Dipp}-*H*).

^{11}B{^{1}H} NMR (128 MHz, THF-d$_8$): δ = 19.0.

^{13}C{^{1}H} NMR (100 MHz, THF-d$_8$): δ = 24.1-24.5 (CH(*C*H$_3$)$_2$), 28.7-32.9 (*C*H(CH$_3$)$_2$), 116.3 (N*C*H), 117.2 (*C*-Al), 117.3 (*C*-Al), 120.5 (Ar-*C*), 122.5 (Ar-*C*), 123.2 (Ar-*C*), 123.4 (Ar-*C*), 123.5 (Ar-*C*), 125.7 (Ar-*C*), 126.0 (Ar-*C*), 127.2 (Ar-*C*), 128.5 (Ar-*C*), 128.6 (Ar-*C*), 130.3 (Ar-*C*). 141.9 (Ar-*C*), 142.2 (Ar-*C*), 142.5 (Ar-*C*), 146.9 (Ar-*C*), 147.5 (Ar-*C*), 147.6 (Ar-*C*), 147.7 (Ar-*C*), 152.0 (Ar-*C*).

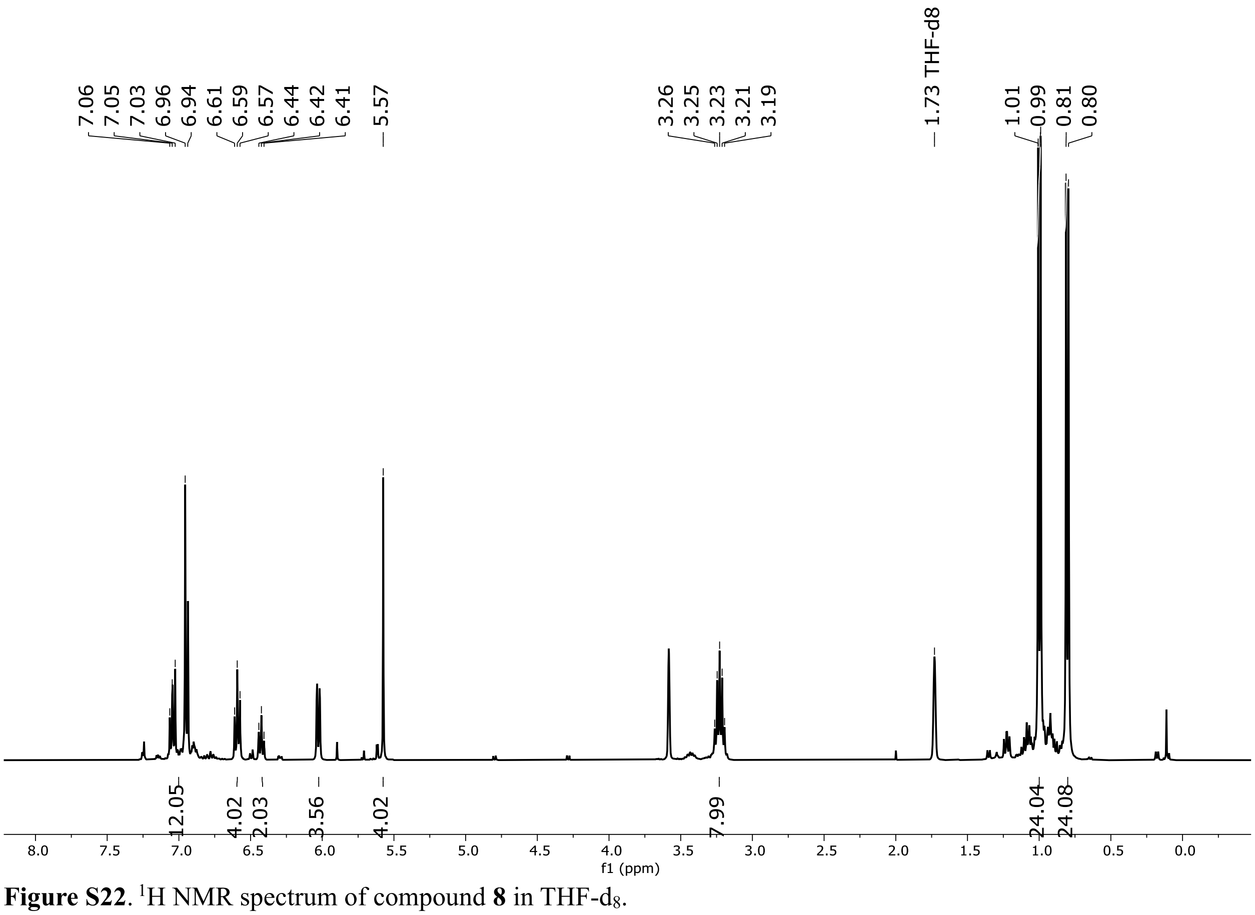

Figure S22. ^{1}H NMR spectrum of compound **8** in THF-d$_8$.

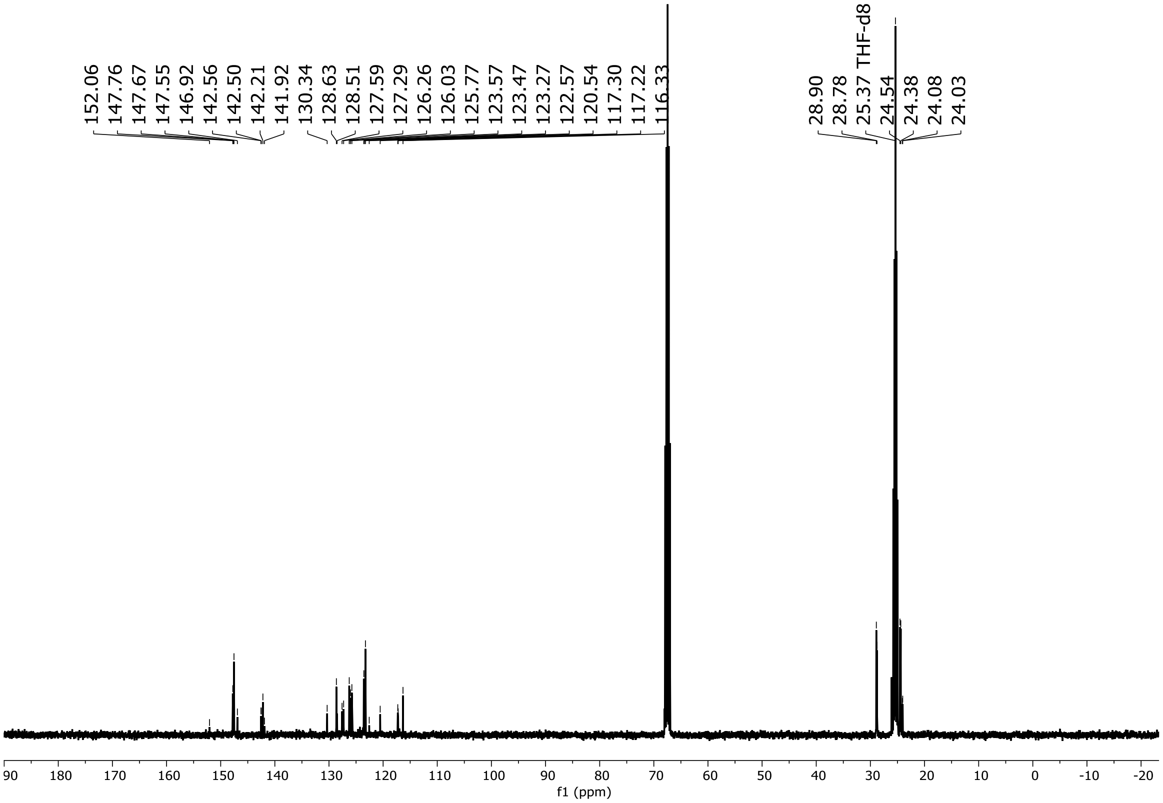

Figure S23. $^{13}C\{^{1}H\}$ NMR spectrum of compound **8** in THF-d_8.

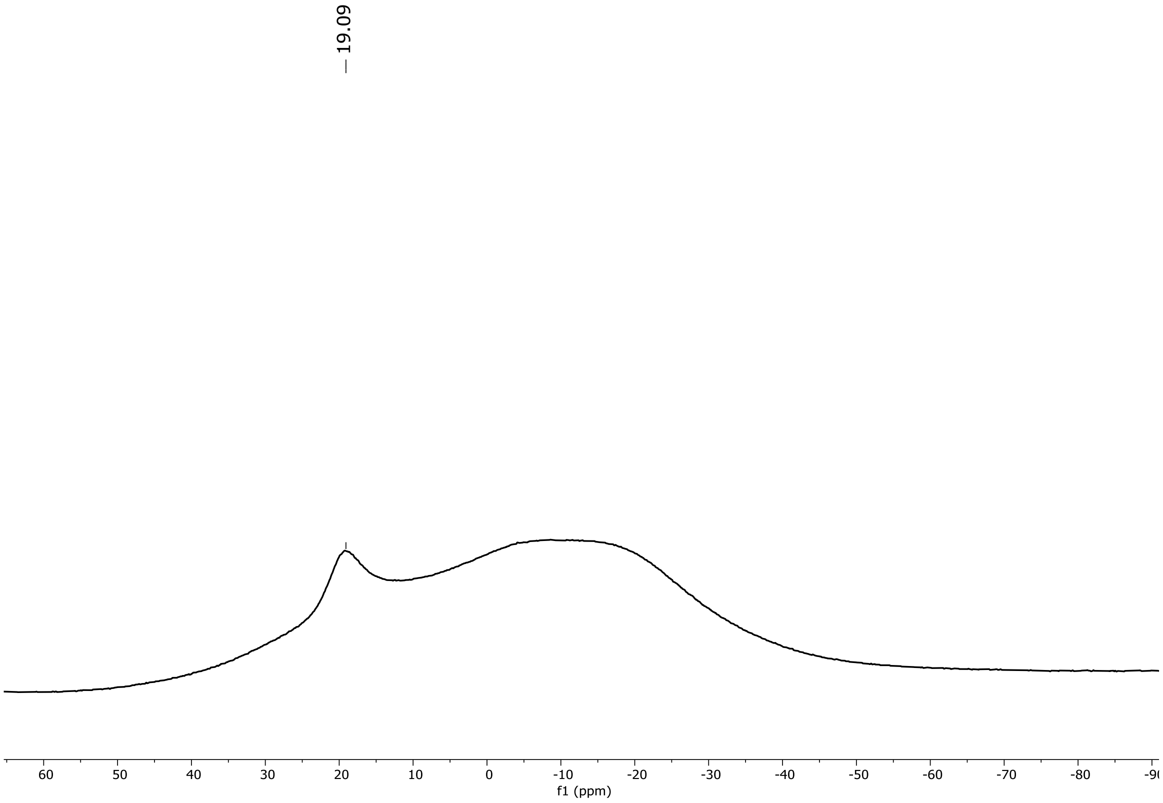

Figure S24. $^{11}B\{^{1}H\}$ NMR spectrum of compound **8** in THF-d_8.

Single Crystal X-ray Diffraction Studies

A Rigaku XtaLAB Synergy-DW VHF diffractometer equipped with a PhotonJet-R dual wavelength rotating anode and HyPix-Arc 150° detector was used to collect all crystallographic data. Crystals were mounted using Paratone-N or perfluorinated ether oil, mounted on MiTeGen Micromount loops, and quench-cooled with an Oxford Cryosystems open flow N_2 cooling device.[2-3] Specific data collection details are in given Tables S1-8. Data processing used CrysAlisPro for unit cell refinement, SCALE3 ABSPACK inter-frame scaling, merging of equivalent reflections and diffraction pattern processing. Structures were solved using SHELXT and refined using SHELXL in OLEX2.[4-6] Crystallographic data are available in in supplementary CIF files (2528441, 2528442, 2528443, 2528444, 2528447 2528448, 2528533 and 2530544) available free via the Cambridge Crystallographic Data Centre: http://www.ccdc.cam.ac.uk/data_request/cif.

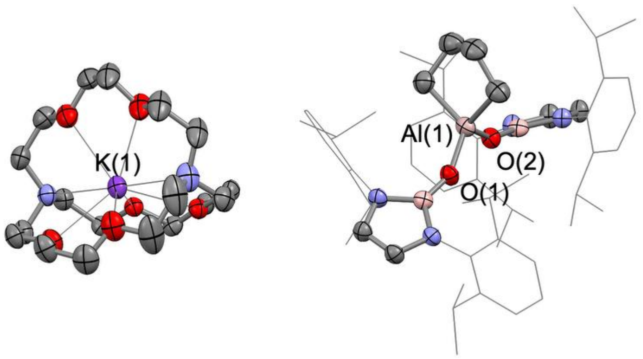

Figure S25. Molecular structure of compound **2'** in the solid state as determined by X-ray crystallography (ellipsoids set at 50% probability level; H atoms omitted and Dipp groups shown in wireframe format for clarity).

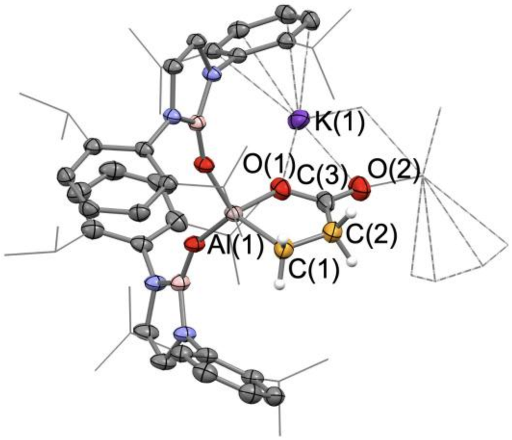

Figure S26. Molecular structure of compound **5** in the solid state as determined by X-ray crystallography (ellipsoids set at 50% probability level; H atoms omitted and ^{i}Pr groups shown in wireframe format for clarity).

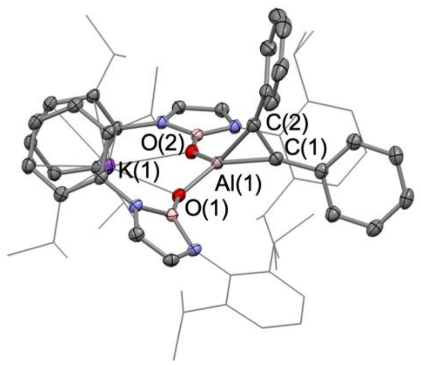

Figure S27. Molecular structure of compounds **8** in the solid state as determined by X-ray crystallography (ellipsoids set at 50% probability level; H atoms omitted and ^{i}Pr groups shown in wireframe format for clarity).

Table S1. Crystallographic details and refinement parameters for **2**

Identification code	2528441
Empirical formula	$C_{62}H_{86}AlB_2KN_4O_2$
Formula weight	1007.04
Temperature/K	100.00(10)
Crystal system	monoclinic
Space group	C2/c
a/Å	25.1197(2)
b/Å	12.67460(10)
c/Å	21.3333(2)
α/°	90
β/°	120.9570(10)
γ/°	90
Volume/Å^3	5824.62(10)
Z	4
ρ_{calc}/g cm^{-3}	1.148
μ/mm^{-1}	1.280
F(000)	2176.0
Crystal size/mm^3	0.12 × 0.11 × 0.06
Radiation	Cu Kα (λ = 1.54184)
2Θ range for data collection/°	8.094 to 151.802
Index ranges	$-31 \leq h \leq 31, -15 \leq k \leq 14, -26 \leq l \leq 26$
Reflections collected	104824
Independent reflections	6015 [R_{int} = 0.0286, R_{sigma} = 0.0091]
Data/restraints/parameters	6015/16/355
Goodness-of-fit on F^2	1.039
Final R indexes [I>=2σ (I)]	R_1 = 0.0417, wR_2 = 0.1123
Final R indexes [all data]	R_1 = 0.0423, wR_2 = 0.1128
Largest diff. peak, hole/e Å^{-3}	0.59/-0.43

Table S2. Crystallographic details and refinement parameters for **2'**

Identification code	2528442
Empirical formula	$C_{77}H_{123}AlB_2KN_6O_8$
Formula weight	1348.51
Temperature/K	99.99(10)
Crystal system	monoclinic
Space group	$P2_1$
a/Å	12.65220(10)
b/Å	49.9524(4)
c/Å	13.25310(10)
α/°	90
β/°	105.1500(10)
γ/°	90
Volume/Å^3	8084.95(12)
Z	4
ρ_{calc}/g cm^{-3}	1.108
μ/mm^{-1}	1.097
F(000)	2932.0

Crystal size/mm^3	0.05 × 0.02 × 0.02
Radiation	Cu Kα (λ = 1.54184)
2Θ range for data collection/°	3.538 to 152.578
Index ranges	-15 ≤ h ≤ 15, -62 ≤ k ≤ 55, -16 ≤ l ≤ 16
Reflections collected	259074
Independent reflections	32061 [R_{int} = 0.1062, R_{sigma} = 0.0468]
Data/restraints/parameters	32061/3/1766
Goodness-of-fit on F^2	1.060
Final R indexes [I>=2σ (I)]	R_1 = 0.0774, wR_2 = 0.2155
Final R indexes [all data]	R_1 = 0.0861, wR_2 = 0.2239
Largest diff. peak, hole/e Å^{-3}	0.62/-0.36
Flack parameter	0.015(8)

Table S3. Crystallographic details and refinement parameters for **3**

Identification code	2528533
Empirical formula	$C_{61}H_{84}AlB_2KN_4O_2$
Formula weight	993.02
Temperature/K	100.00(10)
Crystal system	monoclinic
Space group	C2/c
a/Å	25.0129(3)
b/Å	12.67160(10)
c/Å	21.3049(3)
α/°	90
β/°	120.614(2)
γ/°	90
Volume/Å^3	5811.46(16)
Z	4
ρ_{calc}/g cm^{-3}	1.135
μ/mm-^{1}	1.276
F(000)	2144.0
Crystal size/mm3	0.12 × 0.12 × 0.03
Radiation	Cu Kα (λ = 1.54184)
2Θ range for data collection/°	8.096 to 152.008
Index ranges	-31 ≤ h ≤ 31, -15 ≤ k ≤ 15, -26 ≤ l ≤ 25
Reflections collected	47967
Independent reflections	5965 [Rint = 0.0404, Rsigma = 0.0176]
Data/restraints/parameters	5965/112/367
Goodness-of-fit on F^2	1.046
Final R indexes [I>=2σ (I)]	R1 = 0.0413, wR2 = 0.1131
Final R indexes [all data]	R1 = 0.0426, wR2 = 0.1143
Largest diff. peak, hole/e Å^{-3}	0.32/-0.48

Table S4. Crystallographic details and refinement parameters for **4**

Identification code	2528448
Empirical formula	$C_{64}H_{90}AlB_2KN_4O_2$
Formula weight	1035.09

Temperature/K	100.00(10)
Crystal system	monoclinic
Space group	C2/c
a/Å	24.6596(3)
b/Å	13.34410(10)
c/Å	23.2641(3)
α/°	90
β/°	128.773(2)
γ/°	90
Volume/Å^3	5968.31(17)
Z	4
ρ_{calc}/g cm^{-3}	1.152
μ/mm^{-1}	1.261
F(000)	2240.0
Crystal size/mm^3	0.07 × 0.05 × 0.04
Radiation	Cu Kα (λ = 1.54184)
2Θ range for data collection/°	7.792 to 151.69
Index ranges	$-30 \leq h \leq 29$, $-16 \leq k \leq 16$, $-24 \leq l \leq 28$
Reflections collected	66977
Independent reflections	6139 [R_{int} = 0.0241, R_{sigma} = 0.0117]
Data/restraints/parameters	6139/33/337
Goodness-of-fit on F^2	1.051
Final R indexes [I>=2σ (I)]	R_1 = 0.0400, wR_2 = 0.1053
Final R indexes [all data]	R_1 = 0.0417, wR_2 = 0.1066
Largest diff. peak, hole/e Å^{-3}	0.57/-0.58

Table S5. Crystallographic details and refinement parameters for **5**

Identification code	2528444
Empirical formula	$C_{110}H_{152}Al_2B_4K_2N_8O_8$
Formula weight	1889.79
Temperature/K	100.00(10)
Crystal system	monoclinic
Space group	$P2_1/c$
a/Å	12.18800(10)
b/Å	23.0086(3)
c/Å	19.5951(2)
α/°	90
β/°	92.7280(10)
γ/°	90
Volume/Å^3	5488.80(10)
Z	2
ρ_{calc}/g cm^{-3}	1.143
μ/mm^{-1}	1.356
F(000)	2032.0
Crystal size/mm^3	0.34 × 0.07 × 0.03
Radiation	Cu Kα (λ = 1.54184)
2Θ range for data collection/°	5.928 to 151.972
Index ranges	$-11 \leq h \leq 15$, $-28 \leq k \leq 28$, $-24 \leq l \leq 24$

Reflections collected	19703
Independent reflections	19703 [R_{int} = ?, R_{sigma} = 0.0105]
Data/restraints/parameters	19703/32/655
Goodness-of-fit on F^2	1.044
Final R indexes [I>=2σ (I)]	R_1 = 0.0507, wR_2 = 0.1404
Final R indexes [all data]	R_1 = 0.0550, wR_2 = 0.1436
Largest diff. peak, hole/e Å^{-3}	0.46/-0.38

Table S6. Crystallographic details and refinement parameters for **6**

Identification code	2528447
Empirical formula	$C_{62}H_{82}AlB_2KN_4O_2$
Formula weight	1003.01
Temperature/K	100.00(10)
Crystal system	triclinic
Space group	P-1
a/Å	12.4695(3)
b/Å	13.1946(4)
c/Å	18.8665(4)
α/°	90.780(2)
β/°	108.604(2)
γ/°	94.732(2)
Volume/Å^3	2929.26(13)
Z	2
ρ_{calc}/g cm^{-3}	1.137
μ/mm^{-1}	1.272
F(000)	1080.0
Crystal size/mm^3	0.08 × 0.04 × 0.01
Radiation	Cu Kα (λ = 1.54184)
2Θ range for data collection/°	4.946 to 148.64
Index ranges	-15 ≤ h ≤ 15, -16 ≤ k ≤ 16, -23 ≤ l ≤ 23
Reflections collected	42827
Independent reflections	11461 [R_{int} = 0.0644, R_{sigma} = 0.0737]
Data/restraints/parameters	11461/26/687
Goodness-of-fit on F^2	1.042
Final R indexes [I>=2σ (I)]	R_1 = 0.0658, wR_2 = 0.1623
Final R indexes [all data]	R_1 = 0.1224, wR_2 = 0.1918
Largest diff. peak, hole/e Å^{-3}	0.35/-0.66

Table S7. Crystallographic details and refinement parameters for **7**

Identification code	2528443
Empirical formula	$C_{62}H_{84}AlB_2KN_4O_3$
Formula weight	1021.03
Temperature/K	99.98(10)
Crystal system	monoclinic
Space group	C2/c
a/Å	24.8130(3)
b/Å	13.13320(10)

c/Å	20.9626(3)
α/°	90
β/°	119.671(2)
γ/°	90
Volume/Å^3	5935.48(16)
Z	4
ρ_{calc}/g cm^{-3}	1.143
μ/mm^{-1}	1.276
F(000)	2200.0
Crystal size/mm^3	0.24 × 0.12 × 0.03
Radiation	Cu Kα (λ = 1.54184)
2Θ range for data collection/°	7.882 to 152.114
Index ranges	-30 ≤ h ≤ 30, -16 ≤ k ≤ 16, -26 ≤ l ≤ 26
Reflections collected	38855
Independent reflections	6095 [R_{int} = 0.0209, R_{sigma} = 0.0134]
Data/restraints/parameters	6095/666/465
Goodness-of-fit on F^2	1.036
Final R indexes [I>=2σ (I)]	R_1 = 0.0435, wR_2 = 0.1161
Final R indexes [all data]	R_1 = 0.0456, wR_2 = 0.1177
Largest diff. peak, hole/e Å^{-3}	0.33/-0.32

Table S8. Crystallographic details and refinement parameters for **8**

Identification code	2530544
Empirical formula	$C_{66}H_{82}B_2N_4O_2AlK$
Formula weight	1051.05
Temperature/K	100(2)
Crystal system	triclinic
Space group	P-1
a/Å	12.6359(2)
b/Å	12.7078(2)
c/Å	19.5636(2)
α/°	83.6190(10)
β/°	84.3180(10)
γ/°	73.0100(10)
Volume/Å^3	2978.38(8)
Z	2
ρ_{calc}/g cm^{-3}	1.172
μ/mm^{-1}	1.275
F(000)	1128.0
Crystal size/mm^3	0.15 × 0.11 × 0.07
Radiation	CuKα (λ = 1.54184)
2Θ range for data collection/°	4.556 to 140.138
Index ranges	-15 ≤ h ≤ 15, -12 ≤ k ≤ 15, -23 ≤ l ≤ 23
Reflections collected	46830
Independent reflections	11246 [R_{int} = 0.0200, R_{sigma} = 0.0156]
Data/restraints/parameters	11246/0/701
Goodness-of-fit on F^2	1.058
Final R indexes [I>=2σ (I)]	R_1 = 0.0318, wR_2 = 0.0852

Final R indexes [all data]	R_1 = 0.0346, wR_2 = 0.0868
Largest diff. peak, hole/e $Å^{-3}$	0.34/-0.25

Computational Details

All computational work was carried out at the density functional theory (DFT) level, using Gaussian16 (Revision C.02).[7] The exchange correlation functional PBE1PBE[8-10] was employed in conjunction with the Def2-SVP[11-12] basis set, Grimme's empirical dispersion correction (GD3BJ)[13] and an ultrafine integration grid for the optimization and frequency calculations for the mechanistic studies with ethene and propene. Single-point calculations were performed for the Def2-SVP optimized geometries at the PBE1PBE-GD3BJ/Def2-TZVP (PCM, solvent = benzene) level.[10,12,14] The reported Gibbs free energies correspond to these corrected single point energies. The nature of the stationary points, minima and transition states, was confirmed by frequency calculations at the Def2-SVP level, and are characterized by zero or one imaginary frequency, respectively.

Optimized xyz Coordinates

```
  134
K[Al(OBoryl)2]
 C      2.155750    -1.906477    -3.460936
 C      2.386914    -2.868459    -2.311637
 H      2.915901    -1.115610    -3.372343
 C      0.788539    -1.233009    -3.319650
 C      1.894472    -4.178619    -2.355990
 C      2.043182    -5.036166    -1.266548
 C      2.707359    -4.599963    -0.122302
 C      3.225014    -3.300633    -0.039168
 C      3.037548    -2.430081    -1.135976
 N      3.423656    -1.080565    -1.020156
 B      2.629781    -0.030412    -0.399504
 N      3.463669     1.147549    -0.522324
 C      4.652884     0.790643    -1.156577
 C      4.633300    -0.530778    -1.461080
 C      3.926940    -2.825521     1.217913
 C      2.913851    -2.465620     2.307443
 C      3.193446     2.422885     0.035271
 C      2.550735     3.399261    -0.750906
 C      2.221704     4.618493    -0.153136
 C      2.534234     4.868147     1.179170
 C      3.176468     3.897810     1.939384
 C      3.508393     2.657751     1.388492
 C      2.167224     3.099967    -2.186370
 C      0.773898     2.472423    -2.244363
 C      4.197851     1.596480     2.222488
 C      3.610382     1.469547     3.624744
 C      2.250583     4.318196    -3.100696
 C      4.958728    -3.829803     1.725216
 C      5.707772     1.836734     2.262218
 O      1.404012    -0.175449     0.133812
Al      0.287855     0.863529     1.196290
 O     -1.181412     0.024734     0.431380
 B     -2.504019     0.206274     0.585596
 N     -3.340520     1.357150     0.307643
 C     -4.670210     1.005001     0.551107
 C     -4.740498    -0.282433     0.969990
 N     -3.448755    -0.813918     1.023263
 C     -3.108539    -2.163968     1.226758
 C     -2.371262    -2.518414     2.384148
 C     -1.954626    -3.846462     2.525539
 C     -2.261852    -4.807113     1.561031
 C     -2.982521    -4.445304     0.427505
 C     -3.401029    -3.121770     0.230870
 C     -2.070973    -1.474938     3.442077
 C     -3.312213    -1.213611     4.297593
 C     -4.120115    -2.735847    -1.047615
 C     -3.313624    -3.110251    -2.290635
 C     -2.958350     2.566151    -0.329261
 C     -3.101635     2.667722    -1.728718
 C     -2.720905     3.858370    -2.352582
 C     -2.191205     4.911175    -1.613606
 C     -2.039193     4.789634    -0.236764
 C     -2.425874     3.624583     0.434125
 C     -3.582027     1.478100    -2.538602
 C     -4.405905     1.862291    -3.762603
 C     -2.327873     3.518823     1.942949
 C     -3.671872     3.861461     2.589587
 C     -1.198085     4.347165     2.540120
 C     -0.860740    -1.802626     4.306507
 C     -2.389826     0.601018    -2.929423
 C     -5.525368    -3.334561    -1.095816
 K     -0.113787    -2.330213    -0.123700
 H     -2.104951     2.463858     2.172856
 H     -1.936764    -5.841588     1.697308
 H     -3.213281    -5.200523    -0.328648
 H     -1.834637    -0.540118     2.909213
 H     -4.232305     0.877873    -1.882953
 H      2.832214    -5.284081     0.720630
 H     -4.225340    -1.641088    -1.042974
 H     -1.612321     5.617729     0.331210
 H      2.884581     2.351700    -2.559666
 H     -1.887340     5.832554    -2.116961
 H     -2.823355     3.959384    -3.434901
 H     -1.384532    -4.139117     3.409102
 H      1.706211     5.383608    -0.736802
 H      5.434990     1.521690    -1.351535
 H      2.266173     5.826160     1.631416
 H     -0.235367     4.105681     2.064642
 H     -1.100878     4.124317     3.613507
 H     -1.382258     5.430055     2.446995
 H      5.390682    -1.131265    -1.960471
 H     -1.714481     1.147079    -3.606759
 H     -2.725736    -0.311073    -3.448408
 H     -1.804699     0.309609    -2.044544
 H      3.404962     4.099811     2.987917
 H      4.461783    -1.901613     0.950433
 H     -5.493252    -4.436142    -1.103072
 H     -6.057836    -3.007165    -2.002302
 H     -6.118545    -3.023964    -0.223190
 H     -2.324348    -2.625357    -2.290912
 H     -3.833285    -2.777121    -3.202106
 H     -3.161615    -4.197889    -2.376605
 H     -1.034077    -2.672764     4.960443
 H     -0.625771    -0.945826     4.954689
 H      0.034670    -2.000691     3.696342
 H      4.032471     0.635534     1.710647
 H      1.650721    -6.055060    -1.315834
 H     -5.243887     2.523688    -3.495140
 H     -4.821706     0.960871    -4.239129
 H     -3.799524     2.377200    -4.524625
 H      1.385068    -4.534421    -3.254612
 H     -3.957850     4.903076     2.370035
 H     -3.614545     3.748424     3.683894
 H     -4.475125     3.204976     2.225014
 H     -4.163808    -0.896432     3.678851
 H     -3.112281    -0.415896     5.029558
 H     -3.605981    -2.120323     4.850898
 H      6.136804     1.853082     1.249332
```

H	6.216121	1.043759	2.834184
H	5.938720	2.801824	2.741780
H	3.820318	2.357157	4.242835
H	4.049656	0.602931	4.144210
H	2.518354	1.333156	3.577656
H	4.489566	-4.756922	2.091731
H	5.524145	-3.399574	2.565830
H	5.676248	-4.103268	0.936992
H	1.474361	5.062275	-2.861556
H	2.093619	4.017682	-4.148244
H	3.230409	4.814730	-3.030464
H	0.721502	1.554905	-1.642488
H	0.503796	2.215709	-3.281184
H	0.010722	3.164618	-1.857150
H	2.237342	-1.665699	1.971132
H	3.424896	-2.102614	3.212598
H	2.311279	-3.344247	2.594963
H	-5.479439	1.721229	0.422915
H	-5.614308	-0.854075	1.276013
C	2.313255	-2.557438	-4.831043
H	0.621250	-0.501723	-4.124819
H	-0.025557	-1.976490	-3.380576
H	0.705686	-0.686776	-2.368366
H	1.516940	-3.290703	-5.036583
H	2.257594	-1.793617	-5.621363
H	3.280454	-3.073846	-4.924357

****

6
ethene

H	0.018672	0.000000	-0.012525
C	-0.024633	0.000000	1.080932
H	0.933475	0.000000	1.609604
C	-1.183615	-0.000000	1.734470
H	-1.226867	-0.000000	2.827932
H	-2.141760	-0.000000	1.205848

****

9
propene

H	-0.065800	-0.204806	0.183884
C	-1.109859	0.065799	-0.032591
H	-1.568923	-0.761838	-0.599010
H	-1.106034	0.938928	-0.706490
C	-1.876528	0.353312	1.215321
C	-1.386909	0.298325	2.454698
H	-2.930125	0.631265	1.081077
H	-2.008832	0.522334	3.325298
H	-0.343319	0.026143	2.645334

****

140
TS1 ethene

C	3.393599	-4.281107	-2.014425
C	3.662075	-2.972049	-1.604119
C	3.369333	-2.613889	-0.273241
C	2.832524	-3.543302	0.640775
C	2.583319	-4.841649	0.185566
C	2.857183	-5.208177	-1.127907
N	3.617156	-1.280454	0.143898
B	2.709976	-0.148023	0.134791
N	3.548879	0.959521	0.583816
C	4.841005	0.481386	0.828389
C	4.875950	-0.845224	0.559827
C	2.580131	-3.161299	2.086358
C	1.394440	-3.891721	2.704148
C	4.208317	-1.941783	-2.574349
C	3.061655	-1.214079	-3.277853
C	3.128162	2.272517	0.873955
C	2.567491	2.556970	2.138626
C	2.057647	3.841695	2.369922
C	2.121092	4.826117	1.386636
C	2.707397	4.542994	0.153539
C	3.220520	3.269925	-0.123121
C	2.481682	1.489727	3.211077
C	1.055015	0.957316	3.348730
C	3.793765	2.934962	-1.486371
C	4.605624	4.073606	-2.094023
C	2.678249	2.482311	-2.432709
C	3.026839	1.978457	4.551240
C	5.192840	-2.522874	-3.583987
C	3.847385	-3.353479	2.922779
O	1.407967	-0.057654	-0.201026
Al	-0.021349	-1.174872	0.068003
C	-0.514720	-1.899470	-2.245704
C	-0.290128	-2.804772	-1.175441
H	0.649750	-3.366897	-1.143781
O	-1.302401	0.129750	0.000392
B	-2.638065	0.209843	-0.096184
N	-3.682058	-0.750662	0.209399
C	-4.919618	-0.140948	-0.004042
C	-4.742485	1.132084	-0.430160
N	-3.371713	1.396391	-0.519031
C	-2.810547	2.609489	-0.959562
C	-2.162752	2.651519	-2.217565
C	-1.566582	3.854632	-2.615242
C	-1.603436	4.981403	-1.794925
C	-2.226177	4.919846	-0.550547
C	-2.827751	3.735469	-0.106789
C	-2.112503	1.409909	-3.086826
C	-0.969847	1.405148	-4.094240
C	-3.385679	3.608842	1.298901
C	-3.899126	4.920296	1.879857
C	-3.529704	-2.094033	0.632182
C	-3.895292	-3.146458	-0.230522
C	-3.717876	-4.460569	0.216352
C	-3.168412	-4.725330	1.463591
C	-2.797966	-3.674814	2.298501
C	-2.977248	-2.346553	1.908170
C	-4.472798	-2.890737	-1.610285
C	-3.762339	-3.687323	-2.704185
C	-2.641762	-1.194841	2.839396
C	-1.658770	-1.563597	3.941615
C	-2.332599	2.976095	2.215227
C	-3.456395	1.167366	-3.776510
C	-3.915311	-0.582783	3.427928
C	-5.977097	-3.170389	-1.631616
K	0.172873	2.298877	-0.144222
H	-1.065432	3.912016	-3.583219
H	-5.492006	1.873954	-0.698071
H	-5.852177	-0.670059	0.176854
H	-2.234151	5.804062	0.089649
H	-4.232130	2.906496	1.254074
H	-1.929409	0.562656	-2.408195
H	-1.138954	5.913217	-2.127135
H	-3.694041	1.988059	-4.472599
H	-4.274856	1.085263	-3.047291
H	-3.424240	0.229706	-4.352920
H	-2.148557	-0.426142	2.223821
H	-4.325331	-1.822605	-1.832728
H	-3.996976	-5.290547	-0.437068
H	-3.021012	-5.758059	1.788821
H	-0.919921	0.423438	-4.587386
H	0.005279	1.574393	-3.611475
H	-1.100149	2.163448	-4.883364
H	-2.358488	-3.896713	3.271718
H	-1.442770	3.624762	2.296430
H	-2.021040	1.986969	1.846884
H	-2.729833	2.832113	3.231684
H	-4.603738	-0.240416	2.642199
H	-3.670020	0.283011	4.064417
H	-4.447475	-1.319474	4.050982

H	-4.399587	4.736263	2.842316
H	-4.623371	5.406077	1.208636
H	-3.083647	5.635540	2.073790
H	-2.086253	-2.285847	4.655710
H	-1.387504	-0.664645	4.516057
H	-0.737532	-1.990197	3.517604
H	-6.180390	-4.233651	-1.425068
H	-6.405736	-2.931112	-2.617747
H	-6.514851	-2.580413	-0.874888
H	-2.680871	-3.488275	-2.716193
H	-4.167706	-3.415158	-3.691690
H	-3.910487	-4.771915	-2.581350
H	-1.132064	-3.376423	-0.769527
H	-1.521736	-1.547249	-2.480718
H	0.300341	-1.555328	-2.883103
H	1.607986	4.075372	3.338290
H	5.637122	1.133243	1.181598
H	5.719222	-1.530106	0.621346
H	2.763747	5.325527	-0.606352
H	4.465781	2.074920	-1.344354
H	3.113831	0.652527	2.879835
H	1.720480	5.823543	1.584239
H	2.402961	2.775906	4.985735
H	4.050942	2.368169	4.450051
H	3.047826	1.150572	5.276170
H	4.753606	-1.194211	-1.977199
H	2.333249	-2.086852	2.087973
H	2.156859	-5.577969	0.868641
H	2.645733	-6.225935	-1.464763
H	1.004185	0.181901	4.127483
H	0.703203	0.489257	2.414982
H	0.348780	1.757515	3.626596
H	3.602297	-4.579394	-3.043624
H	1.937433	3.287436	-2.582552
H	2.167426	1.586102	-2.048607
H	3.082147	2.221567	-3.422682
H	6.000875	-3.080996	-3.087064
H	5.650143	-1.715367	-4.176560
H	4.700477	-3.203924	-4.296032
H	5.103134	3.733718	-3.014866
H	5.381350	4.431683	-1.400618
H	3.974087	4.933569	-2.368970
H	2.479613	-1.914753	-3.897833
H	3.445853	-0.416269	-3.933768
H	2.371103	-0.765116	-2.550152
H	4.155976	-4.411494	2.928073
H	3.674027	-3.046044	3.966680
H	4.684836	-2.758509	2.530972
H	0.489781	-3.744049	2.093082
H	1.194080	-3.495847	3.711982
H	1.582035	-4.971750	2.815648

****

146

K[(C2H4)Al(OBoryl)2] + ethene

C	4.068084	-1.474940	-1.990939
C	4.075437	-1.588074	-0.580101
C	4.316239	-2.826593	0.050799
C	4.503991	-3.956713	-0.752625
C	4.458100	-3.867910	-2.137060
C	4.249559	-2.634735	-2.747102
N	3.854189	-0.423409	0.200146
C	4.891090	0.232193	0.871147
C	4.450257	1.404630	1.381400
N	3.097673	1.555223	1.069021
C	2.353548	2.730179	1.291904
C	1.652721	2.886417	2.508396
C	0.838544	4.012350	2.665147
C	0.728469	4.965639	1.652689
C	1.459500	4.821469	0.476385
C	2.292629	3.710797	0.278796
C	1.790084	1.828740	3.585872
C	1.630196	2.377434	4.998834
C	3.112408	3.579070	-0.991338
C	2.245042	3.322886	-2.225036
C	4.434827	-2.951556	1.558792
C	5.907378	-2.997771	1.978288
C	3.923755	-0.118645	-2.663809
C	5.193116	0.719491	-2.476757
B	2.652362	0.396573	0.304110
O	1.406064	0.222802	-0.153330
Al	0.161623	-0.642668	-1.090315
C	0.105053	0.028409	-2.964433
C	0.457310	-1.497660	-2.779724
O	-1.362724	-0.320033	-0.204591
B	-2.697103	-0.469401	-0.143246
N	-3.714027	0.545088	-0.437067
C	-3.534279	1.918030	-0.718576
C	-3.335735	2.348441	-2.050934
C	-3.100646	3.711047	-2.277860
C	-3.080305	4.624816	-1.226002
C	-3.311715	4.193256	0.077618
C	-3.546917	2.840489	0.352542
C	-3.412162	1.361564	-3.198544
C	-4.870261	1.025901	-3.524667
C	-3.746151	2.355483	1.775387
C	-2.401716	1.966657	2.388720
N	-3.486826	-1.615901	0.283369
C	-4.839754	-1.277981	0.228637
C	-4.977547	0.001972	-0.183517
C	-3.065555	-2.903764	0.700440
C	-2.536981	-3.075606	1.997616
C	-2.123618	-4.356341	2.374928
C	-2.246025	-5.435291	1.506245
C	-2.794601	-5.254628	0.241967
C	-3.216059	-3.991553	-0.183983
C	-2.447655	-1.900740	2.957979
C	-3.830692	-1.502051	3.481440
C	-3.773553	-3.777106	-1.578972
C	-4.503324	-4.993255	-2.138391
C	-2.667940	-3.314350	-2.526413
C	-1.506732	-2.135063	4.134951
C	-2.682209	1.827119	-4.452519
C	-4.484959	3.352711	2.660585
C	0.834084	0.659382	3.340769
C	4.020583	4.791226	-1.191436
C	3.698271	-4.163920	2.127591
C	3.579925	-0.187882	-4.147142
K	-0.416153	2.305007	-0.543259
H	-0.880510	0.127997	-3.442011
H	0.285169	4.153153	3.595721
H	4.997502	2.153198	1.950523
H	5.891087	-0.190628	0.919704
H	1.393781	5.591292	-0.297597
H	3.759293	2.700127	-0.862105
H	2.812937	1.430939	3.492982
H	0.083796	5.837097	1.791936
H	0.596600	2.702757	5.199230
H	2.298098	3.232188	5.185568
H	1.866355	1.594450	5.735162
H	3.090816	0.400673	-2.162298
H	3.989317	-2.043973	1.996969
H	4.684534	-4.925252	-0.281073
H	4.593598	-4.763244	-2.748560
H	1.070367	-0.179849	4.012545
H	0.890557	0.295698	2.305572
H	-0.205251	0.960478	3.537197
H	4.229081	-2.575895	-3.835825
H	1.500017	4.123929	-2.374970
H	1.726984	2.351749	-2.177277
H	2.867590	3.292487	-3.132220
H	5.438728	0.881100	-1.418646

H	5.073446	1.707151	-2.950671
H	6.052531	0.222272	-2.954529
H	4.686050	4.630878	-2.053715
H	4.648453	4.968410	-0.305542
H	3.444304	5.710303	-1.385178
H	4.410464	-0.607402	-4.737592
H	3.393340	0.827142	-4.529225
H	2.677138	-0.785294	-4.335994
H	6.397050	-3.896364	1.569773
H	6.000700	-3.030973	3.075417
H	6.468943	-2.125327	1.616360
H	2.647835	-4.194977	1.807740
H	3.720231	-4.136727	3.228321
H	4.174869	-5.108267	1.820815
H	0.829451	0.596024	-3.574208
H	1.517634	-1.733142	-2.948558
H	-0.161710	-2.218030	-3.330769
H	-2.934998	4.064819	-3.296630
H	-5.887547	0.582656	-0.318812
H	-5.614103	-1.993870	0.496974
H	-3.309696	4.918735	0.893621
H	-4.356025	1.440704	1.722576
H	-2.927735	0.436344	-2.848718
H	-2.896081	5.683276	-1.426777
H	-5.418038	1.928798	-3.839586
H	-5.391075	0.588788	-2.662281
H	-4.916618	0.296350	-4.348072
H	-2.052855	-1.050077	2.377559
H	-4.506535	-2.958054	-1.514165
H	-2.892415	-6.110585	-0.428065
H	-1.912251	-6.427276	1.819933
H	-2.649594	1.011035	-5.188930
H	-1.642419	2.115859	-4.239945
H	-3.192790	2.677814	-4.932413
H	-1.695524	-4.517121	3.365096
H	-1.731013	2.838998	2.459261
H	-1.897670	1.188370	1.796799
H	-2.535594	1.567519	3.406090
H	-4.515907	-1.224901	2.669280
H	-3.749710	-0.640593	4.163905
H	-4.283270	-2.334397	4.043768
H	-4.716685	2.892195	3.632885
H	-5.432335	3.677021	2.204240
H	-3.882277	4.251736	2.866901
H	-1.900552	-2.901149	4.821641
H	-1.395528	-1.206850	4.715108
H	-0.503935	-2.453062	3.814190
H	-3.814595	-5.826071	-2.351793
H	-4.994138	-4.733401	-3.088714
H	-5.275358	-5.361420	-1.445375
H	-2.155087	-2.421005	-2.146440
H	-3.074632	-3.076262	-3.522053
H	-1.902246	-4.096852	-2.649379
C	0.595811	-3.110913	0.011839
H	1.434650	-3.420571	-0.617416
C	0.782542	-2.388544	1.123964
H	-0.398585	-3.466277	-0.273496
H	-0.058401	-2.081032	1.745187
H	1.774952	-2.047100	1.425143

****

146

TS2 ethene

C	-4.101042	-1.638065	1.864919
C	-4.065680	-1.637785	0.450469
C	-4.235463	-2.829005	-0.284709
C	-4.434083	-4.021553	0.419644
C	-4.455708	-4.039981	1.807606
C	-4.290370	-2.856176	2.520851
N	-3.865653	-0.408197	-0.226557
C	-4.897727	0.251264	-0.901091
C	-4.480186	1.470480	-1.314500
N	-3.152140	1.650071	-0.923008
C	-2.393869	2.819649	-1.119667
C	-1.688014	2.991096	-2.331664
C	-0.849470	4.103378	-2.457644
C	-0.715374	5.023873	-1.418204
C	-1.446584	4.862591	-0.244052
C	-2.306906	3.767859	-0.077708
C	-1.834813	1.959730	-3.434353
C	-1.661746	2.541108	-4.832891
C	-3.113810	3.602408	1.197242
C	-2.250528	3.119874	2.365633
C	-4.241299	-2.842164	-1.802105
C	-5.672493	-2.938220	-2.337470
C	-3.970956	-0.338757	2.643120
C	-5.240246	0.509123	2.515396
B	-2.697043	0.460531	-0.217509
O	-1.471750	0.303730	0.301965
Al	-0.197887	-0.837288	0.861635
C	-0.065393	-0.468242	2.835589
C	-0.518074	-1.900969	2.578323
O	1.389453	-0.294016	0.182855
B	2.719275	-0.412850	0.056306
N	3.718597	0.635006	0.286095
C	3.506734	1.986151	0.631664
C	3.244149	2.331567	1.977564
C	2.984801	3.675188	2.278474
C	2.994275	4.650343	1.283020
C	3.271743	4.299307	-0.035799
C	3.532325	2.968731	-0.384179
C	3.263071	1.267100	3.056638
C	4.701185	0.894878	3.425251
C	3.758012	2.559133	-1.826751
C	2.445203	2.064343	-2.434879
N	3.524286	-1.546292	-0.374603
C	4.867712	-1.169892	-0.387341
C	4.986243	0.122752	-0.007790
C	3.109786	-2.862382	-0.696006
C	2.504154	-3.112219	-1.944606
C	2.087714	-4.416764	-2.223923
C	2.279563	-5.442451	-1.304873
C	2.899151	-5.183999	-0.086860
C	3.325145	-3.893917	0.241212
C	2.331258	-1.988756	-2.953535
C	3.665258	-1.624285	-3.610562
C	3.960580	-3.586765	1.585454
C	4.697726	-4.771827	2.198657
C	2.920515	-3.029407	2.557073
C	1.283539	-2.276686	-4.023287
C	2.467088	1.643270	4.299782
C	4.381198	3.651845	-2.686625
C	-0.893706	0.771742	-3.216948
C	-3.873309	4.876376	1.560147
C	-3.381871	-3.959594	-2.393106
C	-3.619875	-0.525420	4.114398
K	0.392564	2.227364	0.608218
H	0.929026	-0.429192	3.301149
H	-0.292972	4.257583	-3.384196
H	-5.031562	2.235905	-1.856562
H	-5.879238	-0.201429	-1.016347
H	-1.357414	5.605938	0.552632
H	-3.856357	2.815548	1.000202
H	-2.862751	1.571907	-3.357320
H	-0.048784	5.882264	-1.532742
H	-0.622654	2.855615	-5.021790
H	-2.316223	3.410116	-5.000551
H	-1.906663	1.780076	-5.589034
H	-3.140267	0.219986	2.183749
H	-3.820161	-1.880287	-2.135486
H	-4.564612	-4.954432	-0.133429
H	-4.601917	-4.982389	2.340917

H	-1.129906	-0.040736	-3.920803
H	-0.967513	0.366403	-2.198350
H	0.152174	1.066422	-3.387683
H	-4.308852	-2.883832	3.611044
H	-1.433630	3.830376	2.584863
H	-1.825661	2.122470	2.174219
H	-2.853664	3.033575	3.282324
H	-5.482754	0.741138	1.469275
H	-5.120824	1.462862	3.054685
H	-6.101051	-0.018812	2.956379
H	-4.541734	4.690047	2.414732
H	-4.487388	5.229303	0.718262
H	-3.194737	5.695095	1.848851
H	-4.444523	-0.993251	4.676080
H	-3.431285	0.455607	4.576559
H	-2.715699	-1.136327	4.248722
H	-6.138173	-3.889245	-2.032469
H	-5.681511	-2.895890	-3.438177
H	-6.309320	-2.124793	-1.961910
H	-2.354988	-3.929752	-2.001460
H	-3.334184	-3.859115	-3.488921
H	-3.803822	-4.954325	-2.179162
H	-0.773562	0.134453	3.428844
H	-1.585494	-2.096454	2.740436
H	0.083575	-2.723574	2.985596
H	2.776621	3.964903	3.309772
H	5.885117	0.728570	0.086931
H	5.650373	-1.868700	-0.676030
H	3.281817	5.072102	-0.806641
H	4.451775	1.704207	-1.816180
H	2.789769	0.373638	2.620812
H	2.791772	5.693270	1.539397
H	5.235089	1.762677	3.845175
H	5.261946	0.532778	2.552568
H	4.704731	0.094554	4.181598
H	1.990304	-1.107098	-2.385388
H	4.703518	-2.791043	1.419366
H	3.048314	-5.999837	0.622591
H	1.943223	-6.454820	-1.541370
H	2.412927	0.782525	4.981884
H	1.431678	1.925965	4.055574
H	2.934525	2.471793	4.856095
H	1.599654	-4.636871	-3.174191
H	1.702256	2.877585	-2.476806
H	2.016672	1.236104	-1.851748
H	2.599654	1.697833	-3.461713
H	4.416608	-1.317554	-2.870212
H	3.529953	-0.791283	-4.319274
H	4.066420	-2.482982	-4.172368
H	4.635717	3.249743	-3.678769
H	5.302681	4.050399	-2.235803
H	3.690630	4.494855	-2.850028
H	1.606998	-3.079871	-4.704522
H	1.120520	-1.378050	-4.637174
H	0.317172	-2.568119	-3.585632
H	4.006673	-5.571853	2.508466
H	5.241572	-4.451840	3.100545
H	5.426462	-5.206656	1.497604
H	2.400657	-2.158233	2.136309
H	3.391698	-2.718552	3.503015
H	2.157504	-3.788457	2.790251
C	-0.579644	-2.988796	0.372490
H	-1.481703	-3.407846	0.824330
C	-0.650185	-2.254124	-0.811789
H	0.347409	-3.516596	0.617583
H	0.228564	-2.129578	-1.438471
H	-1.613044	-1.943660	-1.218307

****

152

K[(C4H8)Al(OBoryl)2)] + ethene

C	-2.579124	2.444779	2.253233
C	-3.268365	2.246096	1.036369
C	-3.507592	3.323785	0.150020
C	-3.064511	4.600450	0.512996
C	-2.385726	4.809143	1.713561
C	-2.145280	3.739309	2.570797
N	-3.658519	0.946093	0.666588
C	-4.976206	0.482129	0.669928
C	-4.987655	-0.804423	0.252237
N	-3.689065	-1.229560	-0.042739
C	-3.435984	-2.535302	-0.536473
C	-3.007464	-2.695491	-1.873671
C	-2.804910	-3.991393	-2.351477
C	-3.038236	-5.102017	-1.545808
C	-3.456504	-4.928654	-0.234466
C	-3.646771	-3.649928	0.300274
C	-2.813602	-1.487230	-2.773491
C	-4.161435	-0.880897	-3.175011
C	-4.064217	-3.502913	1.751391
C	-5.531859	-3.886220	1.951525
C	-4.138681	3.067577	-1.206886
C	-3.069254	2.612927	-2.205641
C	-2.326734	1.300866	3.213463
C	-3.241327	1.420409	4.433011
B	-2.777179	-0.125157	0.209388
O	-1.449597	0.002198	0.049596
Al	0.054692	-0.958134	0.391911
C	-0.087696	-2.114373	1.994477
C	0.511190	-3.429692	1.464150
C	0.181584	-3.649390	-0.022858
C	0.535526	-2.411388	-0.863582
O	1.231772	0.431814	0.384002
B	2.536734	0.569658	0.087497
N	3.681252	-0.300296	0.335298
C	3.792493	-1.468889	1.136620
C	4.210581	-2.689525	0.564808
C	4.376679	-3.796454	1.404805
C	4.111877	-3.717996	2.763834
C	3.681397	-2.514782	3.312900
C	3.528347	-1.374052	2.523890
C	4.495158	-2.845999	-0.917674
C	3.830303	-4.090669	-1.506962
C	3.162401	-0.043383	3.154355
C	2.470010	-0.162465	4.506240
N	3.117730	1.719839	-0.604383
C	2.435065	2.824489	-1.161621
C	2.306497	4.040413	-0.442747
C	1.572144	5.082687	-1.025162
C	0.971241	4.945829	-2.274279
C	1.104158	3.749386	-2.969329
C	1.834378	2.679567	-2.433833
C	2.841925	4.275193	0.962124
C	4.338433	4.034933	1.140399
C	1.922244	1.384535	-3.214037
C	0.551270	0.716403	-3.318604
C	4.479565	1.476119	-0.808442
C	4.809223	0.295884	-0.238284
C	2.546923	1.601391	-4.591392
C	5.999599	-2.884642	-1.198808
C	4.404627	0.843134	3.271672
C	-1.983087	-1.776000	-4.018183
C	-3.170953	-4.311912	2.692031
C	-0.859341	1.186777	3.617229
C	-4.922981	4.255089	-1.751878
C	2.012829	3.525607	2.007364
K	-0.424257	2.433281	-0.129113
H	-1.012114	-2.227310	-3.766169
H	5.117227	2.182840	-1.334289
H	5.790560	-0.164409	-0.164592
H	4.894179	0.990679	2.298756
H	2.579632	0.704384	-2.653423

H	2.657535	0.637486	-5.109534
H	3.540897	2.066641	-4.509516
H	1.922573	2.249739	-5.226994
H	0.158494	0.436105	-2.329393
H	0.623583	-0.208802	-3.905755
H	-0.184319	1.371591	-3.814018
H	0.633041	3.637565	-3.949152
H	1.469066	6.021973	-0.474740
H	0.403661	5.774597	-2.704626
H	4.073649	-1.963315	-1.424109
H	6.520670	-1.994057	-0.820576
H	6.190681	-2.954791	-2.281497
H	6.460605	-3.763078	-0.719305
H	3.955576	-4.109461	-2.600237
H	2.754224	-4.117827	-1.286845
H	4.281459	-5.014416	-1.112602
H	4.702560	-4.746371	0.975849
H	4.231447	-4.598748	3.398993
H	3.469826	-2.462160	4.381538
H	2.447114	0.445002	2.475697
H	5.138267	0.382128	3.952102
H	4.144067	1.834012	3.675982
H	2.114203	0.827120	4.831760
H	1.601909	-0.835727	4.459985
H	1.627206	-2.364820	-1.032087
H	0.058292	-2.443950	-1.855544
H	0.678568	-4.572029	-0.380106
H	-0.900101	-3.843626	-0.118727
H	0.194240	-4.314309	2.049416
H	0.407059	-1.796632	2.925489
H	-1.158330	-2.246856	2.242214
H	-2.462328	-4.141746	-3.375787
H	-0.203350	1.048021	2.744553
H	-2.260214	-0.740266	-2.181197
H	-2.507032	-2.452370	-4.712359
H	-1.788364	-0.840841	-4.563979
H	-0.510762	2.070744	4.175620
H	-4.757256	-1.613200	-3.743117
H	-4.012125	0.000921	-3.818416
H	-4.750283	-0.568534	-2.301243
H	-2.878633	-6.107336	-1.942593
H	-3.618687	-5.803340	0.399569
H	-3.950677	-2.440355	2.016427
H	-3.422900	-4.089515	3.740837
H	-3.304529	-5.395625	2.547990
H	-2.108138	-4.078673	2.539198
H	-5.833406	-3.740552	3.000917
H	-5.697083	-4.945987	1.698548
H	-6.204458	-3.287733	1.320559
H	-5.839892	-1.467102	0.124988
H	-5.807471	1.105283	0.993190
H	-4.299916	1.448893	4.134148
H	-3.099940	0.560635	5.105969
H	-3.027761	2.336807	5.007124
H	-2.595483	0.373133	2.689183
H	-1.615070	3.909860	3.511172
H	-2.045431	5.812635	1.981048
H	1.607829	-3.383909	1.571328
H	3.151675	-0.533879	5.287985
H	-0.713142	0.309970	4.264094
H	-3.242743	5.445196	-0.155271
H	-4.839991	2.227672	-1.088277
H	-5.462130	3.963484	-2.665631
H	-5.662335	4.621153	-1.023730
H	-4.266790	5.098119	-2.021749
H	-3.515631	2.406556	-3.189915
H	-2.304258	3.395935	-2.350968
H	-2.578672	1.683970	-1.877124
H	2.677442	5.350411	1.146209
H	2.394331	3.723587	3.020585
H	0.959507	3.852065	1.994844
H	2.043937	2.438498	1.848075
H	4.663124	4.433140	2.114218
H	4.594724	2.968922	1.111373
H	4.918166	4.546927	0.357493
C	2.678629	-2.132143	-4.140972
H	3.684444	-1.705479	-4.070120
C	2.155525	-2.499341	-5.309343
H	2.125930	-2.243679	-3.203597
H	2.706028	-2.393514	-6.249367
H	1.149516	-2.925896	-5.373722

****

152

TS3 ethene

C	1.935820	2.515008	-2.526261
C	2.748040	2.533614	-1.368527
C	2.893230	3.704170	-0.590285
C	2.223643	4.864637	-0.998527
C	1.424727	4.861976	-2.139789
C	1.283226	3.698158	-2.893631
N	3.375605	1.344786	-0.951439
C	4.740089	1.084635	-1.107099
C	5.016033	-0.137716	-0.597511
N	3.853483	-0.714153	-0.082842
C	3.838299	-1.979393	0.554457
C	3.695925	-2.032340	1.960234
C	3.648603	-3.287668	2.570207
C	3.750517	-4.455545	1.819499
C	3.922513	-4.385099	0.443659
C	3.972495	-3.152542	-0.215929
C	3.652947	-0.751598	2.777880
C	5.031419	-0.085758	2.829486
C	4.238537	-3.096555	-1.709096
C	5.726940	-3.327762	-1.987345
C	3.691226	3.662474	0.701185
C	2.827973	3.121716	1.846018
C	1.803073	1.241823	-3.339651
C	3.040651	1.028946	-4.214570
B	2.746287	0.208059	-0.283088
O	1.455257	0.136782	0.080006
Al	0.068073	-1.058380	-0.011809
C	0.301189	-1.923550	-1.813812
C	-0.280691	-3.340511	-1.804561
C	0.109359	-4.141930	-0.537648
C	0.056404	-3.236778	0.661318
O	-1.357686	0.092217	-0.028622
B	-2.658472	0.159262	0.295394
N	-3.712485	-0.851251	0.254028
C	-3.741185	-2.091963	-0.440649
C	-3.669544	-3.311621	0.267308
C	-3.754162	-4.502038	-0.461455
C	-3.916575	-4.495813	-1.842381
C	-3.992074	-3.288690	-2.524254
C	-3.904025	-2.072592	-1.841579
C	-3.549282	-3.344663	1.780627
C	-2.858351	-4.600781	2.303771
C	-4.023018	-0.772639	-2.610157
C	-3.022891	-0.687541	-3.759240
N	-3.361172	1.358515	0.772720
C	-2.832601	2.633060	1.077794
C	-2.844392	3.672676	0.115070
C	-2.270574	4.904635	0.461222
C	-1.699480	5.119375	1.712110
C	-1.699068	4.093607	2.652020
C	-2.261219	2.845300	2.356551
C	-3.362669	3.528655	-1.308698
C	-4.799500	3.033609	-1.451530
C	-2.270689	1.750369	3.402333
C	-0.969788	1.669718	4.195679
C	-4.700033	1.030950	1.007734
C	-4.908531	-0.260638	0.673501

C	-3.477224	1.907659	4.329828
C	-4.919064	-3.200071	2.451236
C	-5.457470	-0.550096	-3.089211
C	3.118128	-0.931488	4.194033
C	3.396654	-4.076008	-2.522674
C	0.530503	1.172201	-4.173122
C	4.315683	5.000624	1.077488
C	-2.391902	2.718379	-2.169244
K	0.052347	2.358222	0.142581
H	2.133325	-1.418170	4.210043
H	-5.412308	1.764394	1.378265
H	-5.842959	-0.816479	0.676830
H	-6.162782	-0.556724	-2.244718
H	-2.395264	0.799751	2.863640
H	-3.505085	1.089676	5.066151
H	-4.420206	1.885287	3.765245
H	-3.430173	2.861199	4.880689
H	-0.090877	1.610842	3.535586
H	-0.961503	0.762337	4.816501
H	-0.838780	2.533109	4.867448
H	-1.255511	4.262501	3.635613
H	-2.272111	5.709607	-0.278875
H	-1.259261	6.089542	1.955182
H	-2.945338	-2.468348	2.071060
H	-5.403494	-2.247712	2.202469
H	-4.815300	-3.244692	3.546990
H	-5.588636	-4.018364	2.140904
H	-2.691194	-4.510943	3.388092
H	-1.883965	-4.764904	1.821561
H	-3.481029	-5.497023	2.154593
H	-3.689462	-5.456899	0.061699
H	-3.977415	-5.439846	-2.388962
H	-4.116777	-3.287883	-3.609849
H	-3.786698	0.039920	-1.908884
H	-5.769202	-1.336854	-3.795046
H	-5.551003	0.420202	-3.602682
H	-3.082208	0.295966	-4.251449
H	-1.995549	-0.830210	-3.395487
H	-0.912397	-2.766313	0.830116
H	0.994012	-2.734121	0.896223
H	-0.568629	-5.006936	-0.433357
H	1.128304	-4.550564	-0.635831
H	-0.010429	-3.926706	-2.704451
H	-0.114417	-1.345502	-2.657099
H	1.394627	-1.964698	-1.994929
H	3.531381	-3.358271	3.652557
H	-0.369890	1.318678	-3.559254
H	2.968933	-0.067242	2.249796
H	3.809043	-1.522379	4.816644
H	3.011426	0.051909	4.677504
H	0.521161	1.915824	-4.986585
H	5.758897	-0.747426	3.326467
H	4.983127	0.852566	3.405584
H	5.414206	0.153689	1.828370
H	3.699711	-5.427776	2.315151
H	4.014415	-5.306959	-0.135082
H	3.983681	-2.079807	-2.046243
H	3.602592	-3.944899	-3.596383
H	3.629235	-5.123582	-2.274346
H	2.322397	-3.917994	-2.364479
H	5.944848	-3.217661	-3.061458
H	6.024892	-4.344554	-1.684347
H	6.364929	-2.621637	-1.437146
H	5.973544	-0.650285	-0.551725
H	5.410911	1.793165	-1.588640
H	3.959337	0.987660	-3.612874
H	2.957215	0.079162	-4.765128
H	3.144485	1.842580	-4.950658
H	1.760987	0.410024	-2.619765
H	0.654878	3.710436	-3.785886
H	0.908643	5.775399	-2.445857
H	-1.380827	-3.268235	-1.822054
H	-3.216899	-1.450590	-4.529486
H	0.448894	0.179502	-4.638993
H	2.323366	5.782138	-0.415280
H	4.508501	2.939935	0.553063
H	4.985437	4.874915	1.941397
H	4.905821	5.419878	0.248769
H	3.557278	5.746495	1.364976
H	3.410673	3.052501	2.777108
H	1.971698	3.790188	2.046087
H	2.455626	2.109283	1.626621
H	-3.351117	4.555623	-1.711322
H	-2.764078	2.635954	-3.201644
H	-1.407011	3.209150	-2.224440
H	-2.252594	1.696661	-1.785584
H	-5.137064	3.186431	-2.488489
H	-4.904004	1.966701	-1.217622
H	-5.481335	3.591037	-0.791669
C	-0.074421	-1.237448	2.419942
H	0.789378	-0.582495	2.570278
C	0.026089	-2.588289	2.780029
H	-1.047619	-0.750162	2.538375
H	0.981179	-3.019787	3.087423
H	-0.844881	-3.146454	3.124888

****

152

K[(C6H12)Al(OBoryl)2]

C	2.063596	-2.431963	2.471312
C	2.847742	-2.452611	1.293936
C	3.038182	-3.647312	0.561554
C	2.455371	-4.826189	1.043264
C	1.689763	-4.821218	2.206669
C	1.493680	-3.633646	2.909002
N	3.400116	-1.250254	0.814299
C	4.771449	-0.981358	0.835636
C	4.990613	0.231080	0.280731
N	3.783129	0.798125	-0.132204
C	3.765316	2.072908	-0.752288
C	3.449848	2.167696	-2.125269
C	3.516110	3.423182	-2.732248
C	3.882549	4.554667	-2.009358
C	4.176532	4.446769	-0.657333
C	4.117867	3.212727	-0.001830
C	3.090141	0.924139	-2.918916
C	4.338068	0.089684	-3.220005
C	4.432861	3.135878	1.480655
C	5.923264	3.355652	1.745723
C	3.772972	-3.619642	-0.767559
C	2.835004	-3.142916	-1.881663
C	1.864340	-1.134233	3.229849
C	3.106066	-0.795956	4.057306
B	2.702356	-0.127007	0.180189
O	1.384063	-0.079191	-0.071023
Al	-0.060497	1.039065	0.097577
C	0.296381	2.031197	1.791184
C	-0.273026	3.446540	1.979059
C	0.419949	4.165050	-0.395386
C	0.026104	3.487725	-1.709257
O	-1.363056	-0.253669	0.211061
B	-2.662952	-0.396727	-0.098269
N	-3.794401	0.515923	0.020860
C	-3.890373	1.803333	0.605995
C	-4.287796	2.904102	-0.182165
C	-4.446351	4.145371	0.442613
C	-4.209102	4.302405	1.800596
C	-3.809379	3.209726	2.563646
C	-3.646942	1.946890	1.991391
C	-4.562192	2.784379	-1.670762
C	-3.782106	3.811834	-2.489513
C	-3.277980	0.741858	2.839853

C	-2.645816	1.097774	4.179923
N	-3.267832	-1.605085	-0.671026
C	-2.608403	-2.756707	-1.149363
C	-2.566561	-3.942086	-0.375439
C	-1.875474	-5.046601	-0.893395
C	-1.233863	-4.992112	-2.128126
C	-1.269175	-3.816395	-2.871644
C	-1.947090	-2.685484	-2.399246
C	-3.154543	-4.074178	1.022474
C	-4.656368	-3.818181	1.128084
C	-1.977699	-1.414276	-3.223593
C	-0.620221	-1.066689	-3.826279
C	-4.620163	-1.357097	-0.923765
C	-4.929562	-0.114298	-0.496747
C	-3.064651	-1.500548	-4.296101
C	-6.059005	2.898581	-1.968704
C	-4.495965	-0.161135	3.055316
C	2.327172	1.217672	-4.204884
C	3.592520	4.116150	2.297424
C	0.606465	-1.113376	4.088459
C	4.422098	-4.947702	-1.137401
C	-2.371203	-3.249377	2.046426
K	0.152655	-2.405279	-0.034232
H	1.434149	1.831612	-4.016275
H	-5.267168	-2.106723	-1.372921
H	-5.899628	0.375139	-0.489153
H	-4.937592	-0.489374	2.103928
H	-2.251350	-0.593595	-2.544545
H	-3.119189	-0.559546	-4.865054
H	-4.051751	-1.681160	-3.846212
H	-2.857687	-2.317314	-5.006800
H	0.160336	-0.988444	-3.053420
H	-0.671387	-0.090565	-4.329608
H	-0.290592	-1.803745	-4.575884
H	-0.759205	-3.772752	-3.836572
H	-1.837400	-5.966186	-0.302916
H	-0.703719	-5.868318	-2.509413
H	-4.225922	1.786016	-1.989363
H	-6.651053	2.156015	-1.414317
H	-6.254800	2.755281	-3.043161
H	-6.438493	3.894414	-1.688681
H	-3.939779	3.639831	-3.565926
H	-2.704210	3.749808	-2.289783
H	-4.108772	4.840738	-2.271600
H	-4.751035	5.008553	-0.153871
H	-4.329773	5.281659	2.269643
H	-3.624744	3.345056	3.630090
H	-2.521633	0.175757	2.272894
H	-5.273892	0.373785	3.623315
H	-4.217387	-1.059719	3.628702
H	-2.302309	0.181893	4.684485
H	-1.777481	1.761546	4.058124
H	-0.697865	4.133592	-2.242808
H	0.919359	3.468476	-2.351721
H	0.748170	5.198323	-0.597898
H	1.320002	3.652503	-0.015242
H	0.443873	4.088285	2.526092
H	0.042689	1.404006	2.664275
H	1.404142	2.070685	1.802116
H	3.275097	3.523661	-3.791370
H	-0.294384	-1.348123	3.500081
H	2.422442	0.327030	-2.276720
H	2.953937	1.739392	-4.945712
H	1.999165	0.275277	-4.669097
H	0.662545	-1.819249	4.932832
H	5.046367	0.665929	-3.836714
H	4.069624	-0.820969	-3.779272
H	4.858385	-0.215936	-2.301076
H	3.927724	5.527471	-2.504614
H	4.446916	5.341440	-0.091316
H	4.174787	2.120582	1.818290
H	3.776319	3.972000	3.373737
H	3.839498	5.162832	2.059654
H	2.518850	3.974176	2.114727
H	6.147012	3.254418	2.819484
H	6.233953	4.365278	1.431986
H	6.548431	2.633807	1.200345
H	5.935012	0.749430	0.134795
H	5.487337	-1.674288	1.273010
H	4.004062	-0.732520	3.426201
H	2.976357	0.176408	4.557134
H	3.281362	-1.559182	4.832669
H	1.748627	-0.341545	2.475704
H	0.887317	-3.641496	3.816253
H	1.240562	-5.749418	2.568475
H	-1.165348	3.405215	2.622590
H	-3.363138	1.588788	4.856947
H	0.465287	-0.109260	4.513344
H	2.593012	-5.760721	0.496318
H	4.572788	-2.867943	-0.685044
H	5.045938	-4.824687	-2.035328
H	5.064333	-5.325254	-0.327530
H	3.675857	-5.724697	-1.368849
H	3.362710	-3.105524	-2.846371
H	1.979585	-3.830392	-2.002851
H	2.460008	-2.126585	-1.686104
H	-3.000902	-5.133390	1.289826
H	-2.802481	-3.372417	3.051494
H	-1.320909	-3.577469	2.110562
H	-2.394497	-2.176346	1.806356
H	-5.021359	-4.166663	2.106676
H	-4.901446	-2.751953	1.038898
H	-5.210363	-4.363546	0.349069
C	-0.677532	4.146100	0.680151
H	-1.015995	5.171181	0.906148
H	-1.571434	3.632326	0.288054
C	-0.522264	2.063080	-1.544333
H	-1.628176	2.088269	-1.490038
H	-0.311126	1.467010	-2.449637

****

143

TS1 propene

C	-3.439242	-1.974948	1.947961
C	-3.927494	-1.593063	0.678521
C	-4.576989	-2.518302	-0.165051
C	-4.774496	-3.820907	0.305980
C	-4.301946	-4.211443	1.551205
C	-3.626439	-3.297553	2.354951
N	-3.783595	-0.248801	0.255547
C	-4.898404	0.555448	0.007335
C	-4.509814	1.758165	-0.472224
N	-3.114831	1.782950	-0.562942
C	-2.387150	2.874745	-1.070499
C	-1.720173	2.742796	-2.312350
C	-0.975193	3.832324	-2.780763
C	-0.887811	5.014660	-2.047270
C	-1.533356	5.123309	-0.817889
C	-2.281428	4.057051	-0.303270
C	-1.801793	1.440742	-3.086837
C	-3.164730	1.286105	-3.764822
C	-2.878976	4.107573	1.091480
C	-1.968376	3.361063	2.072101
C	-5.041121	-2.153613	-1.564179
C	-6.567179	-2.127251	-1.658884
C	-2.795941	-0.949924	2.865331
C	-3.857759	-0.032982	3.478290
B	-2.585974	0.510093	-0.073199
O	-1.280804	0.229319	0.058089
Al	-0.101115	-1.166218	0.145455
C	-0.285478	-2.831986	-1.081825
C	-0.666814	-1.897206	-2.080727

O	1.424183	-0.182348	-0.121481
B	2.733394	-0.333330	0.154947
N	3.613887	0.717193	0.659933
C	3.238912	2.013287	1.060342
C	2.477028	2.174998	2.242310
C	2.071132	3.468176	2.594901
C	2.406146	4.568013	1.806883
C	3.148643	4.393250	0.641625
C	3.568914	3.117966	0.243737
C	2.117455	0.969282	3.091025
C	3.324244	0.486708	3.898661
C	4.274558	2.894907	-1.081633
C	3.261102	2.466003	-2.148020
N	3.620072	-1.471407	0.008786
C	4.909790	-1.088565	0.376683
C	4.914179	0.210025	0.759231
C	3.298272	-2.793006	-0.390008
C	3.472019	-3.159049	-1.739051
C	3.120411	-4.455984	-2.123238
C	2.616217	-5.360940	-1.194555
C	2.461529	-4.986478	0.135887
C	2.798919	-3.699874	0.566704
C	3.976475	-2.141456	-2.744744
C	4.784253	-2.757910	-3.881432
C	2.675663	-3.304093	2.025275
C	3.975421	-3.608246	2.773856
C	1.473874	-3.927671	2.724038
C	2.814709	-1.306081	-3.286794
C	0.912628	1.191877	3.995522
C	5.088936	4.092981	-1.553742
C	-0.668271	1.246704	-4.086224
C	-3.172812	5.517461	1.587557
C	-4.451439	-3.084303	-2.624740
C	-1.919011	-1.565242	3.947207
K	0.453401	2.258134	-0.202038
H	0.767606	-3.137670	-1.130646
H	-0.455318	3.756282	-3.737286
H	-5.123009	2.602549	-0.780157
H	-5.905932	0.198786	0.206792
H	-1.443314	6.047947	-0.245003
H	-3.831980	3.557445	1.061099
H	-1.704120	0.628784	-2.348776
H	-0.308047	5.855723	-2.435571
H	-3.314499	2.067266	-4.527633
H	-3.987354	1.351736	-3.038574
H	-3.231948	0.305973	-4.262042
H	-2.134894	-0.331716	2.237447
H	-4.672791	-1.139559	-1.780134
H	-5.282820	-4.548913	-0.331058
H	-4.447568	-5.238011	1.895786
H	-0.725292	0.235168	-4.513557
H	0.321745	1.342577	-3.611909
H	-0.718593	1.960725	-4.924243
H	-3.246651	-3.620520	3.324947
H	-0.985176	3.856320	2.149193
H	-1.818931	2.314910	1.764734
H	-2.408122	3.341716	3.080994
H	-4.456723	0.469078	2.704845
H	-3.384640	0.744777	4.099768
H	-4.543895	-0.607681	4.121120
H	-3.713241	5.474646	2.544963
H	-3.793270	6.079806	0.873459
H	-2.250989	6.094635	1.764745
H	-2.502257	-2.162639	4.666243
H	-1.422542	-0.768685	4.521142
H	-1.138075	-2.203209	3.505775
H	-6.992271	-3.125280	-1.465280
H	-6.890553	-1.815130	-2.664511
H	-7.010917	-1.433727	-0.929437
H	-3.353033	-3.104107	-2.584123
H	-4.748726	-2.749040	-3.631176
H	-4.810248	-4.118775	-2.504470
C	-1.192312	-3.939390	-0.613572
H	-1.721085	-1.659054	-2.248998
H	0.065395	-1.468229	-2.765554
H	1.484491	3.619513	3.502465
H	5.742866	0.815823	1.120001
H	5.741738	-1.788553	0.334252
H	3.396308	5.261437	0.028019
H	4.967329	2.050615	-0.943735
H	1.842853	0.160458	2.393309
H	2.083586	5.569188	2.103325
H	3.649401	1.259039	4.614237
H	4.173631	0.236888	3.247743
H	3.062282	-0.418192	4.468777
H	4.644220	-1.456300	-2.198583
H	2.528437	-2.212521	2.043669
H	2.060850	-5.705885	0.852250
H	2.336577	-6.368236	-1.512433
H	0.647010	0.246823	4.489979
H	0.028354	1.526047	3.430899
H	1.115066	1.929515	4.788642
H	3.234616	-4.762625	-3.164550
H	2.516356	3.261696	-2.325969
H	2.740716	1.540108	-1.859496
H	3.761023	2.265985	-3.108024
H	5.599815	-3.392730	-3.502952
H	5.228968	-1.966065	-4.503776
H	4.157048	-3.373392	-4.545611
H	5.674344	3.823371	-2.445497
H	5.790385	4.438200	-0.779284
H	4.448793	4.944351	-1.835664
H	2.114004	-1.939423	-3.854053
H	3.180792	-0.513388	-3.959291
H	2.246914	-0.836693	-2.470945
H	4.186634	-4.689903	2.767455
H	3.904382	-3.282314	3.824001
H	4.833521	-3.093040	2.318580
H	0.547753	-3.690069	2.176915
H	1.379198	-3.520617	3.743101
H	1.566919	-5.021305	2.820420
H	-1.281349	-4.703288	-1.410207
H	-0.798037	-4.421033	0.292293
H	-2.203599	-3.580147	-0.387757

****

143

K[(C2H3Me)Al(OBoryl)2]

K	0.182707	-2.245532	0.050377
Al	-0.054286	1.156167	-0.431136
O	1.361307	0.084896	-0.115347
N	3.534325	-1.073731	-0.437231
N	3.671206	1.058383	0.379056
C	3.071867	-2.315017	-0.909193
C	2.491677	-2.393515	-2.195448
C	3.121006	-3.448443	-0.059948
C	1.953820	-3.620214	-2.609328
H	1.502949	-3.697250	-3.601985
C	4.875677	-0.756537	-0.204312
H	5.681443	-1.451878	-0.429845
C	3.474894	2.406604	0.771926
C	4.949706	0.506963	0.273839
H	5.832892	1.079363	0.549237
C	2.584222	-4.654481	-0.523203
H	2.613140	-5.539723	0.114659
C	3.660410	-3.319582	1.354864
H	4.495928	-2.604038	1.322816
C	2.479523	-1.205033	-3.136331
H	2.818242	-0.331466	-2.562053
C	1.996249	-4.740540	-1.785281
H	1.575866	-5.689276	-2.127899
C	3.925394	3.440413	-0.069780

C	2.809635	2.683617	1.984320
C	3.482870	-1.425737	-4.269644
H	3.200935	-2.288836	-4.894568
H	4.493629	-1.611263	-3.876063
H	3.527528	-0.539372	-4.921084
C	2.309262	1.551059	2.860663
H	1.896572	0.788843	2.180975
C	4.522066	3.162539	-1.436069
H	4.525454	2.072589	-1.582054
C	3.745910	4.763001	0.348699
H	4.086175	5.579264	-0.293491
C	3.109557	5.052864	1.547207
H	2.961347	6.091456	1.852150
C	1.086096	-0.884414	-3.670121
H	1.125370	-0.003785	-4.327983
H	0.390672	-0.624328	-2.858327
H	0.660157	-1.717621	-4.252374
C	2.635410	4.018825	2.349681
H	2.112488	4.261626	3.275493
B	2.705939	0.074095	-0.072430
C	2.601213	-2.711396	2.279761
H	1.717680	-3.369562	2.355572
H	2.286352	-1.714791	1.934654
H	2.998676	-2.581856	3.297662
C	3.462111	0.912003	3.637720
H	4.248976	0.545128	2.962505
H	3.103383	0.060099	4.238314
H	3.917665	1.642183	4.325713
C	4.192544	-4.626436	1.929801
H	4.688096	-4.439299	2.894146
H	4.925435	-5.097714	1.257801
H	3.387649	-5.354983	2.118645
C	1.178790	1.962577	3.794777
H	1.512719	2.684446	4.556959
H	0.798895	1.082171	4.333918
H	0.343590	2.410370	3.234703
C	5.968570	3.643651	-1.535840
H	6.038967	4.735275	-1.403618
H	6.393387	3.400306	-2.522548
H	6.603038	3.175797	-0.767536
C	3.651383	3.761568	-2.541087
H	2.613220	3.407364	-2.461769
H	4.039729	3.482026	-3.533566
H	3.631371	4.861766	-2.489524
C	-0.529063	4.339314	-0.047623
H	-1.621189	4.234585	-0.130987
H	-0.239625	5.191310	-0.694779
H	-0.302353	4.631827	0.992505
C	0.186420	3.070032	-0.432446
H	1.274237	3.201419	-0.310120
C	-0.094236	2.456156	-1.858142
H	0.606967	2.721250	-2.667773
H	-1.116756	2.714523	-2.174487
O	-1.361229	-0.010729	-0.023629
N	-3.288667	-1.419701	0.711032
N	-3.808186	0.700261	0.036918
C	-2.682743	-2.673002	0.930237
C	-2.126928	-2.965900	2.195007
C	-2.616922	-3.603498	-0.131658
C	-1.458636	-4.185536	2.363713
H	-1.014688	-4.427954	3.331714
C	-4.670396	-1.226367	0.821233
H	-5.330829	-2.011620	1.182546
C	-3.873172	2.018744	-0.483960
C	-4.969658	0.031720	0.427004
H	-5.940851	0.520761	0.385595
C	-1.942911	-4.812484	0.082308
H	-1.869848	-5.540950	-0.728036
C	-3.196278	-3.259167	-1.491104
H	-3.981817	-2.506848	-1.323066
C	-2.215907	-1.951312	3.316519
H	-3.100665	-1.331321	3.105072
C	-1.360566	-5.099019	1.315748
H	-0.835152	-6.045719	1.463916
C	-4.106477	3.088600	0.402200
C	-3.755782	2.215221	-1.874281
C	-2.404750	-2.589498	4.688544
H	-1.507498	-3.137689	5.017867
H	-3.253313	-3.290159	4.698004
H	-2.598026	-1.811182	5.441974
C	-3.511549	1.035259	-2.798966
H	-2.794433	0.380008	-2.279925
C	-4.120543	2.844197	1.900288
H	-4.595577	1.864058	2.065380
C	-4.260451	4.367939	-0.138102
H	-4.443747	5.216456	0.522975
C	-4.160154	4.579470	-1.509130
H	-4.270935	5.588462	-1.913249
C	-1.001830	-1.019942	3.302963
H	-1.099038	-0.248094	4.080576
H	-0.899413	-0.499062	2.339651
H	-0.071450	-1.578127	3.502678
C	-3.897593	3.515929	-2.365545
H	-3.800658	3.701965	-3.436194
B	-2.677205	-0.189023	0.209534
C	-2.130712	-2.608069	-2.377502
H	-1.281393	-3.292015	-2.548726
H	-1.752796	-1.673853	-1.936174
H	-2.545526	-2.346978	-3.362854
C	-4.798968	0.238690	-3.027377
H	-5.234270	-0.120786	-2.084385
H	-4.603489	-0.637679	-3.666890
H	-5.553039	0.861714	-3.534715
C	-3.836744	-4.453852	-2.189373
H	-4.355590	-4.122610	-3.101495
H	-4.573088	-4.953324	-1.542049
H	-3.091383	-5.204428	-2.497679
C	-2.878126	1.412816	-4.132547
H	-3.569617	1.989654	-4.767710
H	-2.614456	0.500395	-4.689872
H	-1.958657	1.998108	-3.991926
C	-4.924800	3.874389	2.684064
H	-4.451337	4.868687	2.660282
H	-4.992291	3.576618	3.741689
H	-5.948802	3.977178	2.293290
C	-2.689205	2.747290	2.437214
H	-2.090093	2.020589	1.870238
H	-2.689579	2.446160	3.497290
H	-2.175383	3.716640	2.353912

****

152

TS2 propene (to rac-3,4-isomer)

C	-4.014951	-2.935870	-0.694455
C	-3.920378	-1.981439	0.334875
C	-3.902145	-2.360091	1.693904
C	-4.027907	-3.717372	1.998840
C	-4.157602	-4.671280	0.992667
C	-4.141497	-4.282694	-0.340272
N	-3.873143	-0.606055	-0.000063
C	-5.027206	0.059499	-0.418125
C	-4.749874	1.360484	-0.659403
N	-3.395517	1.590731	-0.402355
C	-2.771239	2.835597	-0.606271
C	-2.459300	3.643723	0.510945
C	-1.795397	4.856730	0.289593
C	-1.447866	5.256229	-0.999599
C	-1.767580	4.452467	-2.091711
C	-2.434023	3.233626	-1.919178
C	-2.837006	3.182740	1.906789
C	-4.305865	3.496346	2.199118
C	-2.753177	2.318050	-3.086103

C	-2.945154	3.056175	-4.405612
C	-3.747933	-1.310718	2.781773
C	-3.263982	-1.877594	4.111660
C	-3.956555	-2.530496	-2.154335
C	-2.855617	-3.275150	-2.908628
B	-2.765543	0.334839	0.008450
O	-1.463778	0.188714	0.302705
Al	-0.116810	-0.966834	0.641697
C	0.278482	-2.920628	1.194344
C	-0.277987	-3.940049	2.145265
O	1.368339	-0.083252	0.128900
B	2.695635	-0.159083	-0.064345
N	3.644167	0.942041	0.131278
C	3.331831	2.294338	0.390901
C	3.076797	3.160418	-0.695552
C	2.675291	4.476332	-0.428161
C	2.534283	4.928069	0.880412
C	2.820777	4.075099	1.945643
C	3.235161	2.755950	1.725922
C	3.171790	2.669445	-2.128036
C	3.952711	3.628814	-3.021919
C	3.623014	1.850084	2.876488
C	5.146564	1.817906	3.030389
C	4.912901	0.520882	-0.282210
C	4.842158	-0.767226	-0.686169
N	3.533432	-1.239184	-0.568312
C	3.173915	-2.522679	-1.058798
C	3.545889	-3.677750	-0.342699
C	3.225342	-4.926294	-0.884646
C	2.546498	-5.031873	-2.091629
C	2.168772	-3.882634	-2.777890
C	2.470527	-2.614482	-2.278352
C	4.276098	-3.591475	0.983993
C	3.689350	-4.529812	2.038242
C	2.093378	-1.363197	-3.046563
C	0.801388	-1.499942	-3.842243
C	1.780283	2.387056	-2.695021
C	2.961745	2.206435	4.202426
C	3.254738	-0.915469	-3.936573
C	5.773228	-3.860379	0.815869
C	-0.024176	0.282158	2.768350
C	0.300251	-1.061100	2.952493
C	1.712080	-1.451303	3.285113
C	-0.390637	-2.730603	-0.157466
C	-1.934890	3.735048	3.005522
C	-1.695473	1.219781	-3.225506
C	-5.036867	-0.511581	2.990903
C	-5.313161	-2.704491	-2.837091
K	0.201870	2.295868	0.297135
H	2.460667	5.153653	-1.258342
H	5.638969	-1.402614	-1.064816
H	3.303280	0.835389	2.597955
H	2.210264	5.953682	1.073835
H	2.725567	4.445093	2.968051
H	3.717539	1.714920	-2.111836
H	1.620843	-3.975038	-3.717231
H	5.776136	1.182234	-0.252959
H	1.918738	-0.577369	-2.298407
H	4.165445	-0.733549	-3.346820
H	3.004289	0.014324	-4.472776
H	3.486782	-1.685908	-4.689402
H	3.330346	3.164377	4.603184
H	3.192531	1.434232	4.951208
H	1.866039	2.263676	4.120108
H	0.893761	-2.220052	-4.670756
H	0.531583	-0.530949	-4.289377
H	-0.028330	-1.818506	-3.194432
H	3.503341	-5.833117	-0.342897
H	4.155702	-2.558603	1.347068
H	5.638098	1.465926	2.113425
H	5.434309	1.137991	3.847217
H	5.536089	2.820875	3.268299
H	1.284648	1.569176	-2.151014
H	1.842270	2.069686	-3.747031
H	1.135905	3.281871	-2.656897
H	2.298739	-6.016228	-2.495966
H	5.944224	-4.879796	0.433803
H	6.296719	-3.769212	1.780896
H	6.241055	-3.158396	0.111837
H	3.427213	4.586695	-3.163738
H	4.096120	3.185172	-4.018982
H	4.945544	3.848051	-2.601565
H	0.775963	1.023064	2.689905
H	-1.041938	0.644520	2.918488
H	2.599304	-4.417055	2.120025
H	4.133261	-4.320751	3.023936
H	3.899656	-5.585671	1.805851
H	-1.542600	5.498591	1.135902
H	-5.981010	-0.458574	-0.488441
H	-3.703577	1.817828	-2.842940
H	-0.927024	6.204421	-1.153885
H	-1.493855	4.780392	-3.096417
H	-2.734967	2.085631	1.911046
H	-4.019515	-4.041000	3.040695
H	-5.418516	2.156589	-0.980428
H	-2.977104	-0.607309	2.423860
H	-5.364914	-0.009640	2.070469
H	-4.889361	0.260684	3.763220
H	-5.849575	-1.174617	3.328581
H	-0.734629	1.647130	-3.550343
H	-2.003018	0.479652	-3.979987
H	-1.529211	0.679493	-2.281982
H	-4.044718	-2.477891	4.605695
H	-3.001014	-1.057937	4.797333
H	-2.376213	-2.514957	3.990364
H	-4.214643	-5.039997	-1.124750
H	-3.702934	-1.460192	-2.178813
H	-3.684098	3.867278	-4.318222
H	-3.298665	2.357276	-5.178407
H	-2.003121	3.491863	-4.775941
H	-0.865195	3.549051	2.813643
H	-2.176209	3.254414	3.964912
H	-2.064403	4.820152	3.143437
H	-4.253711	-5.727784	1.253981
H	-5.627978	-3.760665	-2.831220
H	-5.266921	-2.374633	-3.887209
H	-6.097249	-2.120320	-2.332821
H	-4.484680	4.583326	2.177905
H	-4.589808	3.121204	3.194593
H	-4.967626	3.022088	1.461135
H	-1.885103	-3.160340	-2.406149
H	-2.764068	-2.884682	-3.934737
H	-3.070476	-4.353001	-2.985944
H	0.151896	-3.153996	-1.014982
H	-1.432667	-3.079297	-0.143850
H	1.370656	-2.993697	1.087763
H	-0.078179	-4.970697	1.792666
H	0.138833	-3.864917	3.166291
H	-1.373089	-3.848700	2.222890
H	-0.475175	-1.708058	3.372641
H	1.977951	-1.054000	4.281010
H	1.858122	-2.537275	3.297649
H	2.423401	-1.025387	2.561766

****

152

rac-3,4-isomer K[(C4H6Me2)Al(OBoryl)2]

C	-3.767046	-4.467550	1.829861
C	-3.426889	-3.351767	2.589350
C	-3.429805	-2.070734	2.034440
C	-3.768409	-1.936639	0.668829
C	-4.115815	-3.056919	-0.113149

C	-4.113109	-4.317097	0.494447
N	-3.787556	-0.643977	0.085741
B	-2.691999	0.271588	-0.195345
N	-3.366851	1.429040	-0.783951
C	-4.742081	1.184920	-0.824711
C	-4.984029	-0.042741	-0.312691
C	-3.117808	-0.844311	2.875953
C	-2.446840	-1.162972	4.206518
C	-4.522271	-2.927674	-1.569976
C	-3.745527	-3.867401	-2.489449
C	-2.771483	2.621264	-1.234642
C	-2.860364	3.787654	-0.436361
C	-2.212408	4.948081	-0.873112
C	-1.481852	4.957564	-2.061878
C	-1.399893	3.804335	-2.836171
C	-2.043698	2.621991	-2.444566
C	-3.563720	3.736572	0.908358
C	-4.154147	5.073294	1.340517
C	-1.950419	1.384498	-3.312861
C	-2.625424	1.615311	-4.664650
C	-0.508561	0.908520	-3.478151
C	-2.617497	3.185137	1.979849
C	-4.377912	-0.009363	3.124686
C	-6.028355	-3.145843	-1.733592
O	-1.370830	0.193476	0.037704
Al	0.000207	-1.004659	-0.001303
C	-0.131151	-2.341691	-1.451676
C	0.302794	-3.624568	-0.714634
C	-0.302473	-3.624936	0.710372
C	0.132294	-2.342711	1.448020
O	1.370163	0.195051	-0.038033
B	2.691377	0.272882	0.195116
N	3.786752	-0.643611	-0.084123
C	4.983278	-0.042439	0.314267
C	4.741640	1.185923	0.824682
N	3.366555	1.430623	0.782815
C	2.772061	2.623791	1.232229
C	2.858305	3.788107	0.430718
C	2.211224	4.949462	0.866302
C	1.484190	4.961811	2.057201
C	1.404950	3.810595	2.834811
C	2.048129	2.627485	2.444431
C	3.557776	3.733558	-0.915804
C	4.148050	5.068857	-1.352528
C	1.957011	1.391820	3.315590
C	2.627113	1.627768	4.668887
C	3.768088	-1.936709	-0.666322
C	3.431891	-2.071661	-2.032452
C	3.430079	-3.353029	-2.586603
C	3.768937	-4.468337	-1.825836
C	4.112610	-4.317063	-0.489899
C	4.114253	-3.056506	0.116923
C	3.121079	-0.845805	-2.875243
C	2.452706	-1.165490	-4.206867
C	4.518790	-2.926258	1.574202
C	3.742345	-3.866652	2.493267
C	0.516154	0.911392	3.477602
C	2.607612	3.180668	-1.983018
C	4.381257	-0.010452	-3.122260
C	6.024944	-3.142669	1.739672
K	-0.002818	2.462925	0.006197
H	2.265896	5.857757	0.263126
H	5.937558	-0.547823	0.189399
H	2.514908	0.595657	2.802204
H	0.981516	5.876913	2.380131
H	0.838005	3.828092	3.769334
H	4.385727	3.014085	-0.823333
H	3.158685	-3.488398	-3.634034
H	5.442793	1.906761	1.239983
H	2.411078	-0.236702	-2.291498
H	4.846254	0.328747	-2.186554

H	4.139951	0.880398	-3.724137
H	5.127343	-0.598822	-3.680149
H	-0.116121	1.666495	3.973058
H	0.483370	-0.002055	4.088894
H	0.059787	0.656000	2.509884
H	3.135333	-1.698994	-4.887612
H	2.158141	-0.232187	-4.710072
H	1.549280	-1.778926	-4.078323
H	4.373341	-5.197322	0.102457
H	4.288199	-1.896966	1.887916
H	3.674128	1.942800	4.545550
H	2.617124	0.703925	5.267416
H	2.107005	2.406348	5.250180
H	2.264753	2.165125	-1.733447
H	3.111824	3.115656	-2.958873
H	1.729471	3.837447	-2.110759
H	3.755571	-5.462007	-2.279531
H	6.306090	-4.170025	1.457304
H	6.329646	-2.986592	2.786674
H	6.611255	-2.457947	1.109630
H	3.367991	5.809235	-1.592420
H	4.752320	4.934080	-2.262092
H	4.796634	5.499802	-0.575015
H	1.190941	-2.441041	1.758688
H	-0.450359	-2.205049	2.371713
H	2.659033	-3.707022	2.408713
H	4.032093	-3.697546	3.542302
H	3.945439	-4.924869	2.265185
H	-2.269179	5.857955	-0.272521
H	-5.938473	-0.547614	-0.186918
H	-2.504152	0.587544	-2.796253
H	-0.978602	5.872015	-2.385746
H	-0.830300	3.819560	-3.769110
H	-4.392053	3.017643	0.815187
H	-3.153614	-3.486483	3.636377
H	-5.443117	1.905303	-1.241002
H	-2.409042	-0.235329	2.290574
H	-4.844585	0.329152	2.189555
H	-4.136016	0.881884	3.725734
H	-5.122825	-0.597791	3.684078
H	0.119276	1.663453	-3.979432
H	-0.475058	-0.007571	-4.085396
H	-0.047566	0.659346	-2.511016
H	-3.127906	-1.696473	4.888825
H	-2.151827	-0.229250	4.708699
H	-1.543320	-1.775977	4.076573
H	-4.374775	-5.197743	-0.096931
H	-4.293278	-1.898269	-1.884426
H	-3.673413	1.926086	-4.538796
H	-2.613209	0.690558	-5.261742
H	-2.110254	2.395289	-5.248480
H	-2.275356	2.168418	1.733995
H	-3.124882	3.123152	2.954247
H	-1.739008	3.841245	2.108952
H	-3.752811	-5.460952	2.284116
H	-6.308112	-4.173388	-1.450533
H	-6.334493	-2.990449	-2.780275
H	-6.614608	-2.461511	-1.103035
H	-3.374185	5.813522	1.581182
H	-4.761277	4.940960	2.248534
H	-4.800006	5.503140	0.560138
H	-2.662380	-3.705871	-2.406300
H	-4.036831	-3.699449	-3.538237
H	-3.946502	-4.925806	-2.260385
H	0.452369	-2.203540	-2.374736
H	-1.189549	-2.439487	-1.763479
C	0.004741	-4.895002	-1.502682
H	1.400257	-3.574584	-0.582751
H	0.387101	-4.807149	-2.532078
H	0.467603	-5.789004	-1.055251
H	-1.081727	-5.075956	-1.563240

C	-0.004501	-4.895994	1.497475
H	-1.399977	-3.574530	0.578790
H	-0.385905	-4.808489	2.527250
H	-0.468259	-5.789492	1.049958
H	1.081933	-5.077566	1.556946

****

152

TS2 propene (to meso-3,4-isomer)

C	3.716651	-3.549529	-0.009762
C	3.417851	-2.462614	0.836228
C	2.851996	-2.659336	2.109884
C	2.582720	-3.966218	2.523386
C	2.869568	-5.049188	1.701239
C	3.433663	-4.839697	0.447239
N	3.698267	-1.139890	0.407090
C	4.959335	-0.571561	0.584993
C	4.932021	0.735611	0.239865
N	3.640326	1.070223	-0.181834
C	3.247369	2.388998	-0.488984
C	3.005685	2.750113	-1.836328
C	2.517995	4.035459	-2.101958
C	2.303351	4.950838	-1.072753
C	2.585427	4.598111	0.244009
C	3.056323	3.316918	0.559892
C	3.318783	1.773699	-2.953414
C	4.832523	1.683225	-3.169162
C	3.276227	2.903240	2.004265
C	3.807733	4.032137	2.880619
C	2.541389	-1.493385	3.024979
C	1.066390	-1.455310	3.414105
C	4.352610	-3.331388	-1.368799
C	3.807021	-4.267368	-2.443186
B	2.780412	-0.111527	-0.057758
O	1.455595	-0.132426	-0.278601
Al	-0.079221	-1.039225	-0.555366
C	-0.081831	-1.073672	-2.892342
C	1.284319	-1.554372	-3.294901
O	-1.391842	0.028139	0.078262
B	-2.715086	0.264041	0.091805
N	-3.343998	1.586503	0.190220
C	-2.724633	2.817479	0.486172
C	-2.364988	3.097234	1.824495
C	-1.674427	4.284906	2.095867
C	-1.372418	5.187762	1.079128
C	-1.763705	4.918158	-0.230684
C	-2.438723	3.733841	-0.551972
C	-2.696393	2.121043	2.935693
C	-3.347049	2.806306	4.134438
C	-2.855186	3.438155	-1.978909
C	-4.174861	4.139283	-2.306961
C	-4.733881	1.433245	0.181905
C	-5.031546	0.119503	0.075643
N	-3.858597	-0.635780	0.022510
C	-3.910398	-2.052537	-0.010359
C	-3.538247	-2.782916	1.139935
C	-3.607466	-4.175921	1.083163
C	-4.047022	-4.830272	-0.064029
C	-4.418114	-4.096810	-1.182764
C	-4.347552	-2.700391	-1.181703
C	-3.099100	-2.061965	2.402455
C	-2.338228	-2.942795	3.385765
C	-4.709915	-1.925270	-2.433666
C	-3.921724	-2.409403	-3.649853
C	-1.465496	1.315411	3.350453
C	-1.782040	3.790227	-3.006777
C	-6.214181	-1.970496	-2.703347
C	-4.292868	-1.409220	3.106063
C	-0.116423	-2.853668	0.167212
C	-0.671907	-2.892188	-1.244101
C	-0.392431	-4.052226	-2.153018
C	2.613080	2.082103	-4.267999
C	1.992915	2.313433	2.592579
C	3.456043	-1.492285	4.249190
C	5.876515	-3.451146	-1.287840
C	-0.287584	0.279573	-2.614498
K	0.181429	2.215433	-0.174317
H	-0.912483	-1.634882	-3.326490
H	-1.375892	4.509919	3.122845
H	-6.009260	-0.355728	0.043983
H	-3.035541	2.353095	-2.031405
H	-0.834516	6.110840	1.308822
H	-1.529502	5.636102	-1.019921
H	-3.427995	1.410800	2.524867
H	-3.309176	-4.765923	1.950615
H	-5.405580	2.284096	0.269637
H	-2.408263	-1.265663	2.082044
H	-4.828278	-0.707121	2.452135
H	-3.959210	-0.854396	3.998008
H	-5.009545	-2.177295	3.438715
H	-1.607231	4.875866	-3.069348
H	-2.090490	3.451638	-4.006967
H	-0.816392	3.306909	-2.790353
H	-2.987458	-3.712883	3.832923
H	-1.949153	-2.327847	4.211521
H	-1.482532	-3.442689	2.909808
H	-4.751170	-4.617789	-2.083827
H	-4.431046	-0.875443	-2.257452
H	-4.971567	3.834999	-1.613611
H	-4.502789	3.891641	-3.328447
H	-4.066577	5.233943	-2.239608
H	-1.032711	0.778179	2.494183
H	-1.732807	0.562743	4.107972
H	-0.690337	1.965630	3.786874
H	-4.092483	-5.921772	-0.084825
H	-6.552538	-3.003602	-2.883682
H	-6.471124	-1.372985	-3.592453
H	-6.790315	-1.577741	-1.852574
H	-2.650945	3.485361	4.652604
H	-3.672466	2.052942	4.868084
H	-4.229633	3.390775	3.833996
H	-1.287352	0.714788	-2.677218
H	0.568944	0.955872	-2.581490
H	-2.840475	-2.395231	-3.452545
H	-4.120994	-1.765954	-4.521169
H	-4.194121	-3.438793	-3.930873
H	2.309396	4.329950	-3.131753
H	5.795731	-1.157885	0.958139
H	4.028274	2.099997	2.002830
H	1.922615	5.949264	-1.301464
H	2.424080	5.326103	1.041614
H	2.978576	0.784045	-2.612410
H	3.649460	-5.697152	-0.193532
H	5.742774	1.461292	0.238356
H	4.114048	-2.297865	-1.666260
H	6.305965	-2.729021	-0.580144
H	6.334805	-3.270362	-2.273269
H	6.168474	-4.461198	-0.957715
H	1.178086	3.057620	2.589177
H	2.146161	1.999829	3.636515
H	1.664178	1.418551	2.042862
H	4.133212	-5.307461	-2.285104
H	4.170899	-3.961995	-3.436279
H	2.708654	-4.260078	-2.464640
H	2.133229	-4.137292	3.504841
H	2.758631	-0.576782	2.458236
H	4.703673	4.496966	2.442442
H	4.077875	3.642933	3.873819
H	3.058392	4.824214	3.039178
H	1.522575	2.166059	-4.148102
H	2.799521	1.270422	-4.986677
H	2.986175	3.012120	-4.726343

H	2.645786	-6.064919	2.035961
H	3.301289	-2.392017	4.866396
H	3.256081	-0.613998	4.884383
H	4.516198	-1.466500	3.954493
H	5.239632	2.658460	-3.481092
H	5.061902	0.950934	-3.958759
H	5.354520	1.365767	-2.256332
H	0.428243	-1.469114	2.517138
H	0.842805	-0.548972	3.999043
H	0.789442	-2.322332	4.034443
H	-0.775912	-3.259830	0.949950
H	0.873718	-3.329240	0.224970
H	-1.750508	-2.659462	-1.243327
H	-0.988216	-4.936023	-1.854695
H	-0.637965	-3.848704	-3.212239
H	0.665098	-4.358015	-2.101797
H	1.505485	-1.212404	-4.321590
H	2.064277	-1.138762	-2.639786
H	1.366748	-2.645717	-3.273247

****

152

meso-3,4-isomer K[(C4H6Me2)Al(OBoryl)2]

C	-4.521405	-2.877726	-0.061766
C	-4.054489	-1.768112	0.669880
C	-3.842842	-1.846216	2.063291
C	-4.138889	-3.047587	2.709147
C	-4.640676	-4.139295	2.006407
C	-4.822798	-4.053373	0.633774
N	-3.870068	-0.528517	0.005116
C	-4.989881	0.144538	-0.491027
C	-4.619687	1.308569	-1.065998
N	-3.232204	1.439893	-0.969825
C	-2.549344	2.571522	-1.452628
C	-2.644570	3.795169	-0.744682
C	-1.928312	4.898981	-1.219934
C	-1.128658	4.799507	-2.358607
C	-1.042752	3.591860	-3.043942
C	-1.749179	2.461549	-2.610348
C	-3.436472	3.870597	0.549514
C	-2.599270	3.348843	1.721655
C	-1.664475	1.166806	-3.390584
C	-0.225320	0.699349	-3.584415
C	-3.354650	-0.634853	2.836296
C	-4.489172	0.373336	3.038183
C	-4.676274	-2.841537	-1.570716
C	-6.143244	-2.931860	-1.991658
B	-2.681724	0.267032	-0.278700
O	-1.382038	0.091381	0.022813
Al	0.001140	-1.096012	0.009274
C	-0.028304	-2.442662	-1.438294
C	0.618905	-3.641367	-0.716990
C	0.510175	-4.948822	-1.491199
O	1.389570	0.098777	-0.021811
B	2.694545	0.190837	0.293923
N	3.320297	1.347870	0.942820
C	2.702395	2.533950	1.379625
C	2.833227	3.711726	0.604074
C	2.166262	4.866836	1.026075
C	1.381794	4.861145	2.179970
C	1.264667	3.698595	2.935441
C	1.923557	2.520570	2.556677
C	3.608774	3.680770	-0.701094
C	2.727598	3.140374	-1.831601
C	1.796261	1.271978	3.403742
C	0.347933	0.803310	3.518454
N	3.823842	-0.703317	0.067612
C	4.983680	-0.093227	0.550977
C	4.693123	1.120649	1.067363
C	3.893541	-1.979892	-0.546216
C	3.680728	-2.086989	-1.938646
C	3.791064	-3.347592	-2.527860
C	4.118460	-4.469852	-1.772400
C	4.328837	-4.347443	-0.406532
C	4.213752	-3.108997	0.234147
C	3.373144	-0.852859	-2.768201
C	4.617969	0.023900	-2.933188
C	4.441612	-3.017982	1.731593
C	5.925545	-3.168856	2.073640
C	2.775155	-1.167401	-4.133697
C	3.610448	-4.036143	2.510944
C	4.215274	5.025813	-1.082334
C	2.431095	1.480737	4.778347
C	0.288097	-2.403325	1.477194
C	0.133617	-3.760484	0.755423
H	0.775591	-4.529602	1.230588
C	-3.980886	5.261581	0.851629
C	-2.401122	1.299151	-4.723803
C	-2.712057	-0.984572	4.172628
C	-3.848309	-3.936015	-2.244632
K	0.043427	2.358517	0.006137
H	-1.987652	5.851211	-0.689608
H	-5.987485	-0.274214	-0.383589
H	-2.183989	0.398816	-2.800580
H	-0.574303	5.671778	-2.713814
H	-0.419381	3.522019	-3.938885
H	-4.295486	3.190250	0.446340
H	-3.980156	-3.135185	3.784791
H	-5.234194	2.054538	-1.565624
H	-2.576423	-0.159222	2.217719
H	-4.909279	0.714145	2.080962
H	-4.129280	1.257482	3.588308
H	-5.304564	-0.078634	3.625435
H	0.365417	1.412388	-4.182157
H	-0.207548	-0.266162	-4.109487
H	0.287880	0.545266	-2.623281
H	-3.445397	-1.388464	4.888679
H	-2.280823	-0.081710	4.629843
H	-1.905752	-1.723311	4.052815
H	-5.189434	-4.922815	0.082471
H	-4.284838	-1.873308	-1.917222
H	-3.451006	1.590650	-4.570463
H	-2.388801	0.341458	-5.266645
H	-1.930973	2.058142	-5.370156
H	-2.310443	2.296325	1.578330
H	-3.173613	3.392726	2.658944
H	-1.691187	3.959564	1.865990
H	-4.872374	-5.067835	2.533478
H	-6.589410	-3.887395	-1.672209
H	-6.238828	-2.868422	-3.087110
H	-6.744709	-2.122417	-1.552326
H	-3.180052	5.976111	1.101263
H	-4.656086	5.221020	1.719399
H	-4.546391	5.670020	0.000616
H	-1.061522	-2.688350	-1.748938
H	0.521640	-2.215980	-2.366299
H	-2.790715	-3.875842	-1.951511
H	-3.905149	-3.840662	-3.340424
H	-4.211889	-4.941826	-1.981420
H	2.252341	5.785078	0.442025
H	5.950475	-0.584151	0.474577
H	2.360966	0.478746	2.893419
H	0.864976	5.771985	2.492525
H	0.654452	3.702880	3.842392
H	4.434586	2.964662	-0.570701
H	3.618420	-3.460648	-3.598653
H	5.355266	1.843564	1.539176
H	2.619031	-0.278020	-2.205897
H	5.023578	0.352048	-1.966000
H	4.381646	0.922216	-3.525830
H	5.409041	-0.529387	-3.464348
H	-0.288878	1.553637	4.015204

H	0.291253	-0.123549	4.107032
H	-0.088669	0.575657	2.534555
H	3.504409	-1.662602	-4.794646
H	2.468261	-0.235976	-4.632444
H	1.890796	-1.816256	-4.052980
H	4.572562	-5.234508	0.182701
H	4.119917	-2.014636	2.050275
H	3.484862	1.784234	4.688212
H	2.393352	0.548953	5.363310
H	1.904321	2.258187	5.355204
H	2.380994	2.116940	-1.622573
H	3.288870	3.098415	-2.777036
H	1.852111	3.791174	-1.998415
H	4.196708	-5.447375	-2.253783
H	6.295664	-4.164274	1.779551
H	6.089265	-3.056491	3.157115
H	6.545897	-2.421337	1.558510
H	3.445892	5.764918	-1.358015
H	4.873618	4.907696	-1.955959
H	4.813505	5.449190	-0.261494
H	2.541436	-3.948181	2.273662
H	3.735055	-3.878779	3.593854
H	3.922687	-5.069757	2.293823
H	-0.333612	-2.366579	2.386898
H	1.333660	-2.267039	1.814622
C	-1.300804	-4.254180	0.863867
H	1.695278	-3.414812	-0.638088
H	1.019371	-4.862323	-2.463866
H	0.973866	-5.788714	-0.945191
H	-0.539952	-5.216290	-1.693822
H	-1.457189	-5.215739	0.351773
H	-1.589022	-4.388121	1.917104
H	-2.006906	-3.527953	0.434850

****

152

TS2 propene (to 2,4-isomer)

C	-3.263412	-2.748545	2.023954
C	-3.584315	-2.382815	0.704633
C	-3.742244	-3.348436	-0.312345
C	-3.570963	-4.692348	0.026693
C	-3.261037	-5.070657	1.330104
C	-3.109803	-4.106639	2.318477
N	-3.769052	-1.011141	0.395599
C	-4.995129	-0.382260	0.616604
C	-4.914821	0.924654	0.277740
N	-3.622708	1.194851	-0.183236
C	-3.192103	2.460526	-0.621995
C	-2.981215	3.489703	0.324217
C	-2.480441	4.718088	-0.125342
C	-2.185108	4.918326	-1.471694
C	-2.410394	3.900315	-2.397184
C	-2.927526	2.661014	-1.998690
C	-3.223587	3.224079	1.799989
C	-2.061573	2.430873	2.402218
C	-3.224883	1.561425	-3.004348
C	-2.587958	1.784097	-4.370848
C	-4.101826	-2.929272	-1.727764
C	-5.603772	-2.664387	-1.864621
C	-3.039362	-1.704094	3.098682
C	-3.973879	-1.899031	4.290579
B	-2.816036	-0.024420	-0.083879
O	-1.499407	-0.091352	-0.329877
Al	-0.001269	-1.056498	-0.593104
C	-0.020698	-2.823420	0.255398
C	-0.288598	-3.030292	-1.219274
C	0.542971	-4.012514	-1.989395
O	1.352157	-0.012248	-0.012630
B	2.674350	0.173765	0.120080
N	3.331146	1.464758	0.374416
C	2.723299	2.659569	0.812936
C	2.627907	3.776061	-0.050993
C	1.943503	4.913898	0.395333
C	1.368983	4.955492	1.663280
C	1.504358	3.866640	2.520279
C	2.192195	2.713897	2.123440
C	3.278241	3.765580	-1.419729
C	2.351027	4.263717	-2.526877
C	2.374524	1.563973	3.092617
C	1.058446	0.849830	3.389977
C	4.714833	1.262178	0.425063
C	4.979452	-0.046747	0.219835
N	3.790187	-0.758319	0.039041
C	3.805954	-2.174551	-0.059433
C	3.413288	-2.949001	1.056321
C	3.498402	-4.338984	0.957698
C	3.965805	-4.952948	-0.200667
C	4.342453	-4.179916	-1.289893
C	4.262112	-2.783599	-1.244072
C	2.941733	-2.281072	2.335767
C	4.129524	-1.739619	3.136149
C	4.669979	-1.967090	-2.454207
C	6.184822	-1.993091	-2.661081
C	4.578742	4.571528	-1.397704
C	3.069271	2.022075	4.373918
C	3.940117	-2.417724	-3.718819
C	2.076193	-3.173166	3.218561
C	-0.098377	-1.187191	-2.854002
H	-1.146230	-1.428083	-3.059051
C	-3.492474	4.484186	2.613064
C	-4.734599	1.352849	-3.159640
C	-3.651128	-3.919216	-2.797510
C	-1.571944	-1.670142	3.524539
C	0.278992	0.153939	-2.702814
K	-0.106940	2.226398	-0.346978
H	-2.306737	5.526041	0.587428
H	-5.847926	-0.932914	1.007938
H	-2.803942	0.631675	-2.586235
H	-1.784877	5.878859	-1.805505
H	-2.187841	4.078263	-3.450299
H	-4.116031	2.583583	1.871127
H	-2.850800	-4.411025	3.335814
H	-5.693417	1.684505	0.292927
H	-3.274681	-0.726226	2.653468
H	-5.029049	-1.886923	3.977424
H	-3.828639	-1.097465	5.032232
H	-3.787549	-2.857613	4.800785
H	-3.037362	2.643238	-4.894608
H	-2.749356	0.897953	-5.002000
H	-1.501562	1.944144	-4.308306
H	-1.272406	-2.614920	4.006346
H	-1.394657	-0.855907	4.245412
H	-0.914396	-1.521132	2.655246
H	-3.672752	-5.460356	-0.741727
H	-3.581952	-1.973758	-1.912780
H	-5.214385	1.091372	-2.207322
H	-4.931889	0.533854	-3.868727
H	-5.212993	2.264057	-3.553152
H	-1.892164	1.481851	1.872485
H	-2.263270	2.178011	3.454363
H	-1.131324	3.022337	2.382134
H	-3.127039	-6.127749	1.572022
H	-6.177161	-3.581140	-1.652721
H	-5.846886	-2.341263	-2.889597
H	-5.945458	-1.879319	-1.177260
H	-2.597065	5.121534	2.694560
H	-3.787809	4.214244	3.638017
H	-4.301168	5.088911	2.175565
H	-0.523072	0.897989	-2.680515
C	1.668048	0.631700	-2.990680
H	-2.585828	-4.175096	-2.702721
H	-3.809182	-3.487850	-3.797850

H	-4.231070	-4.854943	-2.756126
H	1.857121	5.781820	-0.262870
H	5.944186	-0.548507	0.197725
H	3.038772	0.838415	2.603167
H	0.828461	5.847146	1.990504
H	1.078694	3.915620	3.525883
H	3.540673	2.719302	-1.637563
H	4.696359	-4.667551	-2.201537
H	5.407810	2.077168	0.620870
H	4.378693	-0.925273	-2.251763
H	6.720696	-1.620157	-1.776045
H	6.472894	-1.367339	-3.520705
H	6.539528	-3.017817	-2.856984
H	0.338076	1.517288	3.889360
H	1.229342	-0.009780	4.055453
H	0.599142	0.463368	2.469141
H	4.250726	-3.427324	-4.030583
H	4.156392	-1.733764	-4.554361
H	2.851314	-2.439761	-3.567841
H	3.189876	-4.957120	1.801878
H	2.311956	-1.431521	2.028644
H	4.025929	2.519518	4.154706
H	3.277162	1.157230	5.022511
H	2.446673	2.724313	4.951398
H	1.405863	3.699464	-2.567088
H	2.833955	4.149418	-3.508919
H	2.099791	5.329269	-2.407344
H	4.025166	-6.042563	-0.254891
H	4.793141	-2.563566	3.444218
H	3.780679	-1.231386	4.049528
H	4.726435	-1.021372	2.556622
H	4.384102	5.632012	-1.170024
H	5.084096	4.521270	-2.374676
H	5.273938	4.188939	-0.636654
H	1.229739	-3.598889	2.661638
H	1.665491	-2.583248	4.052324
H	2.655425	-3.998440	3.663384
H	-0.808578	-3.192123	0.926758
H	0.961024	-3.218769	0.544067
H	-1.360583	-3.123632	-1.434703
H	0.312045	-5.052646	-1.687704
H	0.379397	-3.954162	-3.080729
H	1.618661	-3.864930	-1.799643
H	0.607068	-1.886034	-3.312614
H	1.767436	1.061849	-4.007103
H	2.033889	1.397903	-2.287822
H	2.387022	-0.197093	-2.925814

****

152

2,4-isomer K[(C4H6Me2)Al(OBoryl)2]

C	3.814606	-3.485077	0.140809
C	3.642182	-2.300875	-0.604422
C	3.406103	-2.337739	-1.997589
C	3.327182	-3.584604	-2.620292
C	3.496344	-4.762077	-1.897755
C	3.742994	-4.708913	-0.532902
N	3.751488	-1.041781	0.041399
C	4.982955	-0.565199	0.496826
C	4.844251	0.682252	0.998887
N	3.504035	1.066335	0.894790
C	3.033343	2.336737	1.280785
C	3.174807	3.428976	0.392855
C	2.622772	4.662685	0.756780
C	1.948963	4.816726	1.968081
C	1.837905	3.740627	2.844482
C	2.387040	2.490830	2.526309
C	3.834738	3.234922	-0.959588
C	2.793314	2.811278	-1.998352
C	2.254756	1.325073	3.483962
C	0.843561	0.738863	3.440438
C	3.286694	-1.048821	-2.794050
C	4.648738	-0.362603	-2.936301
C	4.116233	-3.461399	1.628128
C	5.590930	-3.780746	1.887443
B	2.742526	-0.019189	0.275834
O	1.428393	0.024061	-0.012111
Al	-0.011978	-1.086047	-0.126409
C	-0.095425	-2.506684	1.245607
C	-0.704948	-3.651274	0.414408
C	-0.614810	-5.018970	1.077752
O	-1.321970	0.179043	-0.084027
B	-2.631498	0.377635	-0.307989
N	-3.190054	1.593171	-0.906256
C	-2.498739	2.773460	-1.242545
C	-2.540656	3.880675	-0.359568
C	-1.805876	5.027349	-0.682250
C	-1.036578	5.085579	-1.843047
C	-1.000486	3.991758	-2.702137
C	-1.725954	2.825253	-2.424342
C	-3.280258	3.797337	0.963915
C	-2.319621	3.374880	2.079682
C	-1.673840	1.661867	-3.391112
C	-0.250059	1.141159	-3.579572
N	-3.817328	-0.418906	0.004778
C	-4.946066	0.302400	-0.393990
C	-4.583889	1.491190	-0.924880
C	-3.935877	-1.654787	0.695301
C	-3.525361	-1.722982	2.047497
C	-3.620328	-2.946516	2.712165
C	-4.142198	-4.070919	2.081930
C	-4.584028	-3.980112	0.770842
C	-4.487548	-2.783836	0.051246
C	-3.069096	-0.475273	2.783415
C	-4.263239	0.448500	3.044606
C	-5.001624	-2.747950	-1.376408
C	-6.531210	-2.674887	-1.407845
C	-2.342413	-0.758749	4.091972
C	-4.530429	-3.947053	-2.200744
C	-4.008757	5.087900	1.325362
C	-2.318074	2.035361	-4.725985
C	-0.163342	-2.301038	-1.703070
C	-0.055594	-3.658319	-0.983328
H	-0.488298	-4.477739	-1.593731
C	4.615944	4.455918	-1.431817
C	2.659525	1.699019	4.907835
C	2.654602	-1.223159	-4.170002
C	3.225348	-4.405275	2.433409
K	0.195001	2.357410	0.123765
H	2.714220	5.515977	0.081521
H	5.886243	-1.163763	0.411836
H	2.946119	0.545941	3.130060
H	1.518226	5.785470	2.233518
H	1.321471	3.873418	3.798341
H	4.544529	2.400211	-0.855630
H	3.129880	-3.641756	-3.691401
H	5.595450	1.332614	1.441849
H	2.624906	-0.384421	-2.215161
H	5.099332	-0.126901	-1.962451
H	4.549570	0.578027	-3.501522
H	5.348362	-1.011280	-3.487391
H	0.089899	1.491990	3.725203
H	0.749719	-0.104765	4.139836
H	0.596501	0.352243	2.440905
H	3.305614	-1.796603	-4.849172
H	2.493182	-0.237434	-4.632617
H	1.681728	-1.732732	-4.115214
H	3.871039	-5.637685	0.027831
H	3.921643	-2.437526	1.982927
H	3.667813	2.138719	4.937952
H	2.658696	0.804169	5.548518
H	1.961634	2.421737	5.360120

H	2.313566	1.860362	-1.722959
H	3.259333	2.660108	-2.983726
H	2.013951	3.583610	-2.117246
H	3.428238	-5.727335	-2.404859
H	5.830541	-4.805543	1.560863
H	5.823676	-3.706832	2.961605
H	6.263234	-3.098795	1.347450
H	3.955312	5.302304	-1.679227
H	5.180281	4.212073	-2.344456
H	5.332889	4.797352	-0.670103
H	0.934651	-2.781096	1.544206
H	-0.662706	-2.341364	2.174533
H	2.160045	-4.193508	2.270229
H	3.434109	-4.297894	3.509392
H	3.403706	-5.460143	2.171644
H	-1.825490	5.886660	-0.008227
H	-5.947761	-0.094637	-0.256324
H	-2.270211	0.851578	-2.948771
H	-0.466914	5.988071	-2.078083
H	-0.401035	4.041964	-3.614793
H	-4.036293	3.004112	0.871366
H	-3.287430	-3.025435	3.747432
H	-5.212234	2.273152	-1.346194
H	-2.357127	0.045558	2.123160
H	-4.751935	0.766796	2.113042
H	-3.945419	1.350655	3.590336
H	-5.015009	-0.067750	3.662677
H	0.423283	1.917387	-3.977609
H	-0.237618	0.299825	-4.287709
H	0.172144	0.765682	-2.635134
H	-3.013558	-1.201841	4.844874
H	-1.958166	0.180476	4.516962
H	-1.490033	-1.438348	3.948435
H	-5.000235	-4.863662	0.282440
H	-4.604698	-1.835718	-1.848042
H	-3.351515	2.385785	-4.584937
H	-2.342759	1.162636	-5.396521
H	-1.758167	2.834636	-5.238296
H	-1.940291	2.353576	1.919274
H	-2.830206	3.368708	3.054132
H	-1.465449	4.069251	2.159949
H	-4.204902	-5.020245	2.618790
H	-6.970309	-3.579500	-0.957382
H	-6.896732	-2.603339	-2.444529
H	-6.921958	-1.811672	-0.850913
H	-3.313062	5.910831	1.554580
H	-4.630670	4.933015	2.219952
H	-4.666003	5.419824	0.507794
H	-3.446161	-4.097669	-2.118588
H	-4.774531	-3.796855	-3.263869
H	-5.027677	-4.876845	-1.882922
H	0.672505	-2.218569	-2.414191
C	-1.469631	-2.159766	-2.474121
H	1.011174	-3.913065	-0.850631
H	-1.779931	-3.425587	0.280966
H	-1.075307	-5.006977	2.078321
H	-1.123788	-5.795677	0.481634
H	0.438251	-5.327100	1.192867
H	-1.624558	-2.980801	-3.201304
H	-2.344612	-2.161517	-1.806021
H	-1.517973	-1.217394	-3.041058

****

152

TS2 propene (to 2,5-isomer)

C	-4.504727	-1.750781	1.761572
C	-4.251988	-1.620572	0.379695
C	-4.303012	-2.737282	-0.481341
C	-4.582147	-3.989111	0.075306
C	-4.810008	-4.135392	1.438366
C	-4.775242	-3.023464	2.272163
N	-3.986362	-0.334400	-0.162342
C	-5.018681	0.430291	-0.710080
C	-4.525283	1.565888	-1.251854
N	-3.138369	1.582882	-1.081781
C	-2.307723	2.589416	-1.608325
C	-2.307982	3.873665	-1.016808
C	-1.452500	4.850772	-1.541132
C	-0.613052	4.561863	-2.614220
C	-0.615913	3.289279	-3.182190
C	-1.455592	2.279798	-2.696015
C	-3.160089	4.145116	0.211412
C	-3.513380	5.614867	0.401689
C	-1.470021	0.891065	-3.309279
C	-2.732683	0.671732	-4.146278
C	-4.112805	-2.592486	-1.979293
C	-3.359338	-3.760059	-2.611036
C	-4.543249	-0.532937	2.663786
C	-4.035682	-0.805887	4.076445
B	-2.726550	0.377657	-0.358711
O	-1.469473	0.127586	0.040199
Al	-0.175427	-1.125588	0.219638
C	-0.192701	-0.975997	2.546353
C	-0.871665	0.295748	2.955071
O	1.393596	-0.210354	0.269676
B	2.701673	-0.236279	0.577067
N	3.494923	0.902836	1.058358
C	3.106299	2.256523	1.158515
C	3.291677	3.101963	0.040448
C	2.842006	4.426412	0.117429
C	2.232817	4.905583	1.273675
C	2.084668	4.072739	2.381556
C	2.523551	2.742252	2.353562
C	3.906399	2.562032	-1.237898
C	4.752119	3.591418	-1.979852
C	2.402481	1.846226	3.572616
C	3.769866	1.612836	4.222594
C	4.835069	0.512940	1.153384
C	4.944540	-0.787813	0.806193
N	3.693122	-1.299460	0.458362
C	3.608840	-2.577653	-0.153736
C	4.057049	-2.722539	-1.481208
C	4.052307	-3.999820	-2.050717
C	3.591430	-5.097124	-1.336457
C	3.129822	-4.934973	-0.033192
C	3.138996	-3.683144	0.586626
C	4.536347	-1.535753	-2.295567
C	3.741992	-1.376403	-3.590437
C	2.700124	-3.512266	2.029360
C	1.821805	-4.646898	2.543143
C	2.824257	1.982808	-2.151562
C	1.422184	2.363872	4.619172
C	3.904016	-3.327570	2.957784
C	6.037619	-1.618457	-2.570484
C	-0.431450	-2.318505	-1.334406
C	-0.732356	-3.102961	-0.071655
H	-1.715084	-3.573759	0.085372
C	-0.860685	-2.137428	2.133960
C	-2.486068	3.579535	1.465210
C	-0.222858	0.560612	-4.118273
C	-5.459633	-2.393263	-2.680324
C	-5.953235	0.061974	2.705746
K	0.229959	2.206967	0.056602
H	-0.450142	-3.121907	2.358466
H	2.966798	5.090691	-0.740015
H	5.832827	-1.415726	0.791334
H	2.040054	0.870592	3.209313
H	1.881563	5.939498	1.318235
H	1.623333	4.468787	3.287349
H	4.571269	1.732864	-0.950960
H	4.398687	-4.131378	-3.078892
H	5.610088	1.200267	1.485777

H	4.360488	-0.634444	-1.690562
H	6.613230	-1.681556	-1.635283
H	6.382831	-0.729459	-3.122295
H	6.284724	-2.505212	-3.176131
H	1.809932	3.261781	5.126888
H	1.264156	1.597339	5.391838
H	0.439101	2.610327	4.193299
H	3.865502	-2.245523	-4.255546
H	4.084411	-0.488325	-4.145477
H	2.668263	-1.265724	-3.384355
H	2.761407	-5.804528	0.512539
H	2.108866	-2.583382	2.071049
H	4.473546	1.125652	3.535964
H	3.662636	0.963914	5.105510
H	4.209397	2.567107	4.554797
H	2.289227	1.147358	-1.674036
H	3.264643	1.590033	-3.079956
H	2.094209	2.758586	-2.436767
H	3.579934	-6.086760	-1.799160
H	4.551409	-4.218829	2.930254
H	3.568481	-3.185052	3.997396
H	4.509127	-2.455077	2.677614
H	4.140026	4.397663	-2.415007
H	5.284749	3.108697	-2.813087
H	5.501523	4.053194	-1.319466
H	0.838582	-1.097309	2.898701
H	0.972255	-4.848897	1.874647
H	1.420360	-4.390304	3.535122
H	2.393168	-5.581995	2.657731
H	-1.437475	5.850231	-1.102748
H	-6.051270	0.091418	-0.672343
H	-1.492871	0.176596	-2.471670
H	0.048399	5.335167	-3.012774
H	0.045930	3.076920	-4.023165
H	-4.101454	3.589055	0.083770
H	-4.963450	-3.146762	3.340394
H	-5.052752	2.359768	-1.776455
H	-3.887641	0.222825	2.203568
H	-6.298978	0.351488	1.703756
H	-5.978226	0.959566	3.344462
H	-6.670230	-0.667299	3.115831
H	-0.139445	1.173489	-5.030590
H	-0.260468	-0.491791	-4.433642
H	0.695475	0.688958	-3.526847
H	-4.737578	-1.435117	4.646337
H	-3.928321	0.139332	4.630436
H	-3.057590	-1.308068	4.070602
H	-4.614814	-4.867422	-0.572127
H	-3.518372	-1.679177	-2.138602
H	-3.646935	0.820070	-3.554889
H	-2.750863	-0.356850	-4.538850
H	-2.758475	1.363430	-5.003924
H	-2.314409	2.496567	1.377033
H	-3.117606	3.732360	2.353631
H	-1.523925	4.085995	1.660396
H	-5.018368	-5.124049	1.854539
H	-6.110163	-3.269576	-2.527194
H	-5.313735	-2.264379	-3.764694
H	-5.989824	-1.507268	-2.306052
H	-2.631920	6.222563	0.662426
H	-4.234177	5.725660	1.225598
H	-3.965638	6.045262	-0.504465
H	-2.425051	-3.977666	-2.075623
H	-3.104194	-3.520664	-3.654924
H	-3.972196	-4.675498	-2.632217
C	0.487815	-2.968943	-2.341687
H	-1.369824	-1.985128	-1.807814
H	0.080151	-3.799507	0.187261
H	-1.950760	-2.128424	2.040927
H	-0.957725	0.388062	4.055056
H	-0.329495	1.200563	2.625991
H	-1.884397	0.359613	2.540908
H	0.061796	-3.896933	-2.775457
H	0.716425	-2.293758	-3.183159
H	1.450317	-3.248773	-1.889707

****

152

2,5-isomer K[(C4H6Me2)Al(OBoryl)2]

C	-4.291862	-3.070432	0.051869
C	-3.955815	-1.810033	0.587867
C	-3.843653	-1.626581	1.985437
C	-4.024206	-2.729428	2.821989
C	-4.323803	-3.984192	2.302493
C	-4.465524	-4.144436	0.932162
N	-3.782963	-0.685298	-0.264153
C	-4.880131	-0.070206	-0.873689
C	-4.504338	1.087315	-1.461184
N	-3.130284	1.270546	-1.274715
C	-2.499652	2.511766	-1.502215
C	-2.677368	3.558651	-0.565274
C	-2.012149	4.772488	-0.775146
C	-1.178604	4.954427	-1.876699
C	-1.006737	3.917385	-2.789267
C	-1.661238	2.689175	-2.624889
C	-3.528393	3.374006	0.676755
C	-4.687148	4.367835	0.716812
C	-1.455098	1.583540	-3.635890
C	-1.942717	2.001330	-5.022306
C	-3.623565	-0.243816	2.570867
C	-3.008426	-0.252103	3.965034
C	-4.528062	-3.289693	-1.429912
C	-3.790198	-4.515049	-1.968266
B	-2.600874	0.141603	-0.501992
O	-1.324428	0.020156	-0.093925
Al	0.078967	-1.140743	-0.191451
C	0.062773	-2.252589	-1.856140
C	-1.231512	-2.175038	-2.656421
O	1.437898	0.058851	0.000877
B	2.719658	0.044578	0.410286
N	3.373819	1.137188	1.127846
C	2.817395	2.382749	1.477997
C	3.017185	3.496175	0.627560
C	2.404703	4.710395	0.957592
C	1.609272	4.826224	2.097198
C	1.423802	3.725921	2.928877
C	2.023868	2.492345	2.639942
C	3.799479	3.344589	-0.663299
C	4.618362	4.578884	-1.024162
C	1.813261	1.311226	3.562974
C	2.451853	1.566097	4.928010
C	4.708649	0.795697	1.361875
C	4.943192	-0.431641	0.845666
N	3.783086	-0.938807	0.253470
C	3.808731	-2.159146	-0.472883
C	3.956782	-3.382736	0.212575
C	4.044433	-4.558573	-0.540327
C	3.967648	-4.531727	-1.925746
C	3.804400	-3.318929	-2.588203
C	3.731689	-2.115974	-1.883732
C	4.041345	-3.454751	1.726182
C	3.145371	-4.544850	2.314974
C	3.623094	-0.786875	-2.611032
C	3.113562	-0.903235	-4.042623
C	2.852617	2.956182	-1.802251
C	0.335448	0.950826	3.691415
C	4.962955	-0.045156	-2.593122
C	5.485560	-3.660637	2.190598
C	0.228024	-2.661286	1.070272
C	-0.300619	-3.786191	0.169250
C	0.286347	-3.652446	-1.243761
H	-0.072167	-4.791305	0.579817

C	-2.682474	3.447620	1.949826
C	-0.000680	1.116300	-3.667700
C	-4.945268	0.530547	2.578225
C	-6.025752	-3.401102	-1.728114
K	0.083688	2.300705	0.089041
H	-0.110251	-4.449554	-1.904275
H	2.544596	5.578881	0.310430
H	5.872867	-0.994474	0.836304
H	2.327638	0.452708	3.107580
H	1.136662	5.781541	2.338840
H	0.803684	3.824326	3.823728
H	4.498167	2.506191	-0.523020
H	3.734898	-3.312040	-3.676628
H	5.386690	1.457765	1.896171
H	2.887515	-0.184501	-2.054647
H	5.317795	0.143542	-1.570355
H	4.876447	0.925061	-3.108098
H	5.732945	-0.633987	-3.116898
H	-0.257324	1.783786	4.102794
H	0.205691	0.093295	4.367313
H	-0.100302	0.658741	2.723041
H	3.844701	-1.401555	-4.699079
H	2.937655	0.100574	-4.458746
H	2.167659	-1.461672	-4.096259
H	4.158885	-5.515420	-0.026309
H	3.691794	-2.485226	2.114943
H	3.523349	1.795357	4.828631
H	2.351793	0.678210	5.571053
H	1.973242	2.411450	5.448547
H	2.359545	1.992896	-1.603363
H	3.400778	2.844542	-2.749924
H	2.079131	3.728165	-1.956895
H	4.023889	-5.461913	-2.495985
H	5.879044	-4.621075	1.820353
H	5.542105	-3.675496	3.290601
H	6.155558	-2.868052	1.829497
H	3.982108	5.435061	-1.300069
H	5.261407	4.364208	-1.890953
H	5.265051	4.892383	-0.190937
H	1.330111	-2.783857	1.106654
C	-0.304934	-2.720288	2.488639
H	2.110911	-4.456294	1.955603
H	3.132466	-4.471931	3.413558
H	3.510699	-5.552448	2.061495
H	-2.141623	5.586952	-0.057401
H	-5.868592	-0.521773	-0.843645
H	-2.072275	0.736659	-3.303880
H	-0.663482	5.906964	-2.023691
H	-0.353098	4.061874	-3.653422
H	-3.963214	2.366679	0.628354
H	-3.929261	-2.608456	3.901986
H	-5.105040	1.794953	-2.028680
H	-2.916921	0.279292	1.907821
H	-5.365359	0.632290	1.567431
H	-4.807814	1.540848	2.995164
H	-5.687694	0.008634	3.202719
H	0.684455	1.932741	-3.948544
H	0.129488	0.307180	-4.401181
H	0.319368	0.714008	-2.694278
H	-3.708793	-0.641638	4.720790
H	-2.749468	0.774131	4.267609
H	-2.093520	-0.859619	3.999668
H	-4.715510	-5.128256	0.528903
H	-4.145849	-2.404046	-1.958825
H	-2.995396	2.320668	-4.995831
H	-1.860259	1.158671	-5.725730
H	-1.348156	2.834372	-5.431248
H	-1.961208	2.617322	2.010637
H	-3.319573	3.369142	2.843559
H	-2.125466	4.395767	2.023313
H	-4.453146	-4.837692	2.972120
H	-6.460942	-4.278892	-1.223909
H	-6.199755	-3.512870	-2.810047
H	-6.582202	-2.516822	-1.384849
H	-4.334054	5.408248	0.801391
H	-5.337249	4.164496	1.581699
H	-5.300835	4.293472	-0.193049
H	-2.723658	-4.495363	-1.706316
H	-3.869073	-4.555538	-3.065595
H	-4.218488	-5.450601	-1.575421
H	0.903051	-2.035391	-2.535657
H	1.370543	-3.837970	-1.168526
H	-1.406667	-3.731660	0.131396
H	-1.304616	-2.966121	-3.428365
H	-2.119461	-2.279507	-2.016026
H	-1.342083	-1.210255	-3.173897
H	-0.097544	-3.690683	2.982061
H	0.135157	-1.937587	3.128230
H	-1.397307	-2.587911	2.511421

****

References

1. Sarkar, D.; Vasko, P.; Roper, A. F.; Crumpton, A. E.; Roy, M. M. D.; Griffin, L. P.; Bogle, C.; Aldridge, S. Reversible [4 + 1] Cycloaddition of Arenes by a "Naked" Acyclic Aluminyl Compound. *J. Am. Chem. Soc.* **2024,** *146*, 11792-11800.
2. Cosier, J.; Glazer, A. M. A nitrogen-gas-stream cryostat for general X-ray diffraction studies. *J. Appl. Crystallogr*. **1986**, *19*, 105–107.
3. CrysAlisPro, Agilent Technologies, Version 1.171.39.46.
4. Sheldrick, G. M. Crystal structure refinement with SHELXL. *Acta Crystallogr. C* **2015**, *71*, 3–8.
5. Sheldrick, G. M. SHELXT – Integrated space-group and crystal-structure determination. *Acta Crystallogr. A* **2015,** *71*, 3–8.
6. Dolomanov, O. V.; Bourhis, L. J.; Gildea, R. J.; Howard, J. A. K.; Puschmann, H. OLEX2 : a complete structure solution, refinement and analysis program. *J. Appl. Crystallogr.* **2009**, *42*, 339–341.
7. Gaussian 16, Revision C.02, M. J. Frisch, G. W. Trucks, H. B. Schlegel, G. E. Scuseria, M. A. Robb, J. R. Cheeseman, G. Scalmani, V. Barone, G. A. Petersson, H. Nakatsuji, X. Li, M. Caricato, A. V. Marenich, J. Bloino, B. G. Janesko, R. Gomperts, B. Mennucci, H. P. Hratchian, J. V. Ortiz, A. F. Izmaylov, J. L. Sonnenberg, D. Williams-Young, F. Ding, F. Lipparini, F. Egidi, J. Goings, B. Peng, A. Petrone, T. Henderson, D. Ranasinghe, V. G. Zakrzewski, J. Gao, N. Rega, G. Zheng, W. Liang, M. Hada, M. Ehara, K. Toyota, R. Fukuda, J. Hasegawa, M. Ishida, T. Nakajima, Y. Honda, O. Kitao, H. Nakai, T. Vreven, K. Throssell, J. A. Montgomery, Jr., J. E. Peralta, F. Ogliaro, M. J. Bearpark, J. J. Heyd, E. N. Brothers, K. N. Kudin, V. N. Staroverov, T. A. Keith, R. Kobayashi, J. Normand, K. Raghavachari, A. P. Rendell, J. C. Burant, S. S. Iyengar, J. Tomasi, M. Cossi, J. M. Millam, M. Klene, C. Adamo, R. Cammi, J. W. Ochterski, R. L. Martin, K. Morokuma, O. Farkas, J. B. Foresman, D. J. Fox, Gaussian, Inc., Wallingford CT, **2019**.
8. Perdew, J. P.; Burke, K.; Ernzerhof, M. Generalized Gradient Approximation Made Simple. *Phys. Rev. Lett.* **1996**, *77*, 3865-3868.
9. Perdew, J. P. ; Burke, K.; Ernzerhof, M. Generalized Gradient Approximation Made Simple [Phys. Rev. Lett. 77, 3865 (1996)] *Phys. Rev. Lett.* **1997**, *78*, 1396.
10. Adamo, C.; Barone, V. Toward reliable density functional methods without adjustable parameters: The PBE0 model. *J. Chem. Phys.* **1999**, *110*, 6158-6169.
11. Weigend, F.; Ahlrichs, R. Balanced basis sets of split valence, triple zeta valence and quadruple zeta valence quality for H to Rn: Design and assessment of accuracy. *Phys. Chem. Chem. Phys.* **2005**, *7*, 3297-3305.
12. Weigend, F. Accurate Coulomb-fitting basis sets for H to Rn. *Phys. Chem. Chem. Phys.* **2006**, *8*, 1057-1065.
13. Grimme, S.; Ehrlich, S.; Goerigk, L. Effect of the damping function in dispersion corrected density functional theory. *J. Comp. Chem.* **2011**, *32*, 1456-1465.
14. Tomasi, J.; Mennucci, B.; Cammi, R. Quantum Mechanical Continuum Solvation Models. *Chem. Rev.* **2005**, *105*, 2999-3093.
